# Functionalized Cyclic Beta‐Amino Acid Derivatives With Antiviral Potential

**DOI:** 10.1002/cmdc.202500862

**Published:** 2026-03-30

**Authors:** Melinda Nonn, Marta Denel‐Bobrowska, Balázs Volk, Agnieszka B. Olejniczak, Loránd Kiss

**Affiliations:** ^1^ MTA TTK Lendület Artificial Transporter Research Group Institute of Materials and Environmental Chemistry HUN‐REN Research Center for Natural Sciences Hungarian Academy of Sciences Budapest Hungary; ^2^ National Drug Research and Development Laboratory HUN‐REN Research Centre for Natural Sciences Budapest Hungary; ^3^ Institute of Medical Biology Polish Academy of Sciences Łódź Poland; ^4^ Egis Pharmaceuticals Plc. Directorate of Drug Substance Development Budapest Hungary; ^5^ Institute of Organic Chemistry Stereochemistry Research Group HUN‐REN Research Centre for Natural Sciences Budapest Hungary

**Keywords:** antiviral property, cyclic amino acids, drug design, functionalization, stereocenter

## Abstract

Selected highly functionalized cyclic beta (β)‐amino acid derivatives have been synthesized according to modified and improved literature synthetic protocols and subjected to various antiviral studies. The model compounds were regio‐ and stereoisomers of azido‐functionalized β‐amino esters, orthogonally protected diamino esters, an oxirane‐fused cyclic β‐amino ester, 1,2,3‐triazole‐substituted β‐amino esters, mono‐ and difluorinated cyclic β‐amino esters, mono and dihydroxylated cyclic β‐amino esters, dihydroxylated lactams, and β‐amino esters with piperidine and azepane skeletons. Formed compounds have been investigated and their anti‐HCMV (Human cytomegalovirus), anti‐HRV8 (Human rhinovirus 8), and anti‐HSV‐1 (Human herpesvirus 1) activities were tested (CC50 and IC50 determinations) during screening studies.

## Graphical abstract

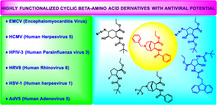

**Abbreviations**
CC_50_
: 50% cytotoxic concentration
CPE: cytopathic effect
DMF: dimethylformamide
FBS: fetal bovine serum
IC_50_
: 50% inhibitory concentration
MEM: minimum essential medium
MOI: multiplicity of infection
PFU: plaque forming units
PyFluor: 2‐pyridinesulfonyl fluoride
XtalFluor‐E: *N,N*‐diethyl‐*S,S‐*difluorosulfiliminium tetrafluoroborate

## 1 Introduction

**FIGURE 1:**
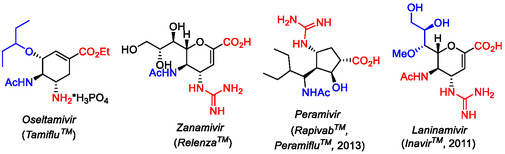
Highly functionalized cyclic amino acid derivatives as active ingredients of some antiviral marketed drugs.

Highly functionalized cyclic amino acid derivatives with multiple stereogenic centers are considered to be molecular entities of major relevance in medicinal chemistry and drug development. Some highly functionalized scaffolds of this class of compounds are active ingredients of commercially available antiviral drugs, such as *Oseltamivir* (*Tamiflu*), *Zanamivir* (*Relenza*), *Peramivir* (*Peramiflu*), or *Laninamivir* (*Inavir*) with antiviral effects (Figure 1) [1, 2, 3, 4, 5, 6, 7, 8, 9, 10 and references cited therein].

Due to the pandemic threat of various viral infections (e.g., SARS‐CoV‐2 Covid‐19 pandemic or influenza viral infections), there is a continuous and urgent need to develop strategies for the treatment of these infections, especially by designing, synthesizing, and testing effective three‐dimensional small molecular entities as potential active ingredients of pharmaceuticals [6, 11, 12, 13].

**FIGURE 2:**
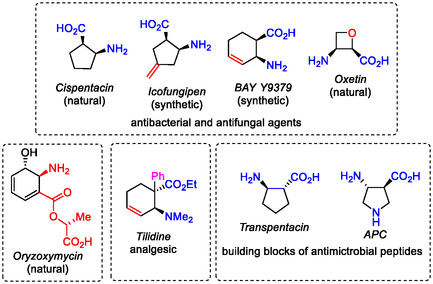
Selected bioactive cyclic amino acid derivatives.

Since the discovery of natural *Cispentacin* with antifungal effects by Japanese researchers in 1990 [14, 15, 16], the chemistry of cyclic beta(β)‐amino acids and their derivatives have developed significantly over the past 30 years. Cyclic β‐amino acids as small molecules possess a wide range of biological and pharmaceutical potentials. Some representatives are known as antifungal or antiviral agents and antibiotics or they are elements in various pharmacologically important bioactive derivatives (anticancer agents, antineuralgics, cardioprotective, or anti‐inflammatory agents). Thus, some products of this family of compounds of natural origin, such as small molecules *Oxetin* and *Oryzoxymycin*, are compounds with antibacterial effects. *Icofungipen* and *BAY Y9379* are synthetic substances with antifungal effects, and phenyl‐substituted *Tilidin* is a marketed analgesic drug (Figure 2) [14, 15, 16, 17, 18, 19, 20, 21, 22, 23, 24 and references cited therein].

Furthermore, conformationally restricted or noncanonical amino acid derivatives possess either an aryl moiety or a fluorine‐containing element in their structure. Because of their influence on the secondary structures of peptides, they might be considered as highly interesting building blocks in foldamer chemistry. For example, *Transpentacin* possessing a cyclopentene skeleton and *APC*, an amino acid derivative with a pyrrolidine skeleton, are known as interesting building blocks of novel antimicrobial peptides (Figure 2) [25, 26, 27, 28, 29, 30, 31, 32, 33 and references cited therein]. In addition, β‐amino acids function as precursors of β‐lactams and they can also be used as chiral building blocks in asymmetric syntheses [14].

**FIGURE 3:**
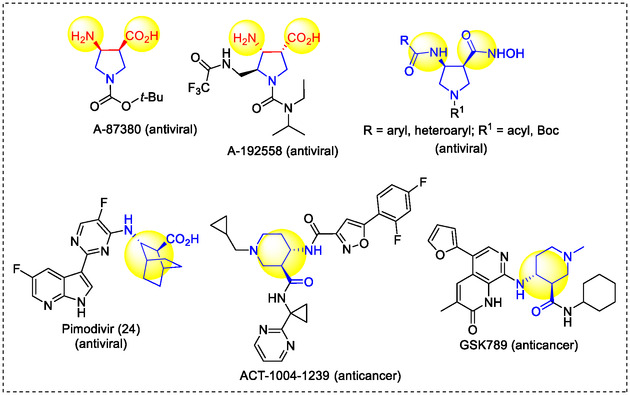
Some bioactive compounds with a cyclic β‐amino acid element in their structure.

Azaheterocyclic β‐amino acids containing an extra nitrogen atom in their skeleton possess a wide range of biological and pharmaceutical properties. Some of these classes of derivatives are known as antiviral agents and they are present in various pharmacologically important bioactive compounds (anticancer agents, antineuralgics, cardioprotective, or anti‐inflammatory agents). (Figure 3) [14, 17, 18, 19, 20, 34, 35, 36, 37, 38].

## 2 Results and Discussion

Taking into consideration the high antiviral potential, which relies on the highly functionalized cyclic amino acid derivatives, our goal was to perform the antiviral screening of various selected densely functionalized cyclic amino esters with multiple stereogenic centers as promising three‐dimensional molecular entities.

The first group of model compounds includes some highly functionalized, racemic cycloalkane derivatives, namely an oxirane‐fused cyclopentane β‐amino ester (**1**), stereo‐ or regioisomers of four azido β‐amino esters with a cyclopentane (2 and 3) or cyclohexane (**4** and **5**) framework, two stereo‐ and regioisomers of orthogonally protected diamino esters (**6** and **7**), and two triazole‐substituted β‐amino esters (**8** and **9**) (Table 1) [39, 40, 41, 42].

**TABLE 1:** Some highly functionalized cycloalkane derivatives: an oxirane‐fused cyclic β‐amino ester, azido β‐amino esters, orthogonally protected diamino esters, and triazole‐substituted β‐amino esters.

Entry	Structure	Entry	Structure	Entry	Structure
**1**	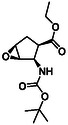 1 ref. [39]	4	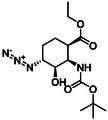 4 ref. [40]	7	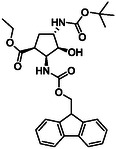 7 ref. [41]
**2**	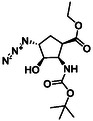 2 ref. [39]	5	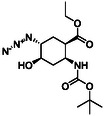 5 ref. [40]	8	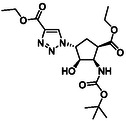 8 ref. [42]
**3**	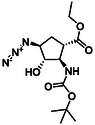 3 ref. [39]	6	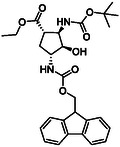 6 ref. [39]	9	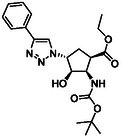 9 ref. [41]

The second type of selected model derivatives were some racemic azaheterocyclic β‐amino esters. These include two *N‐*bridged scaffolds with an azepane ring (**14** and **15**) and a compound with a piperidine skeleton (**13**). Three compounds were vicinal dihydroxylated molecules (**10**, **11** and **12**), precursors of azaheterocyclic β‐amino esters (Table 2) [43, 44, 45, 46].

**TABLE 2:** Selected azaheterocyclic β‐amino esters and some precursors.

Entry	Structure	Entry	Structure	Entry	Structure
**1**	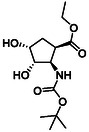 10 ref. [43]	3	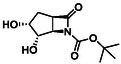 12 ref. [44]	5	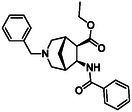 14 ref. [45]
**2**	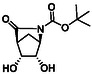 11 ref. [46]	4	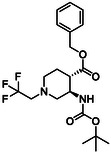 13 ref. [45]	6	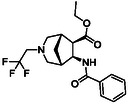 15 ref. [45]

Organofluorine chemistry is considered to be an important field of medicinal chemistry and drug research, since yearly around 25%–30% of the newly introduced small‐molecular‐based drugs approved by the FDA contain a fluorine‐containing active ingredient. Incorporation of a fluorine atom onto the structure of an organic molecule exerts a profound effect on its acid–base character, lipophilicity, polar hydrophobicity, and metabolic stability, thus increasing the bioavailability of a certain fluorine‐containing molecule with pharmaceutical potential [47, 48, 49, 50, 51, 52, 53, 54, 55, 56, 57, 58].

Fluorine‐containing molecular entities in life sciences and drug design are of high importance. As the third group of investigated compounds, we selected racemates of five fluorine‐containing five‐ or six‐membered cyclic β‐amino esters (**16**, **17**, **18**, **19,** and **20**) and a hydroxylated cyclohexene β‐amino ester (**21**) as a precursor of a related fluorinated scaffold (Table 3) [47, 48, 49, 50, 51].

**TABLE 3:** Some fluorine‐containing and hydroxylated cyclic β‐amino esters.

Entry	Structure	Entry	Structure	Entry	Structure
**1**	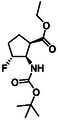 16 ref. [49]	3	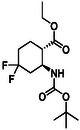 18 ref. [48]	5	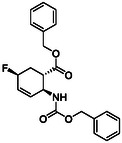 20 ref. [51]
**2**	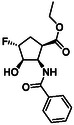 17 ref. [50]	4	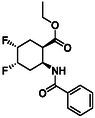 19 ref. [50]	6	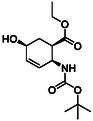 21 ref. [47]

### 2.1 Chemistry

Compounds listed in Tables 1–3 were described earlier by our group [39, 40, 41, 42, 43, 44, 45, 46, 47, 48, 49, 50, 51]. As a result of numerous experimental investigations and acquired experiences related to the chemistry of β‐amino acids, some selected compounds presented in Tables 1–3 could be prepared according to modified, improved synthetic methods (these are marked with blue letters).

**SCHEME 1:**
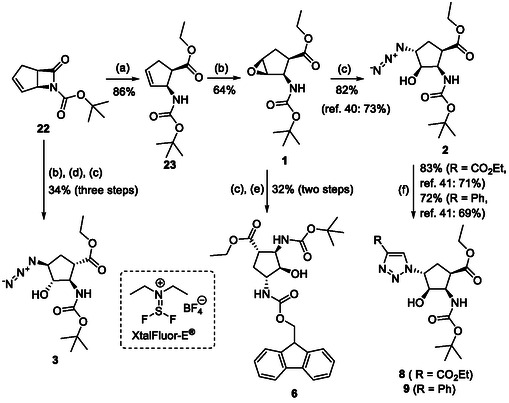
(a) EtONa, EtOH, 0°C, 1 h; (b) MCPBA, CH_2_Cl_2_, RT, 6 h; (c) NaN_3_, EtOH, XtalFluor‐E (5 mol%), Δ, 6 h; (d) EtONa, EtOH, 0°C to RT, 24 h; (e) PPh_3_, THF, H_2_O, RT, 5 h; FmocOSu, TEA, RT, 12 h; and (f) ethyl propiolate (R = CO_2_Et) or phenylacetylene (R = Ph), CuI (1 equiv), EtOH, Δ, 10 h; (Xtal‐Fluor‐E = (diethylamino)difluorosulfonium tetrafluoroborate).

Thus, the azido‐substituted cyclic β‐amino esters and the orthogonally protected diamino esters (Table 1) were prepared by starting from an unsaturated *N*‐Boc‐protected bicyclic β‐lactam **22** (Scheme 1). Lactam ring opening in **22** with EtONa, followed by epoxidation of the formed cyclopentene β‐amino ester (**23**), afforded the corresponding oxirane‐fused cyclopentane amino ester **1**. Based on our former experiences in the field of fluorinations across deoxofluorinations, the azidolysis step, through oxirane ring opening was performed under modified experimental conditions, by using NaN_3_ in EtOH under reflux condition in the presence of a catalytic amount of XtalFluor‐E (5 mol%). In this final step, formed HF, which most probably facilitates the epoxide opening, gave azido‐substituted β‐amino ester **2** in 82% isolated yield (note that according to the earlier reported method [39] the obtained yield was slightly lower, 73%). Another azido‐substituted amino ester **3** (a stereoisomer of **2**) was synthesized by epoxidation of the bicyclic lactam, followed by lactam ring opening with ethanolysis and then azidolysis with NaN_3_ in the presence of XtalFluor‐E (5 mol%) gave the desired product (Scheme 1). Next, azide derivative **2** was subjected to 1,3‐dipolar cycloaddition with alkynes to form molecules containing the 1,2,3‐triazole ring. Reactions with either ethyl propiolate or phenylacetylene were executed in the presence of CuI yielding the corresponding triazole derivatives **8** and **9**. It should be noted that the transformation with ethyl propiolate in the presence of CuI proceeded with an 83% yield. In an earlier study, this reaction was carried out under thermal conditions furnishing **8** in 71% yield [42].

**SCHEME 2:**
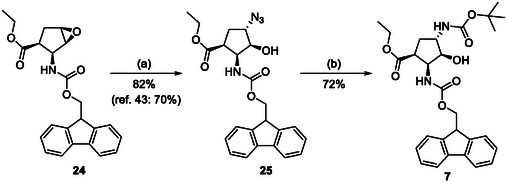
(a) NaN_3_, EtOH, XtalFluor‐E (5 mol%), Δ, 6 h and (b) H_2_, Pd/C, Boc_2_O, EtOAc, RT, 14 h.

The orthogonally (Fmoc/Boc) protected diaminocyclopentane carboxylates **7** and **6** were accessed from either the *N*‐Boc‐ or the Fmoc‐protected (**1** and **24**) epoxy amino esters through azidolysis, azide reduction, and amino group protection (Schemes 1 and 2).

**SCHEME 3:**
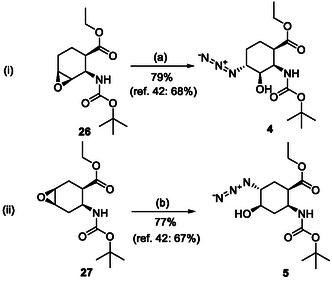
(a) NaN_3_, EtOH, XtalFluor‐E (5% mol%), Δ, 24 h and (b) NaN_3_, EtOH, XtalFluor‐E (5% mol%), Δ, 6 h.

The six‐membered azido‐substituted amino esters **4** and **5** were prepared from the corresponding epoxy amino ester regioisomers (**26** and **27**) applying our new oxirane opening methodology with NaN_3_ in the presence of XtalFluor‐E in EtOH giving the target compounds with slightly higher yields (79% and 77%) than those described earlier in the literature (68% and 67%) (Scheme 3). Note, that formation of compound **4** required prolonged heating.

**SCHEME 4:**
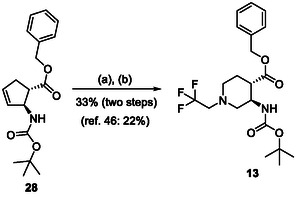
(a) O_3_, MeOH, –78°C, 0.5–1 h, then Me_2_S, –78°C to RT, 1 h and (b) 2 equiv CF_3_CH_2_NH_2_HCl, 2 equiv NaHCO_3_, 2 equiv NaBH_3_CN, 2 drops of AcOH, MeOH, –20°C to RT, 16 h.

**SCHEME 5:**
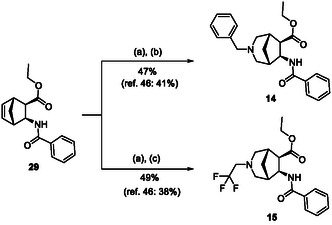
(a) O_3_, MeOH, –78°C, 0.5–1 h, then Me_2_S, –78°C to RT, 1 h; (b) 2 equiv BnNH_2_, 2 equiv NaBH_3_CN, 2 drops of AcOH, MeOH, –20°C to RT, 16 h; and (c) 2 equiv CF_3_CH_2_NH_2_HCl, 2 equiv NaHCO_3_, 2 equiv NaBH_3_CN, 2 drops of AcOH, MeOH, –20°C to RT, 16 h.

The azaheterocyclic β‐amino ester derivatives (Table 2) were synthesized utilizing a slightly modified literature method. Racemic β‐amino esters containing a cyclopentene or norbornene framework (**28** and **29**) were subjected to ozonolysis followed by treatment with the corresponding primary amine in the presence of 2 equiv of NaBH_3_CN, from –20°C to RT (Schemes 4 and 5) providing the corresponding target compounds **13**, **14**, and **15** with improved yields. Note that according to the method reported earlier the reaction was carried out at RT with 1 equiv of NaBH_3_CN.

**SCHEME 6:**
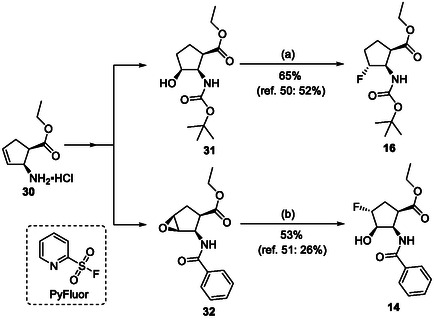
(a) XtalFluor‐E (1.5 equiv), CH_2_Cl_2_, two drops of EtOH, RT, 6 h and (b) PyFluor (1.5 equiv), CH_2_Cl_2_, two drops of EtOH, RT, 14 h (PyFluor = 2‐Pyridinesulfonyl fluoride).

The fluorine‐containing cyclic β‐amino esters (Table 3) were prepared according to a modified synthetic protocol. Fluorine‐containing amino esters with a cyclopentane ring system were prepared by starting from the hydrochloride of cyclopentene amino ester (**30**). Thus, thehydroxylated β‐amino ester (**31**) was subjected to deoxofluorination in the presence of XtalFluor‐E in the presence of a catalytic amount of EtOH furnishing fluorinated amino ester **16** in 65% yield (Scheme 6) (note that with Deoxofluor this compound was formed with a slightly inferior yield of 52% [49]). Although oxirane ring opening earlier has been accomplished with both Deoxofluor and XtalFluor‐E according to an earlier protocol [50], the same oxirane opening with PyFluor afforded the desired fluorine‐containing product **17** in a significantly higher yield (53%, Scheme 6).

**SCHEME 7:**
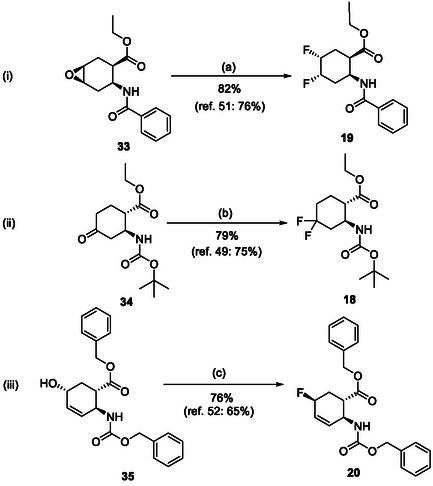
(a) PyF (1.5 equiv), CH_2_Cl_2_, RT, 8 h; (b) XtalFluor‐E (1.5 equiv), CH_2_Cl_2_, two drops of EtOH, Δ, 4 h; and (c) XtalFluor‐E (1.5 equiv), CH_2_Cl_2_, RT, 4 h.

For the synthesis of six‐membered, fluorine‐containing β‐amino ester derivatives, modified synthetic protocols were also developed. Similar to the results discussed above, PyFluor proved to be superior during oxirane opening in compound **33**, compared to the fluorinating agents applied earlier, delivering **19** in a higher yield (Scheme 7i). Geminal difluorinated amino ester **18** was accessed from the corresponding oxo compound (**34**) with XtalFluor‐E in CH_2_Cl_2_, in the presence of 2 drops of EtOH (Scheme 7ii). In contrast, the fluorinated β‐amino ester with a cyclohexene ring was obtained from **35** by hydroxy–fluorine exchange with XtalFluor‐E in CH_2_Cl_2_ (Scheme 7iii).

### 2.2 Antiviral Activity Studies

The aim of this study was to test the antiviral activity of the above‐described 21 compounds (Tables 1, 2, 3) on a range of both human and animal virus panels: EMCV (Encephalomyocarditis Virus), HCMV (Human Herpesvirus 5), HPIV‐3 (Human Parainfluenza virus 3), HRV8 (Human Rhinovirus 8), AdV5 (Human Adenovirus 5), and HSV‐1 (Human Herpesvirus 1). Simultaneously, cytotoxicity assays were conducted on cell lines corresponding to the respective viral strains (cell lines used for the propagation and replication of the viruses). The cell lines used in this study included the A549 (Human lung cancer cell line), MRC‐5 (Human lung normal fibroblasts), LLCMK2 (Macaca mulatta normal kidney cells), HeLa (Human cervix adenocarcinoma cells), and Vero (Cercopithecus aethiops normal kidney cells).

#### 2.2.1 In Vitro Cytotoxicity Screening Studies

The initial step of our studies was to test the cytotoxic properties of the investigated compounds using a screening assay at 10 µM on cell lines: A549, MRC‐5, LLCMK2, HeLa, and Vero. Compounds demonstrating cell viability of ≥50% (in both cytotoxicity and antiviral activity studies described below) were selected for further, extended studies resulting in CC_50_ (50% cytotoxic concentration, the parameter used for cell toxicity results) and IC_50_ (50% inhibitory concentration, the parameter used for antiviral activity results). The results of the in vitro cytotoxicity screening are presented in Table 4a (see Supporting Information).

#### 2.2.2 In Vitro Antiviral Screening Studies

The antiviral properties of the investigated compounds were initially assessed during a screening assay at 10 µM on the viruses panel: EMCV, HCMV, HPIV‐3, HRV8, AdV5, and HSV‐1. Compounds demonstrating cell viability of ≥50% (in both aforementioned studies of cell toxicity and antiviral activity) as well as compounds with a percentage of plaques <50% of viral HCMV control and MRC‐5 cells viability >50% were selected for further studies, including CC_50_ and IC_50_ evaluation. The results of the in vitro screening, including the assessment of cellular toxicity and the determination of antiviral activity, are presented in Table 4a (see Supporting Information). On the basis of the results, six compounds have been selected for CC_50_ and IC_50_ evaluation. These include compound **2** (anti‐HRV8 studies and corresponding HeLa cells), compound **5** (anti‐HSV1 studies and corresponding Vero cells), and compounds **7**, **16**, **14**, and **15** (anti‐HCMV studies and corresponding MRC‐5 cells).

#### 2.2.3 Cytotoxic Activity Assay in the Range of 0.1–1000 µM

Screening studies described above revealed that the most promising results were observed for compounds **2**, **5**, **7**, **14**, **15**, and **16**. Note, that these compounds as highly functionalized cyclic compounds possessing those types of essential groups (e.g., azido group, a second protonable N‐atom, or orthogonally protected amino functions) which are also present in the structure of antiviral drugs such as Tamiflu, Peramivir or azidothymidine (AZT) [14, 24]. Obviously, for more successful screening and prediction of the activity potential molecular entities further investigations based on structure‐activity studies are needed.

Selected compounds were investigated for cytotoxic activity against corresponding host cell lines included compounds **2** (HeLa cells), **5** (Vero cells) as well as compounds **7**, **14**, **15**, and **16** (MRC‐5 cells). Cytotoxicity was determined by MTT assay and expressed as CC_50_ parameter (50% cytotoxic concentration). All results are collected in Table 4.

**TABLE 4:** CC_50_ and IC_50_ results.

**CC**_**50**_ **and IC**_**50**_ **values**								
No.	Compound	**HeLa CC**_**50**_, **uM**	**HRV8 IC**_**50**_, **uM**	**Vero CC**_**50**_, **uM**	**HSV‐1 IC**_**50**_, **uM**	**MRC5 CC**_**50**_, **uM**	**HCMV IC**_**50**_, **uM**	SI
1	**2**	342.50 ± 6010	35.67 ± 7.50	ND	ND	ND	ND	**9.60**
2	**7**	ND	ND	ND	ND	270.67 ± 29.28	4.00 ± 3.61	**67.67**
3	**5**	ND	ND	758.75 ± 1.77	>758.75	ND	ND	—
4	**16**	ND	ND	ND	ND	20.00 ± 5.00	14.5 ± 0.70	**1.38**
5	**14**	ND	ND	ND	ND	266.67 ± 41.93	4.3 ± 0.93	**62.02**
6	**15**	ND	ND	ND	ND	555.00 ± 7.07	92.00 ± 4.00	**6.03**
7	**GCV**	ND	ND	ND	ND	>1000	3.07 ± 0.63	**325.73**
8	**ACV**	ND	ND	735.00 ± 14.14	5.1 ± 0.79	ND	ND	**144.12**

^a^Selectivity index (SI) = CC_50_/IC_50_ for the equivalent cell lines.

^b^ND, not determined.

#### 2.2.4 Antiviral Activity Assay in the Range of 0.1–1000 µM

The compounds selected during the screening process have been evaluated for their antiviral activity against the following viruses: HRV8 (**2**), HSV‐1 (**5**), and HCMV (**7**, **14**, **15**, **16**).

Antiviral activity results are shown as an IC_50_ parameter (50% inhibitory concentration) and results collected in Table 4. Acyclovir (ACV) and ganciclovir (GCV) were used as positive controls in the anti‐HSV‐1 and anti‐HCMV assays. The selectivity index (SI), defined as the ratio of a compound toxic concentration (CC_50_) against its inhibitory concentration (IC_50_), was calculated for compounds with antiviral properties.

Considering all the results acquired, it can be concluded that the most beneficial results were obtained in terms of anti‐HCMV activity. Compound **7** exhibited selective anti‐HCMV activity (IC_50_ = 4.00 ± 3.61 µM) with low cytotoxicity in MRC‐5 cells (CC_50_ = 270.67 ± 29.28 µM), yielding a high selectivity index (SI; 67.67). Compound **16** inhibited HCMV replication (IC_50_ = 14.5 ± 0.70 µM) but showed high cytotoxicity (CC_50_ = 20.00 ± 5.00 µM), resulting in a low SI (1.38). Compounds **14** and **15** demonstrated anti‐HCMV activity with different selectivity profiles. Compound **14** showed potent and selective inhibition (IC_50_ = 4.3 ± 0.93 µM; SI = 62.02), whereas **15** exhibited weaker activity (IC_50_ = 92.00 ± 4.00 µM) and low selectivity (SI = 6.03). Overall, compounds **7** and **14** emerged as the most promising selective anti‐HCMV candidates. However, it should be noted that the results obtained were approximately five times lower than those observed for the reference drug ganciclovir.

Compound **2** showed low cytotoxicity in HeLa cells (CC_50_ = 342.50 ± 60.10 µM) and low antiviral activity against HRV8 (IC_50_ = 35.67 ± 7.50 µM), resulting in a SI < 10.

Detailed studies did not confirm any antiviral properties of compound **5** against the HSV‐1 virus within the nontoxic range of concentrations.

## 3 Conclusions and Outlook

In the current article, we have described the synthesis of some selected multifunctionalized β‐amino acid derivatives with antiviral potential.

The model compounds included azido‐functionalized β‐amino esters, orthogonally protected diamino esters, an oxirane‐fused cyclic β‐amino ester, 1,2,3‐triazole‐substituted β‐amino esters, mono‐ and difluorinated cyclic β‐amino esters, mono and dihydroxylated cyclic β‐amino esters, and β‐amino esters with piperidine or azepane framework. These compounds have been studied to test their anti‐HCMV, anti‐HRV8 and anti‐HSV‐1 activity (CC_50_ and IC_50_ determinations) during screening studies. Based on these preliminary antiviral investigations we aim to plan the synthesis and test of other highly functionalized novel, conformationally restricted non‐natural amino acid derivatives as three‐dimensional molecular entities with multiple chiral centers. For further successful screening and prediction of the activity potential molecular entities further investigations based on structure‐activity studies are needed. The structure‐activity studies for the rational design of potential molecules are part of a forthcoming project in collaboration with a cooperating partner.

## 4 Experimental Part

### 4.1 Chemistry

#### 4.1.1 Azidolysis of Epoxy Amino Esters

To a solution of epoxy amino ester (2.5 mmol) in EtOH (15 mL), NaN_3_ (1.5 equiv), and XtalFluor‐E (5 mol%) were added and the mixture was stirred at reflux temperature for the time indicated on Schemes 1, 2, and 3 (the reaction was monitored by TLC). Then the solvent was evaporated under reduced pressure. The crude material was taken up in EtOAc (20 mL) and washed with H_2_O (2 × 10 mL). The combined organic layers were dried (Na_2_SO_4_) and concentrated under reduced pressure. The crude material was purified by means of column chromatography on silica gel (hexane/AcOEt 4:1).

#### 4.1.2 Formation of 1,2,3‐Triazole‐Containing β‐Amino Esters

To a solution of azido amino ester (0.8 mmol) in EtOH (10 mL), the acetylenic compound (1 equiv) and CuI (1 equiv) were added, and the solution was stirred at reflux temperature for 10 h (see Scheme 1). The solid was filtered off, the filtrate was concentrated under reduced pressure, and the crude material was purified by means of column chromatography on silica gel (hexane/EtOAc 4:1).

#### 4.1.3 Synthesis of Azaheterocyclic β‐Amino Esters

The cycloalkene amino esters (400 mg) and 40 mL of MeOH were mixed in a three‐necked round‐bottom flask. The reaction mixture was cooled with a dry ice/acetone bath (–78°C). With the help of an ozone generator, ozone was flowed into the reaction mixture. The reaction mixture was stirred until completion of the reaction (monitoring by TLC) (see Schemes 4 and 5). Then, after the cooling bath was removed, 0.4 ml of dimethyl sulfide was added to the mixture. This mixture was stirred while warming to room temperature for 1 h. Next, the mixture was cooled again to –20°C (salted ice bath) and the amine hydrochloride (2 equiv), NaHCO_3_ (2 equiv), NaCNBH_3_ (2 equiv), and two drops of AcOH were added to the reaction mixture followed by stirring for 16 h. Then, the solvent was evaporated, and the crude material was diluted with EtOAc (2 ml), and washed with 3 × 10 mL of water. The organic layer was dried over Na_2_SO_4_ and then concentrated under reduced pressure. The products were purified by means of column chromatography (hexane/EtOAc 3:1).

#### 4.1.4 Synthesis of Fluorine‐Containing β‐Amino Esters

##### 4.1.4.1 Method A

To a solution of hydroxylated amino ester derivative (1 mmol) in CH_2_Cl_2_ (10 mL) under an Ar atmosphere, XtalFluor‐E (1.5 equiv) was added at 0°C. The mixture was stirred at room temperature for the time indicated (see Schemes 6 and 7), then diluted with CH_2_Cl_2_ (25 mL) and washed with brine (3 × 10 mL). The organic layer was dried over Na_2_SO_4_ and concentrated under reduced pressure. The crude material was purified by means of column chromatography (silica gel, hexane/EtOAc 5:1).

##### 4.1.4.2 Method B

To a solution of oxo amino ester derivative (1 mmol) in CH_2_Cl_2_ (10 mL) under an Ar atmosphere, XtalFluor‐E (1.5 equiv) was added at 0°C. The mixture was stirred at reflux temperature for 4 h, then diluted with CH_2_Cl_2_ (25 mL) and washed with brine (3 × 10 mL). The organic layer was dried over Na_2_SO_4_ and concentrated under reduced pressure. The crude material was purified by means of column chromatography (silica gel, hexane/EtOAc 5:1) (see Scheme 7).

##### 4.1.4.3 Method C

To a solution of epoxy amino ester (1 mmol) in CH_2_Cl_2_ (8 mL), PyFluor (1.5 equiv) was added, and the mixture was stirred at room temperature in the presence or without EtOH (see Schemes 6 and 7) for the time indicated. Then the mixture was diluted with CH_2_Cl_2_ (20 mL) and washed with brine (3 × 10 mL). The organic layer was dried over Na_2_SO_4_ and concentrated under reduced pressure. The crude product was purified by means of column chromatography (silica gel, hexane/EtOAc 3:1) (see Schemes 6 and 7).

The NMR and HRMS spectra, melting points, and retention factors (TLC) of the synthetized substances were identical to those described earlier in the literature (see also Supporting Information).

### 4.2 Antiviral Activity Studies

#### 4.2.1 In Vitro Cytotoxicity Screening Studies on the Corresponding Cell Lines

Cytotoxic properties of compounds were assessed on A549, MRC‐5, LLCMK2, HeLa, and Vero cell lines. Cell lines were purchased from the American Type Culture Collection (ATCC, Manassas, VA, USA). All tested compounds were dissolved in DMSO (dimethyl sulfoxide, Sigma‐Aldrich, Darmstadt, Germany) and then suspended in minimum essential medium (MEM; Sigma‐Aldrich, Darmstadt, Germany) supplemented with 2% heat‐inactivated fetal bovine serum (FBS; Sigma‐Aldrich, Darmstadt, Germany) and 1% penicillin/streptomycin mixture (10 000 units/mL penicillin G with 10 mg/mL streptomycin, Sigma‐Aldrich, Darmstadt, Germany). The final concentration of DMSO in the medium was 0.1%. A549, MRC‐5, LLCMK2, HeLa, and Vero cells were propagated in MEM supplemented with 10% heat‐inactivated FBS and 1% penicillin/streptomycin mixture. Upon reaching 80%–90% confluency, cells were harvested with 0.25% trypsin in 1 mM EDTA and seeded into 96‐well microplates at 2 × 10^4^ cells/well. After overnight incubation at 37°C in a humidified atmosphere containing 5% CO_2_, the culture medium was replaced with a 100 µL freshly prepared solution of tested compounds diluted with a maintenance medium supplemented with 2% FBS and antibiotics to achieve compound concentrations of 10 µM. All experiments were carried out in triplicate. Compounds treated and untreated cells (control group) were incubated at 37°C for 48 h in a humidified atmosphere containing 5% CO_2_. After incubation with drugs, the cells were treated with 3‐(4,5‐dimethylthiazol‐2‐yl)−2,5‐diphenyltetrazolium bromide dye solution (MTT, Sigma‐Aldrich, Darmstadt, Germany) (25 µL, 5 mg/mL) for 2 h and lysed with solvent solution (100 µL) containing: DMF (Sigma‐Aldrich, Darmstadt, Germany) (45 mL), SDS (Sigma‐Aldrich, Darmstadt, Germany) (13.5 g), and distilled water (55 mL). After overnight incubation at 37°C, optical density at 550 nm and a reference wavelength of 670 nm were measured on a microplate spectrophotometer, Varioskan Lux (Thermo Fisher Scientific, Waltham, MA, USA).

#### 4.2.2 In Vitro Antiviral Screening Studies

Antiviral properties of compounds were assessed against the following viruses: EMCV, HCMV, HPIV‐3, HRV8, AdV5, and HSV‐1. Viruses were purchased from the ATCC. All tested compounds were dissolved in DMSO and then suspended in MEM supplemented with 2% heat‐inactivated FBS, and 1% penicillin/streptomycin mixture. The final concentration of DMSO in the medium was 0.1%. A549, MRC‐5, LLCMK2, HeLa, and Vero cells were propagated, harvested and seeded as it was described above. After overnight incubation of cells at 37°C in a humidified atmosphere containing 5% CO_2_, the culture medium was removed, and cells were inoculated with the respective virus solution in MEM supplemented with 2% FBS and antibiotics (HSV‐1, ADV5, EMCV and HRV8 MOI 0.005; HPIV‐3 MOI 0.01; HCMV 20 PFU (plaque forming units per well). After 1‐h (HSV‐1, HPIV‐3, AdV5, HRV8, and EMCV) or 2 h adsorption period (HCMV), the residual virus was removed, and the infected cells were further incubated with a 100 µL freshly prepared solution of tested compounds diluted with a maintenance medium supplemented with 2% FBS and antibiotics to obtain compound concentrations of 10 µM. All experiments were carried out in triplicate. Cell monolayers were incubated with the compounds at 37°C (35°C for HRV8) in a humidified atmosphere containing 5% CO_2_ until the typical cytopathic effect (CPE) was visible. Viral infection was evaluated by MTT assay or plaque reduction assay (HCMV). After incubation with drugs, the cells were treated with MTT (25 µL, 5 mg/mL) for 2 h and lysed with solvent solution (100 µL) containing DMF 45 mL), SDS (13.5 g), and distilled water (55 mL). After overnight incubation, the optical density at 550 nm and a reference wavelength of 670 nm were measured on a microplate spectrophotometer, Varioskan Lux. The number of HCMV plaques was counted under an inverted microscope Olympus IX73 (Olympus, Tokyo, Japan).

#### 4.2.3 Cytotoxic Activity Assay in the Concentration Range of 0.1–1000 µM

Cytotoxic properties of selected compounds were assessed on MRC‐5, HeLa, and Vero cell lines. All tested compounds were dissolved in DMSO and then suspended in MEM supplemented with 2% heat‐inactivated FBS and 1% penicillin/streptomycin mixture. The final concentration of DMSO in the medium was 0.1%. Investigated cells were propagated and seeded as described above. After overnight incubation at 37°C in a humidified atmosphere containing 5% CO_2_, the culture medium was replaced with a 100 µL freshly prepared solution of tested compounds diluted with a maintenance medium supplemented with 2% FBS and antibiotics to obtain compound concentrations in the range of 0.1–1000 µM. All experiments were carried out in triplicate. Cells exposed to compounds studied and unexposed cells (control group) were incubated at 37°C for 48 h in a humidified atmosphere containing 5% CO_2_. After the incubation, MTT assay was used to assess the cytotoxic concentration (CC_50_), as was described above. CC_50_ was defined as the concentration required to reduce cell viability by 50% compared to untreated controls and was calculated by linear regression analysis of the dose–response curves obtained from the data.

#### 4.2.4 Antiviral Activity Assay in the Concentration Range of 0.1–1000 µM

Antiviral properties of selected compounds were assessed against viruses: HCMV, HRV8, and HSV‐1. All tested compounds were dissolved in DMSO and then suspended MEM supplemented with 2% heat‐inactivated FBS and 1% penicillin/streptomycin mixture. The final concentration of DMSO in the medium was 0.1%. Investigated cells were propagated and seeded as described above. The culture medium was removed from confluent cells grown in 96‐well microplates, and the cells were inoculated with virus solutions in MEM supplemented with 2% FBS and antibiotics (HSV‐1 and HRV8 MOI 0.005; HCMV 20 PFU/well (plaque forming units per well). After a 1 h (HSV‐1 and HRV8) or 2‐h adsorption period (HCMV), residual viral particles were removed, and infected cells were further incubated with MEM supplemented with 2% FBS and antibiotics containing compound concentrations in the range of 0.1–1000 µM [59]. All experiments were carried out in triplicate. The cell monolayers were incubated with the investigated compounds at 37°C (35°C for HRV8) in a humidified atmosphere containing 5% CO_2_ until a CPE was visible. Viral infection was evaluated by MTT assay (as described previously) or plaque reduction assay. HCMV plaques were counted under an inverted microscope, Olympus IX73 (Olympus, Tokyo, Japan). Antiviral activity was expressed as the concentration required to reduce the number of viral plaques to 50% of the control (virus‐infected but untreated).

## Supporting Information

Additional supporting information can be found online in the Supporting Information section. **Supporting** **Table 4a.** Screening assay results on a panel of cell lines and viruses (viability ‐ % of control after exposition on investigated drug^*^).

## Conflicts of Interest

The authors declare no conflicts of interest.

## Supporting information

Supplementary Material

## Data Availability

All data generated or analyzed during this study are included in this article [and its supplementary information files].
