# Supplementary material for: Functionalized Cyclic Beta‐Amino Acid Derivatives With Antiviral Potential
Source: ChemMedChem. 2026 Mar 30;21(6):e202500862. doi: 10.1002/cmdc.202500862 (PMC13035932; doi:10.1002/cmdc.202500862)
Supplement: Supplementary file 1 — Supplementary Material [file CMDC-21-e202500862-s001.pdf]

Supporting Information

Functionalized Cyclic β-Amino Acid Derivatives with Antiviral Potential

Melinda Nonn,[a],[b] Marta Denel-Bobrowska[c] Balázs Volk[d] Agnieszka B. Olejniczak,[c]* Loránd Kiss[e]*

[a]MTA TTK Lendület Artificial Transporter Research Group, Institute of Materials and Environmental Chemistry, HUN-REN Research Center for Natural Sciences, Hungarian Academy of Sciences, H-1117 Budapest, Magyar Tudósok krt. 2, Hungary
[b]National Drug Research and Development Laboratory, HUN-REN Research Centre for Natural Sciences, Magyar tudósok krt. 2, H-1117 Budapest, Hungary
[c]Institute of Medical Biology, Polish Academy of Sciences, 106 Lodowa St., 93-232 Łódź, Poland
[d]Egis Pharmaceuticals Plc., Directorate of Drug Substance Development, P.O. Box 100, H-1475 Budapest, Hungary
[e]Institute of Organic Chemistry, Stereochemistry Research Group, HUN-REN Research Centre for Natural Sciences, H-1117 Budapest, Magyar tudósok krt. 2, Hungary

E-mail: kiss.lorand@ttk.hu; kiss.lorand00@gmail.com
Tel: +36-30-1600354
Kiss L ORCID iD: 0000-0003-2346-9816

Antiviral studies

Table 4a. Screening assay results on a panel of cell lines and viruses (viability - % of control after exposition on investigated drug*).

Cell lines/Viruses												
**No.**	**Compound**	**A549**	**EMCV**	**HeLa**	**AdV5**	**HRV8**	**LLCMK2**	**HPIV-3**	**MRC5**	**HCMV#**	**Vero**	**HSV-1**
**1**	**1**	102.63	31.57	99.51	37.89	46.34	106.21	35.42	105.37	99.29	101.52	17.96
**2**	**2**	106.22	31.84	104.66	38.36	63.68	104.70	35.57	83.05	54.16	105.00	17.58
**3**	**3**	98.59	31.34	100.08	37.67	41.13	101.72	33.54	71.01	160.92	104.23	17.61
**4**	**10**	103.21	30.71	105.28	38.29	35.83	97.93	31.20	72.02	200.96	102.88	18.75
**5**	**9**	102.74	24.44	102.41	35.59	44.43	101.53	29.72	97.48	102.84	100.41	16.84
**6**	**11**	103.93	32.43	98.93	38.73	38.20	106.42	34.29	80.52	66.84	102.70	23.37
**7**	**7**	97.82	31.45	94.70	39.57	40.96	96.97	36.09	94.28	12.73	98.74	18.24
**8**	**12**	101.02	31.36	99.55	40.88	38.24	94.59	36.40	90.70	125.87	99.72	19.56
**9**	**13**	96.16	30.39	96.84	37.07	42.62	90.81	34.09	109.18	227.77	97.30	16.55
**10**	**6**	78.59	20.69	81.71	12.73	34.90	60.03	43.69	92.88	76.33	97.87	23.07
**11**	**21**	100.99	29.34	98.11	15.65	35.56	90.55	47.04	94.01	60.72	107.41	47.63
**12**	**4**	96.04	25.15	85.31	15.20	34.12	104.77	47.53	97.28	413.62	95.01	44.43
**13**	**5**	91.83	25.87	63.25	14.49	36.32	102.77	45.17	97.68	204.99	99.82	71.01
**14**	**18**	78.26	22.39	30.89	13.96	46.63	89.73	40.86	108.17	145.93	94.47	39.74
**15**	**16**	94.26	36.09	110.40	15.63	42.99	89.13	48.16	86.65	31.10	102.75	46.93
**16**	**17**	92.38	30.08	105.77	15.01	45.05	78.75	47.95	62.17	107.61	105.15	44.95
**17**	**19**	96.06	29.71	101.16	14.93	47.05	70.94	48.48	102.19	119.39	101.57	47.53
**18**	**20**	95.19	23.30	89.52	14.93	48.18	68.52	46.12	101.62	200.99	107.75	32.94
**19**	**14**	85.45	20.67	67.89	11.25	44.38	60.55	38.57	99.87	0	95.82	31.41
**20**	**15**	96.56	20.13	69.24	12.06	45.86	95.42	47.15	93.94	0	98.88	41.60
**21**	**8**	98.03	23.63	29.93	14.36	48.29	88.29	42.42	88.09	63.61	88.81	37.64

compounds with a viability > 50% for both, cytotoxicity and antiviral screening are selected for further studies including CC_{50} and IC_{50} evaluation
#compounds with a percentage of plaques < 50% of viral HCMV control and MRC-5 cells viability > 50% are selected for further studies including CC_{50} and IC_{50} evaluation

General information about chemical syntheses

Chemicals were purchased from Sigma-Aldrich and Merck. TLC plates (TLC 60 F_{254} 25 Silica gel on aluminum sheets) were purchased from Merck. Silica gel for flash chromatography (pore size 15-40 μm) was purchased from Merck. Flash chromatography purifications were obtained with CombiFlash Rf + (on normal phase) or with a CombiFlasf Rf EZ Prep (on reversed phase) using gradient eluent systems hexane/ethyl acetate, or dichloromethane/methanol, or acetonitrile/water. Melting points were determined with a PGH Rundfunk-Fernsehen Niederdorf with a Multi-Thermometer apparat. LC-MS samples were measured with an LC-MS 2020 Shimadzu instrument. NMR spectra were recorded at room temperature using Varian NMR System spectrometers operating at 500 MHz for ^{1}H frequency. Samples were dissolved in $CDCl_3$. All spectra were referenced to the residual solvent signal of $CHCl_3$ (7.26 ppm). Chemical shifts were reported in ppm, and J-couplings in Hz. Spectra were analyzed using MestreNova software. HRMS data were acquired on a Sciex 5600+ Triple TOF high resolution mass spectrometer in flow injection mode. The resolution was over 30000 in the entire mass range.

^{1}H NMR, ^{13}C NMR and HRMS spectra of the studied compounds

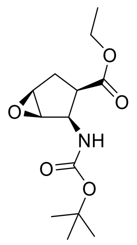

1

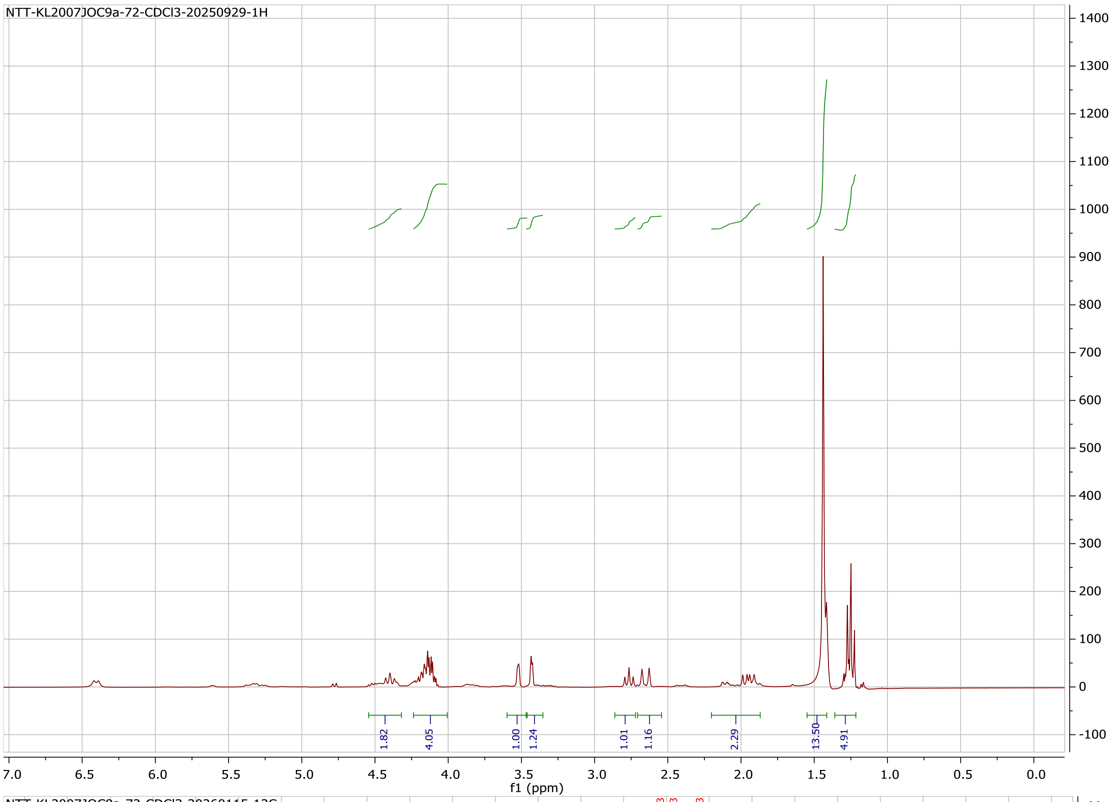

NTT-KL2007JOC9a-72-CDCl3-20250929-1H
1.82
4.05
1.00
1.24
1.01
1.16
2.29
13.50
4.91
f1 (ppm)

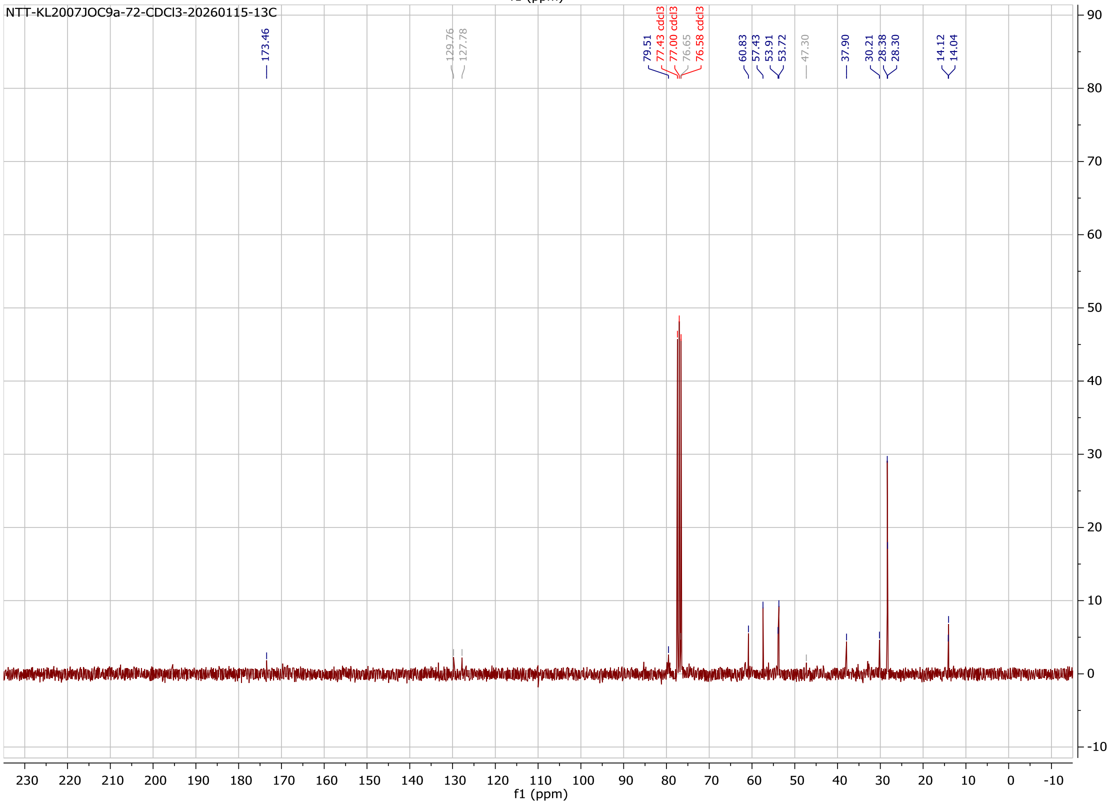

NTT-KL2007JOC9a-72-CDCl3-20260115-13C
173.46
129.76
127.78
79.51
77.43 cdcl3
77.00 cdcl3
76.65
76.58 cdcl3
60.83
57.43
53.91
53.72
47.30
37.90
30.21
28.38
28.30
14.12
14.04
f1 (ppm)

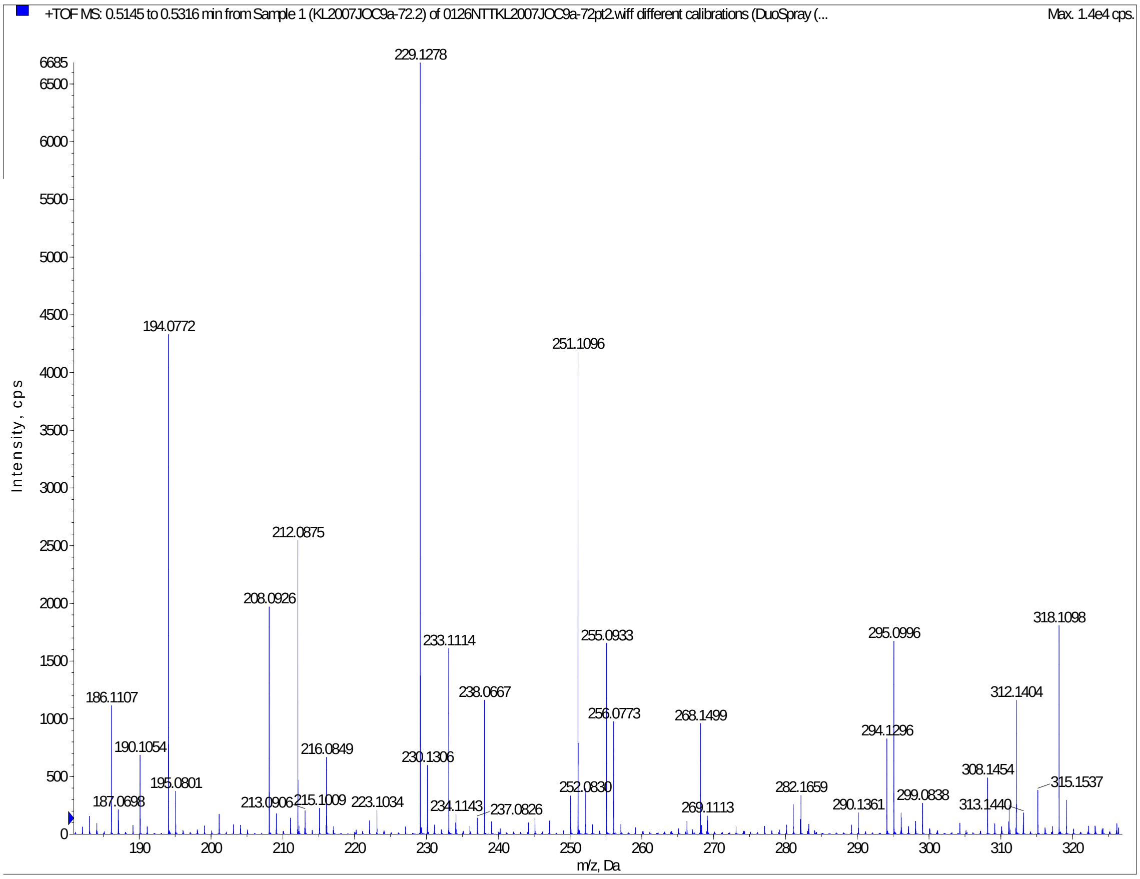
+TOF MS: 0.5145 to 0.5316 min from Sample 1 (KL2007JOC9a-72.2) of 0126NTTKL2007JOC9a-72pt2.wiff different calibrations (DuoSpray (...
Max. 1.4e4 cps.
Intensity, cps
m/z, Da
229.1278
194.0772
251.1096
212.0875
208.0926
233.1114
255.0933
295.0996
318.1098
238.0667
186.1107
312.1404
256.0773
268.1499
294.1296
190.1054
216.0849
230.1306
308.1454
195.0801
252.0830
282.1659
315.1537
187.0698
213.0906
215.1009
223.1034
234.1143
237.0826
269.1113
290.1361
299.0838
313.1440

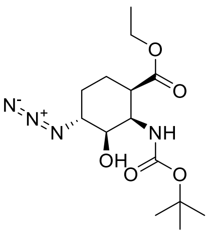

N-
N+
N
NH
OH
O
O
O
O

4

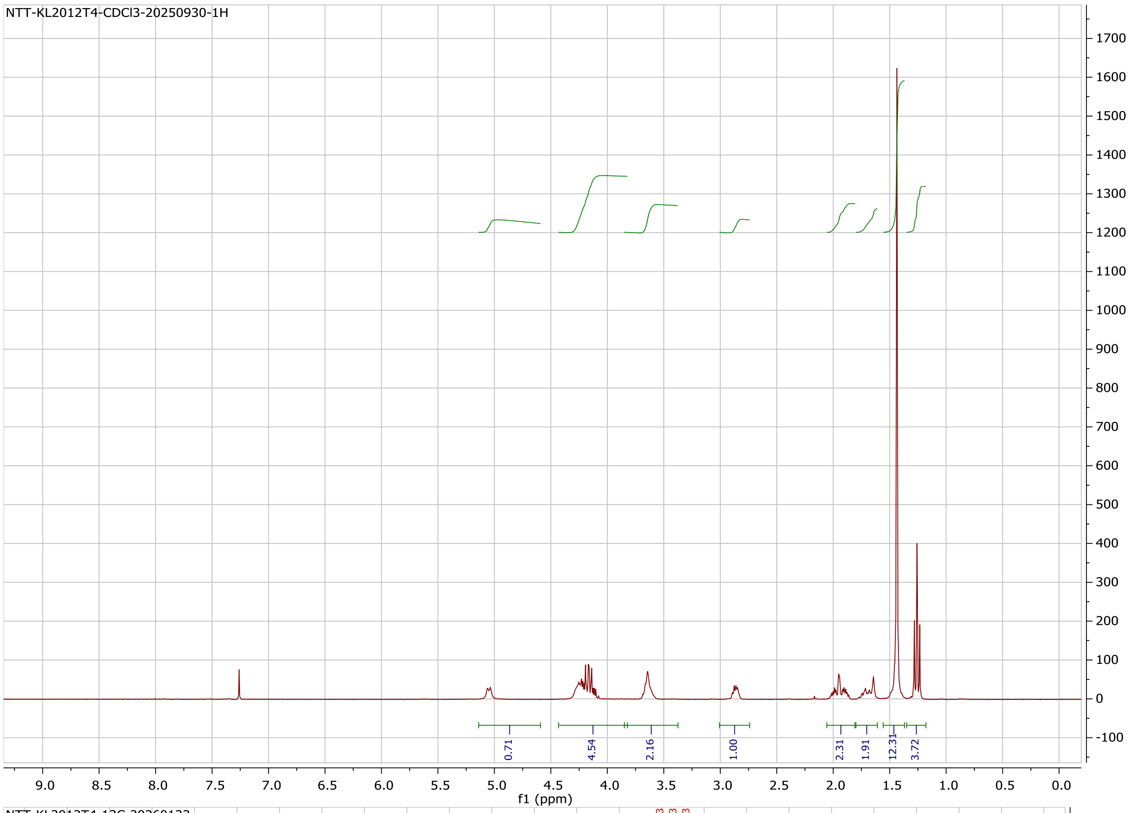

NTT-KL2012T4-CDCl3-20250930-1H
0.71
4.54
2.16
1.00
2.31
1.91
12.31
3.72
f1 (ppm)

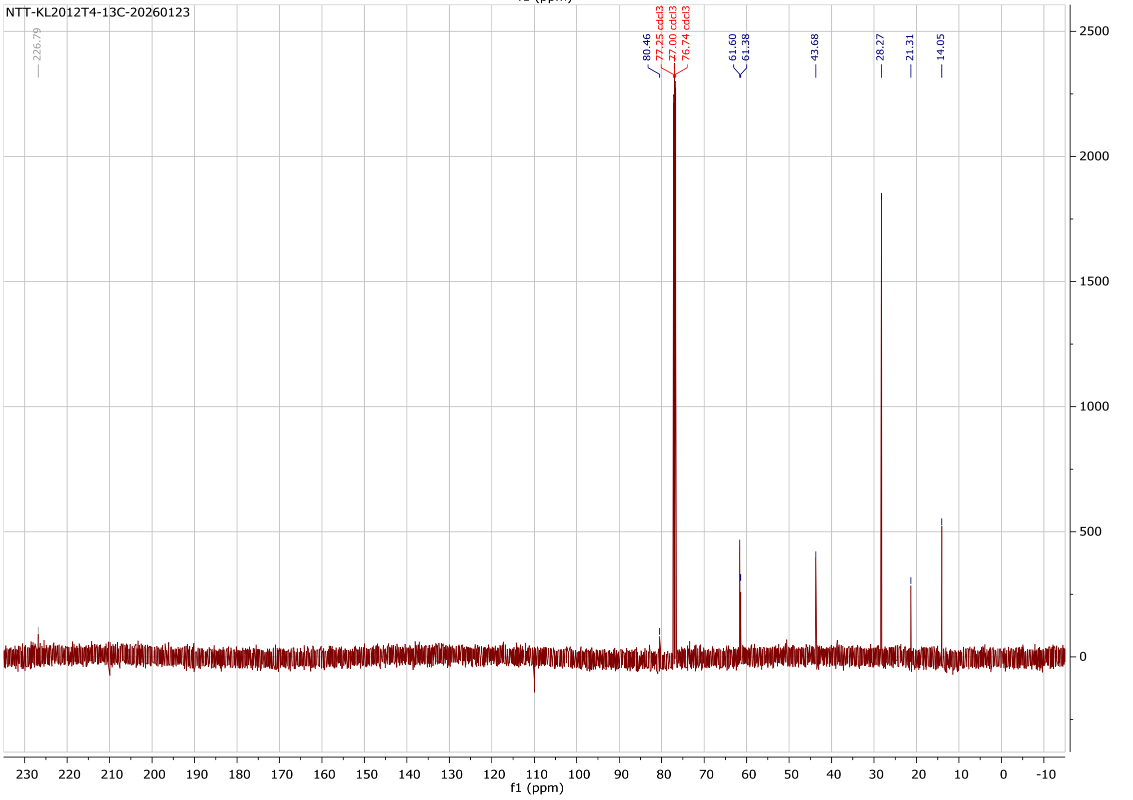

NTT-KL2012T4-13C-20260123
226.79
80.46
77.25 cdcl3
77.00 cdcl3
76.74 cdcl3
61.60
61.38
43.68
28.27
21.31
14.05
f1 (ppm)

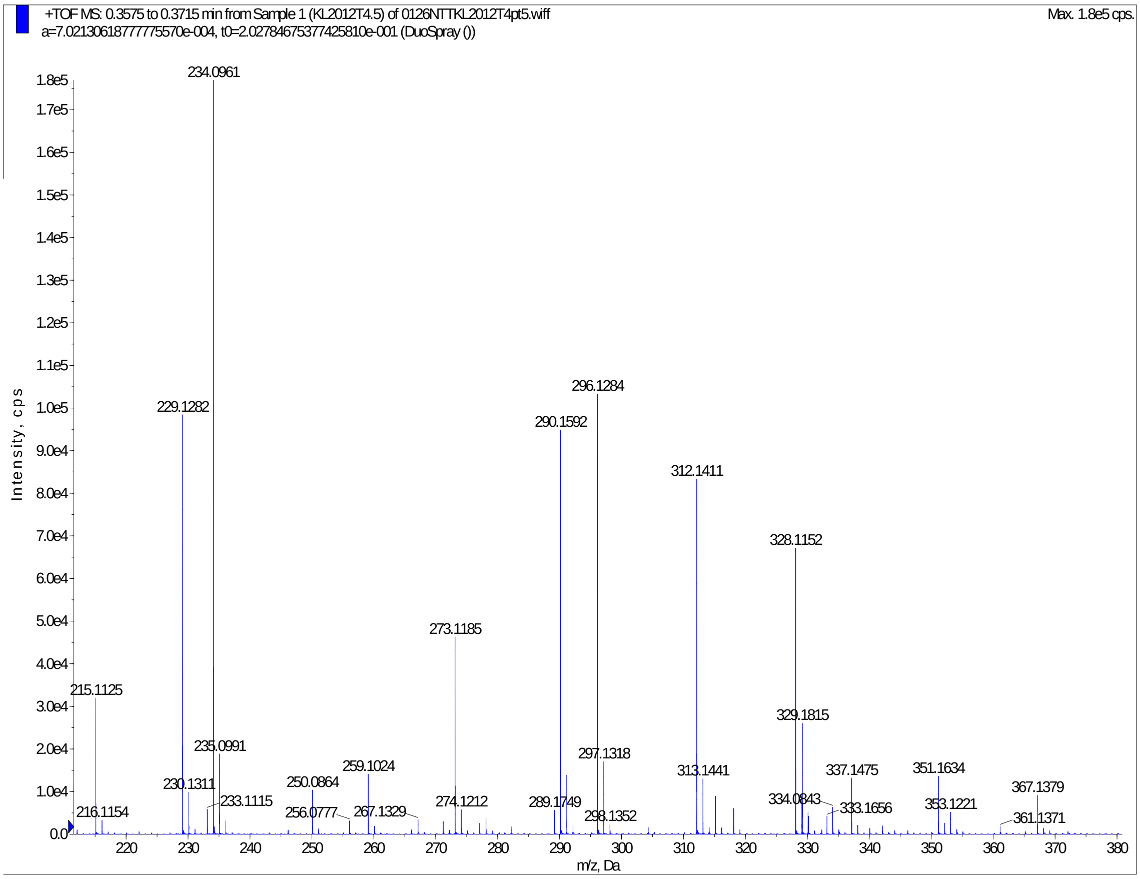

+TOF MS: 0.3575 to 0.3715 min from Sample 1 (KL2012T4.5) of 0126NTTKL2012T4pt5.wiff
a=7.02130618777775570e-004, t0=2.02784675377425810e-001 (DuoSpray ())
Max. 1.8e5 cps.
Intensity, cps
m/z, Da
234.0961
229.1282
296.1284
290.1592
312.1411
328.1152
273.1185
215.1125
329.1815
235.0991
297.1318
259.1024
313.1441
337.1475
351.1634
230.1311
250.0864
367.1379
233.1115
274.1212
289.1749
334.0843
353.1221
216.1154
256.0777
267.1329
298.1352
333.1656
361.1371

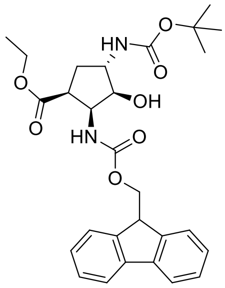

7

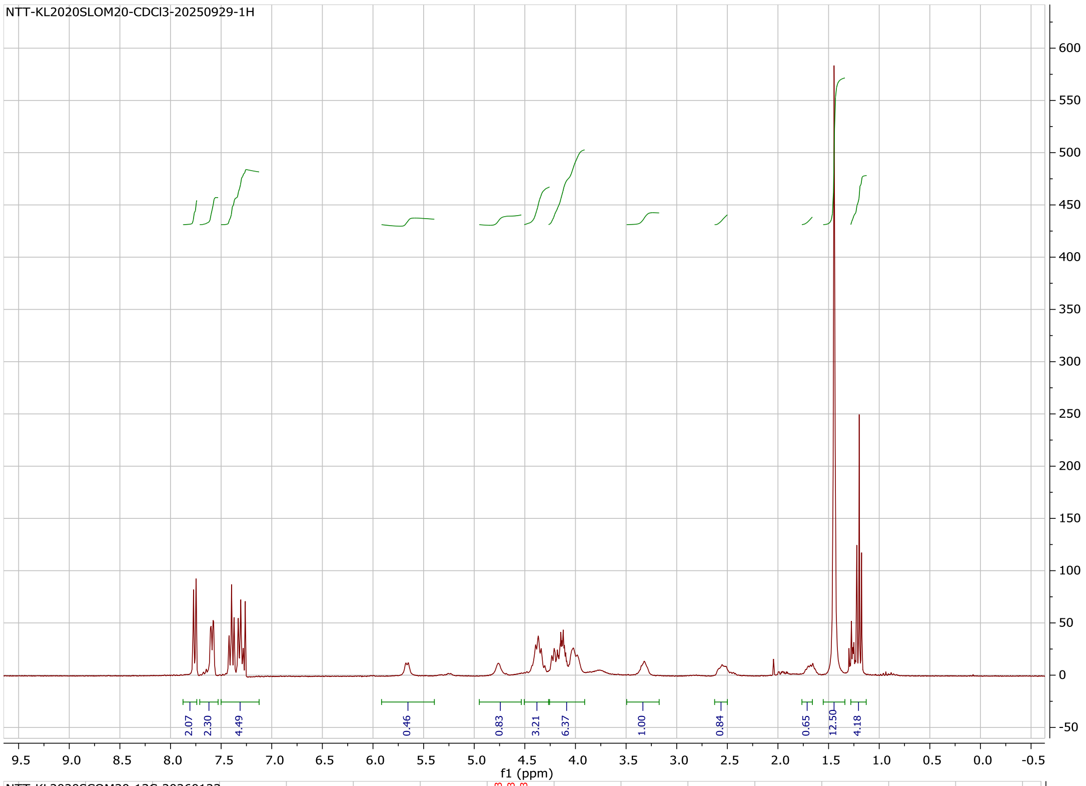

NTT-KL2020SLOM20-CDCl3-20250929-1H
2.07
2.30
4.49
0.46
0.83
3.21
6.37
1.00
0.84
0.65
12.50
4.18
f1 (ppm)

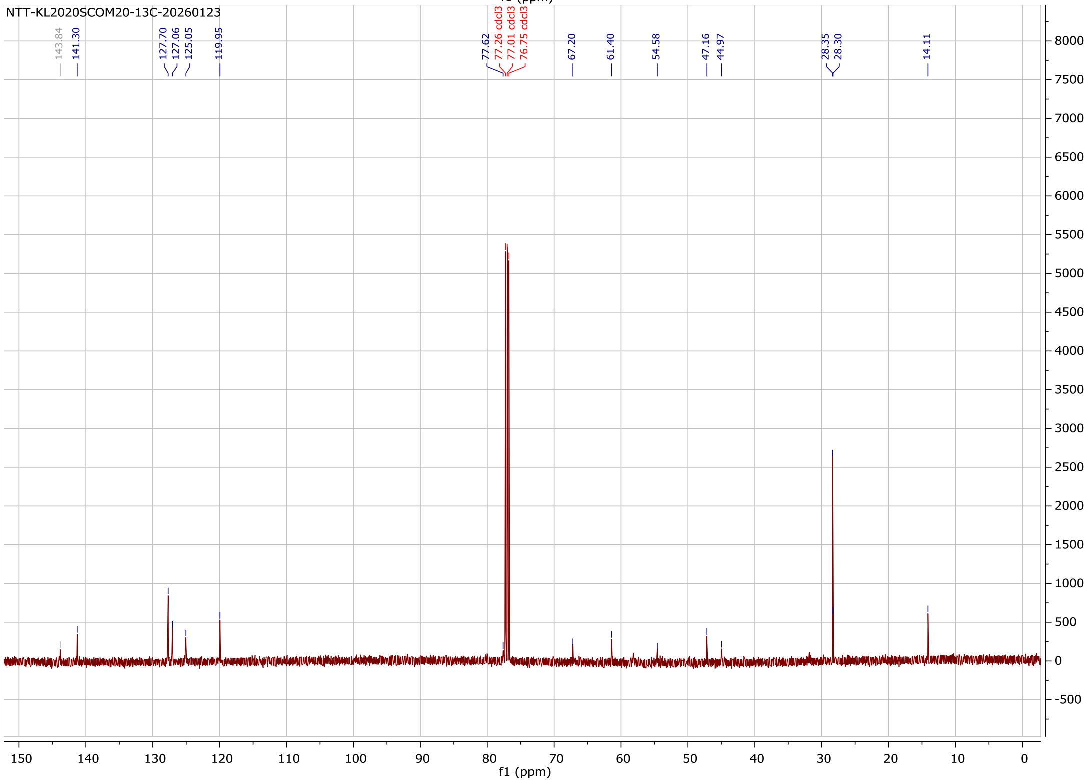

NTT-KL2020SCOM20-13C-20260123
143.84
141.30
127.70
127.06
125.05
119.95
77.62 cdcl3
77.26 cdcl3
77.01 cdcl3
76.75 cdcl3
67.20
61.40
54.58
47.16
44.97
28.35
28.30
14.11
f1 (ppm)

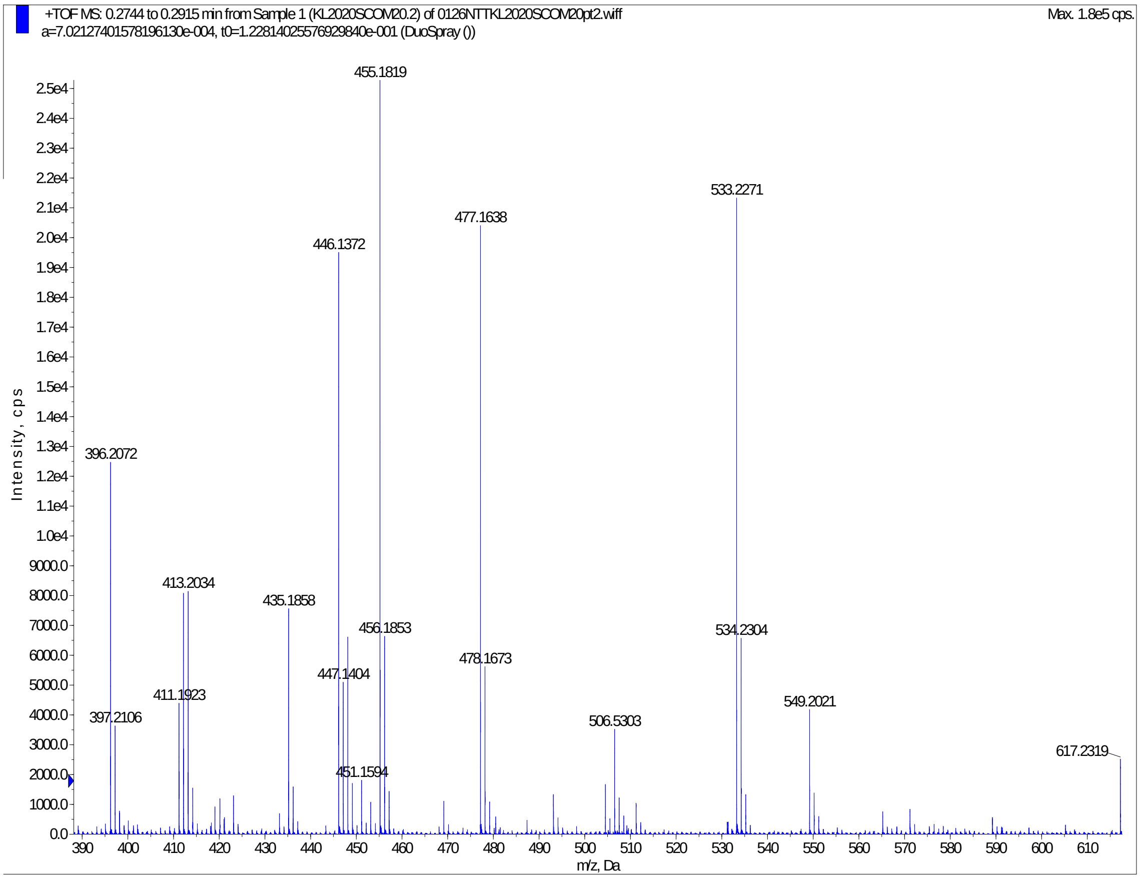
+TOF MS: 0.2744 to 0.2915 min from Sample 1 (KL2020SCOM20.2) of 0126NTTKL2020SCOM20pt2.wiff
a=7.02127401578196130e-004, t0=1.22814025576929840e-001 (DuoSpray ())
Max. 1.8e5 cps.
Intensity, cps
m/z, Da
455.1819
533.2271
477.1638
446.1372
396.2072
413.2034
435.1858
456.1853
534.2304
478.1673
447.1404
411.1923
397.2106
549.2021
506.5303
617.2319
451.1594

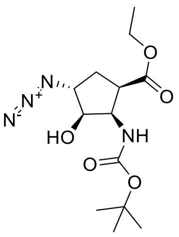
N
N
N
HO
NH
O
O
O
O
O

2

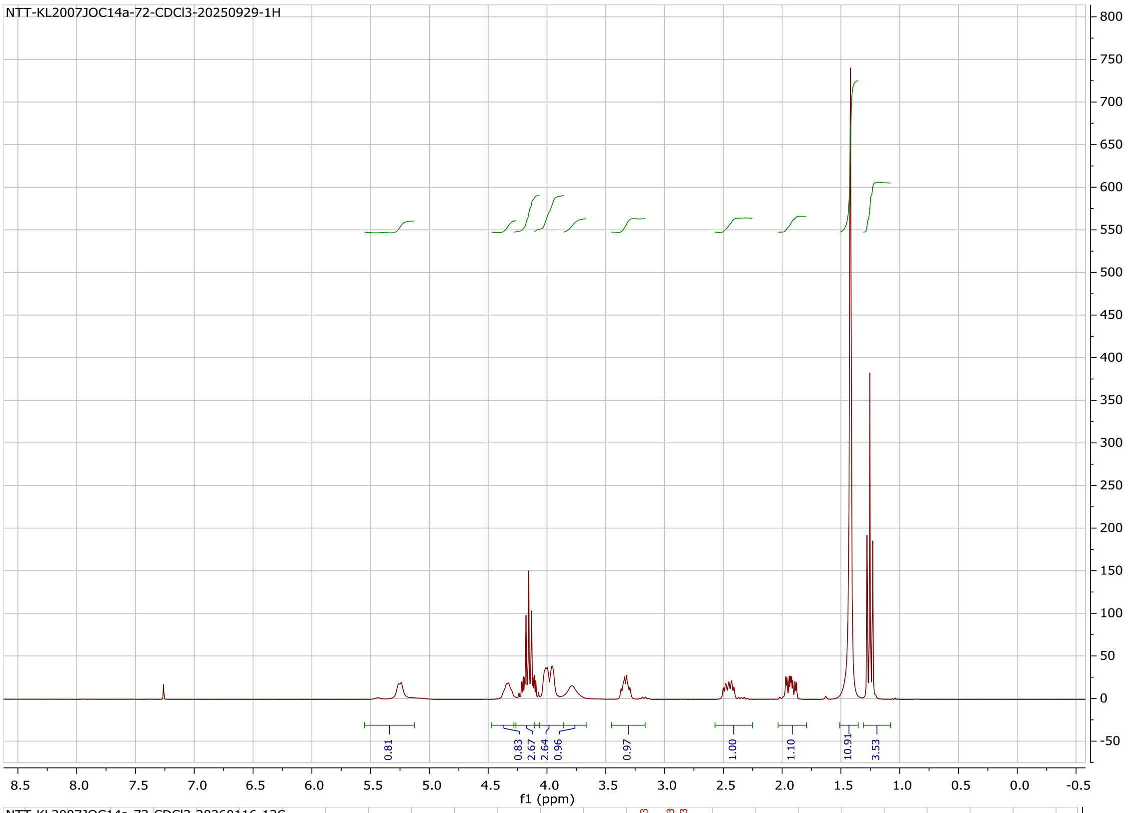
NTT-KL2007JOC14a-72-CDCl3-20250929-1H
0.81
0.83
2.67
2.64
0.96
0.97
1.00
1.10
10.91
3.53
f1 (ppm)

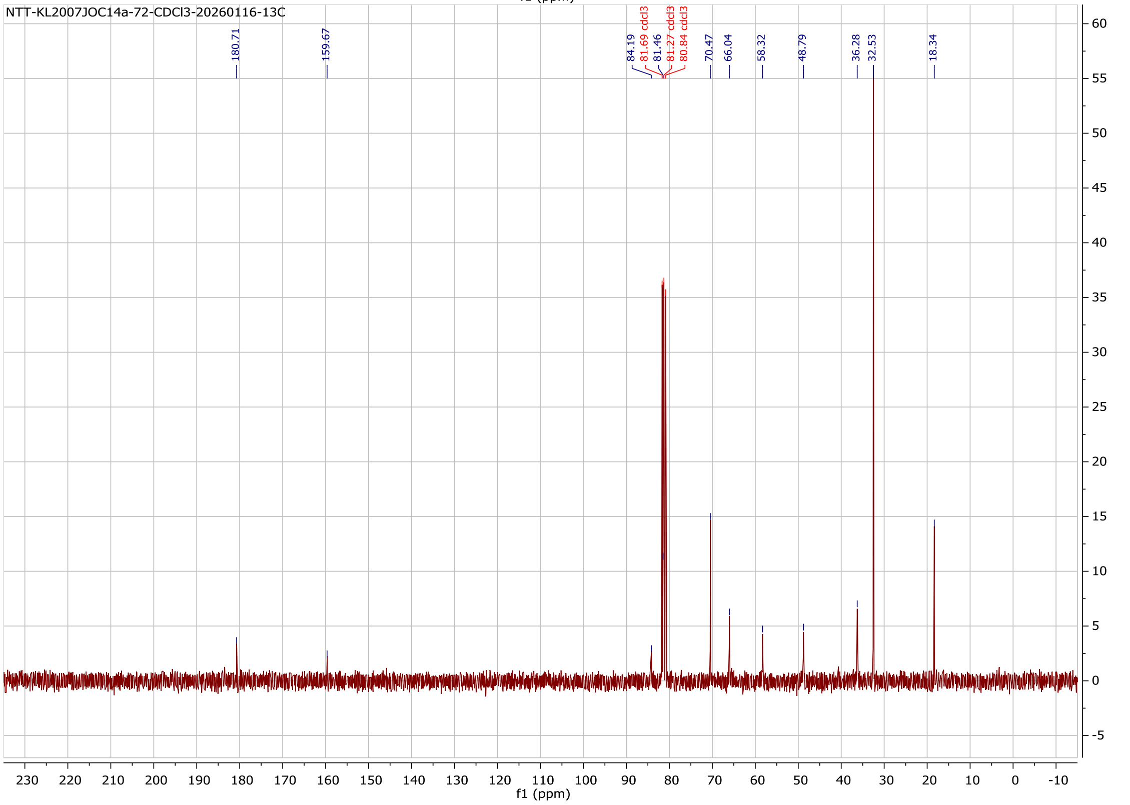
NTT-KL2007JOC14a-72-CDCl3-20260116-13C
180.71
159.67
84.19
81.69 cdcl3
81.46
81.27 cdcl3
80.84 cdcl3
70.47
66.04
58.32
48.79
36.28
32.53
18.34
f1 (ppm)

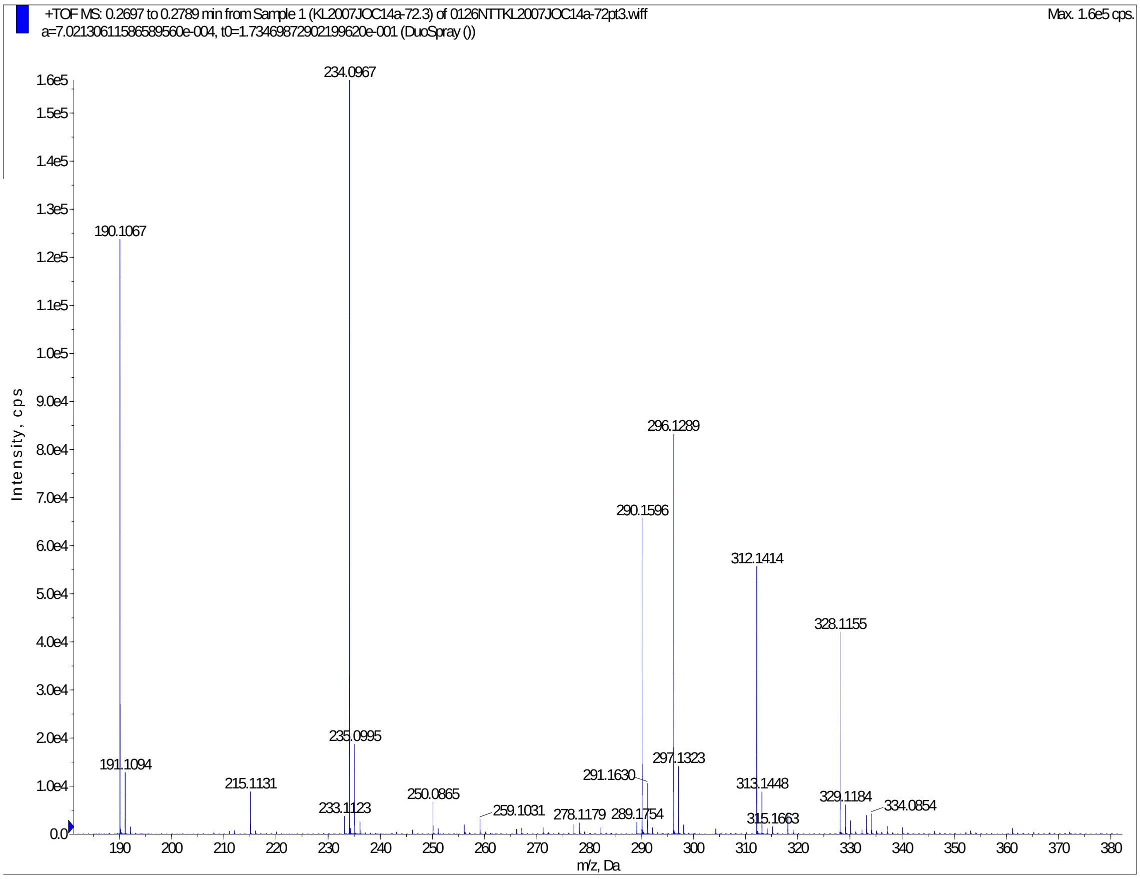
+TOF MS: 0.2697 to 0.2789 min from Sample 1 (KL2007JOC14a-72.3) of 0126NTTKL2007JOC14a-72pt3.wiff
a=7.02130611586589560e-004, t0=1.73469872902199620e-001 (DuoSpray ())
Max. 1.6e5 cps.
Intensity, cps
m/z, Da
234.0967
190.1067
296.1289
290.1596
312.1414
328.1155
235.0995
191.1094
215.1131
297.1323
291.1630
313.1448
250.0865
329.1184
233.1123
259.1031
278.1179
289.1754
315.1663
334.0854

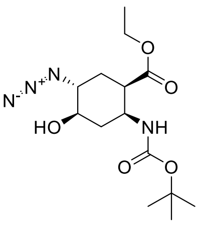

5

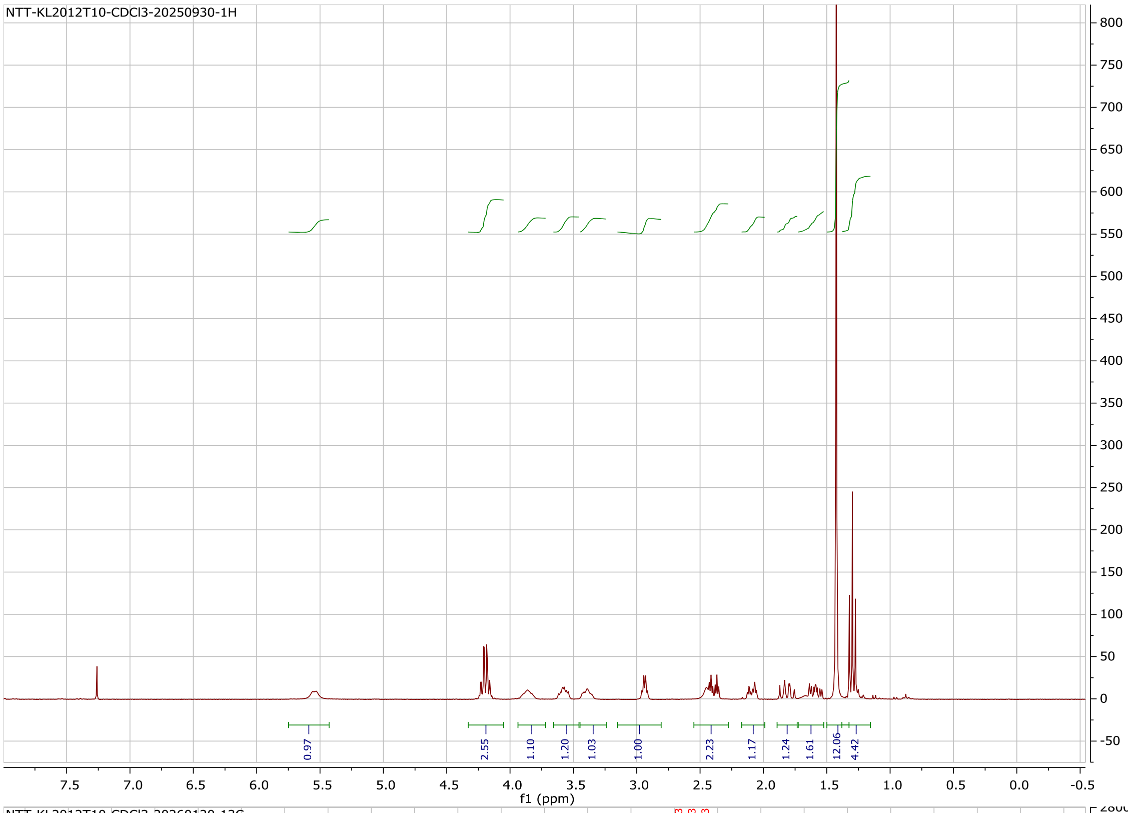
NTT-KL2012T10-CDCl3-20250930-1H
0.97
2.55
1.10
1.20
1.03
1.00
2.23
1.17
1.24
1.61
12.06
4.42
f1 (ppm)

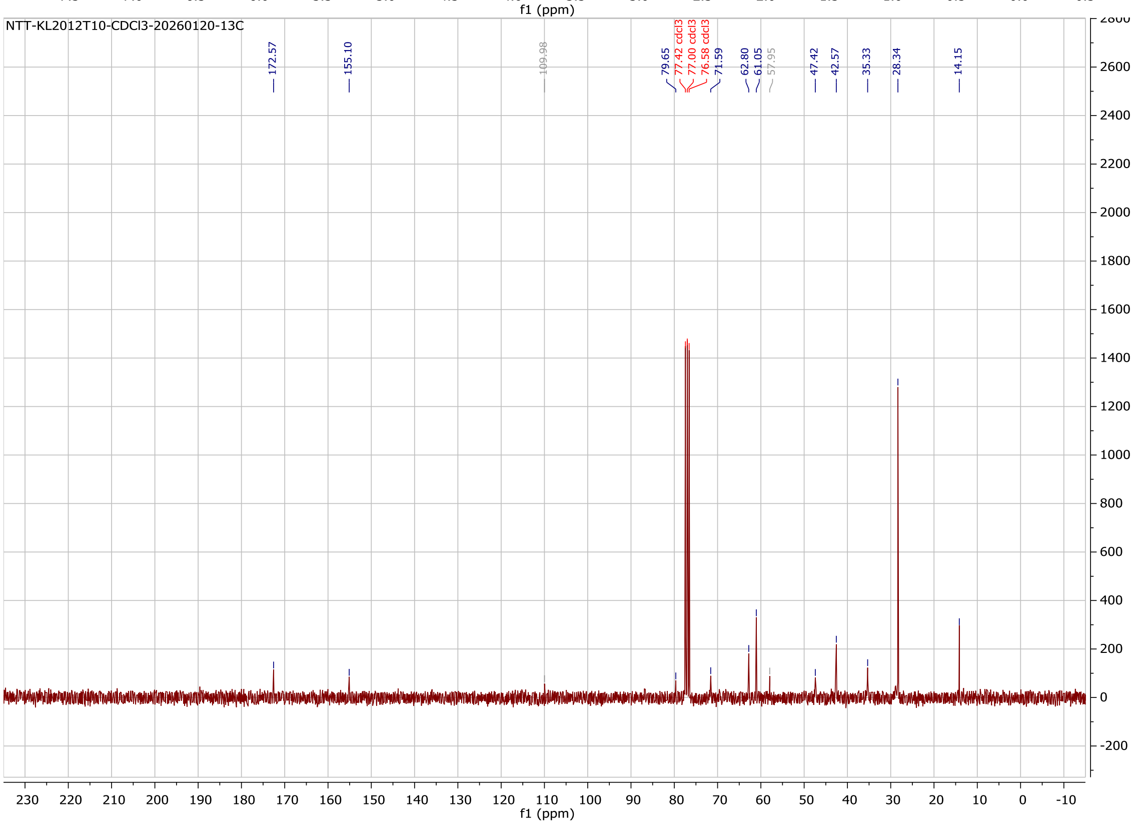
NTT-KL2012T10-CDCl3-20260120-13C
172.57
155.10
109.98
79.65
77.42 cdcl3
77.00 cdcl3
76.58 cdcl3
71.59
62.80
61.05
57.95
47.42
42.57
35.33
28.34
14.15
f1 (ppm)

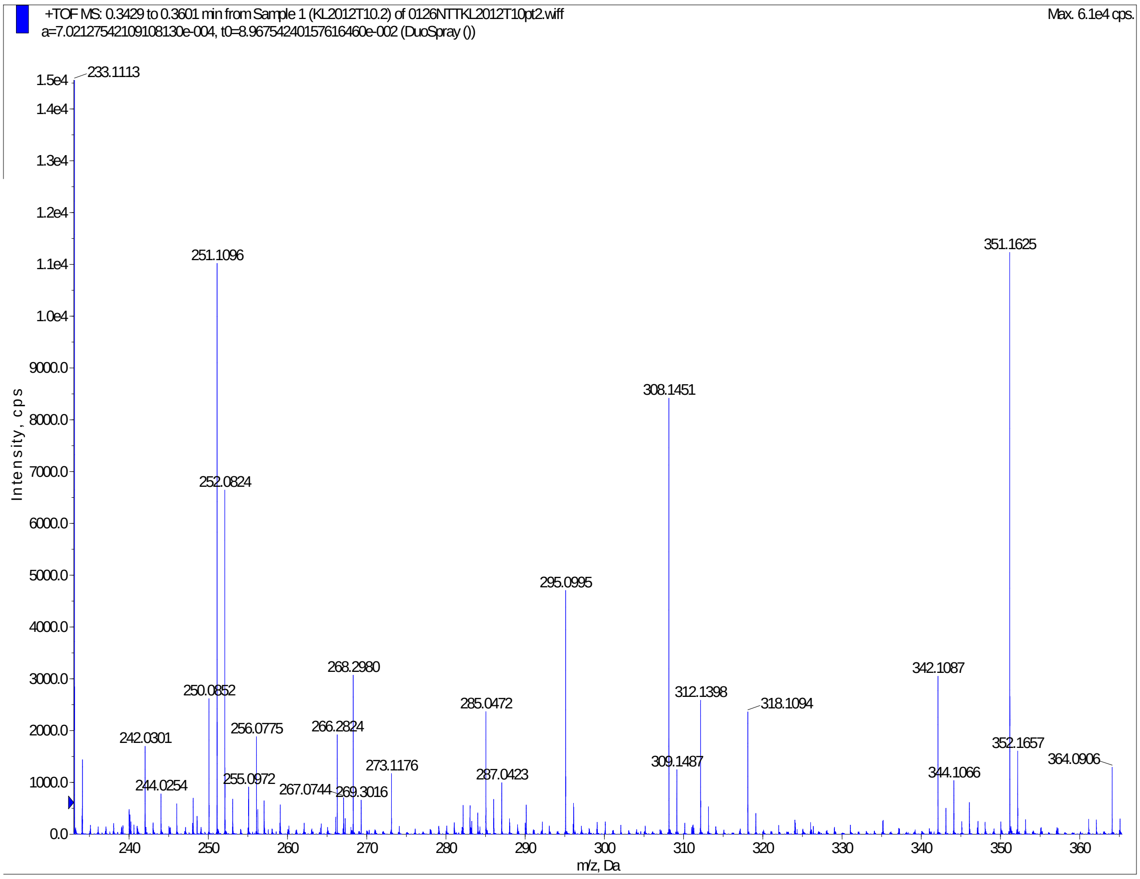
+TOF MS: 0.3429 to 0.3601 min from Sample 1 (KL2012T10.2) of 0126NTTKL2012T10pt2.wiff
a=7.02127542109108130e-004, t0=8.96754240157616460e-002 (DuoSpray ())
Max. 6.1e4 cps.
Intensity, cps
m/z, Da
233.1113
251.1096
351.1625
308.1451
252.0824
295.0995
268.2980
342.1087
250.0852
312.1398
285.0472
318.1094
242.0301
256.0775
266.2824
352.1657
309.1487
364.0906
273.1176
287.0423
344.1066
244.0254
255.0972
267.0744
269.3016

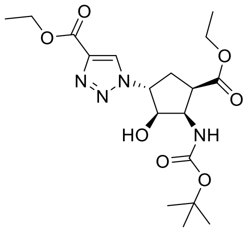
O
O
N
N
N
O
O
HO
NH
O
O

8

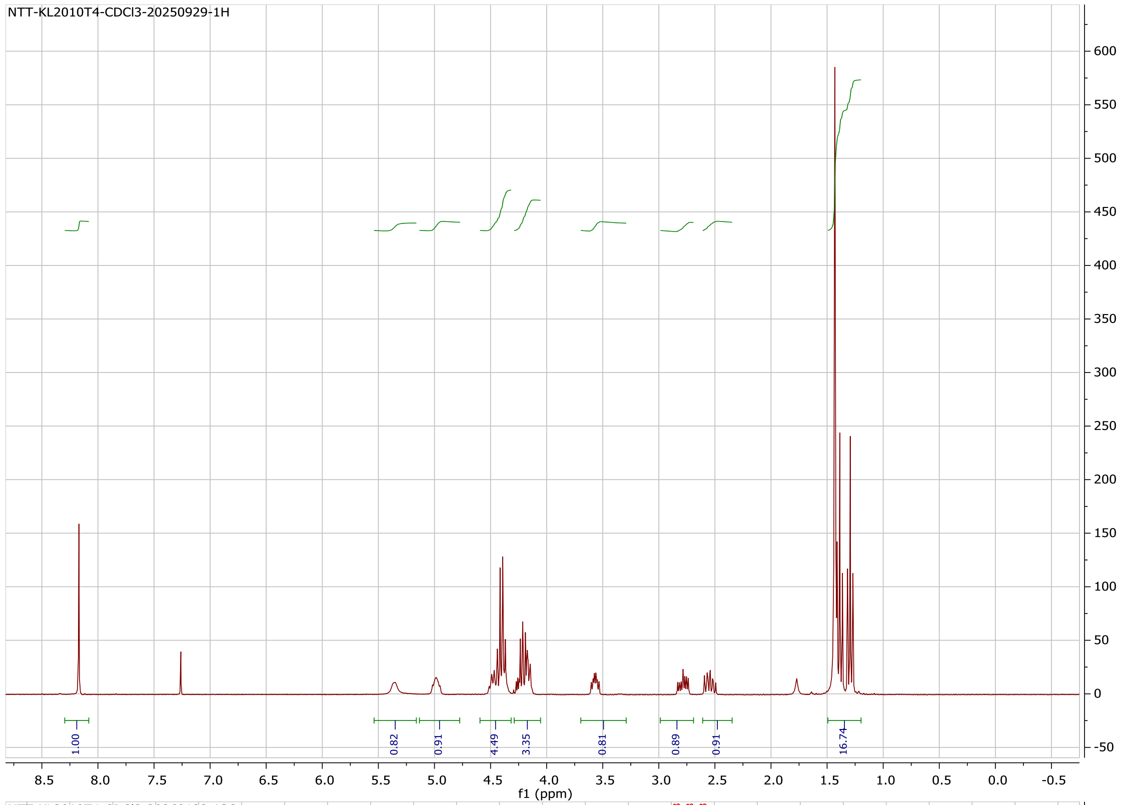
NTT-KL2010T4-CDCl3-20250929-1H
1.00
0.82
0.91
4.49
3.35
0.81
0.89
0.91
16.74
f1 (ppm)

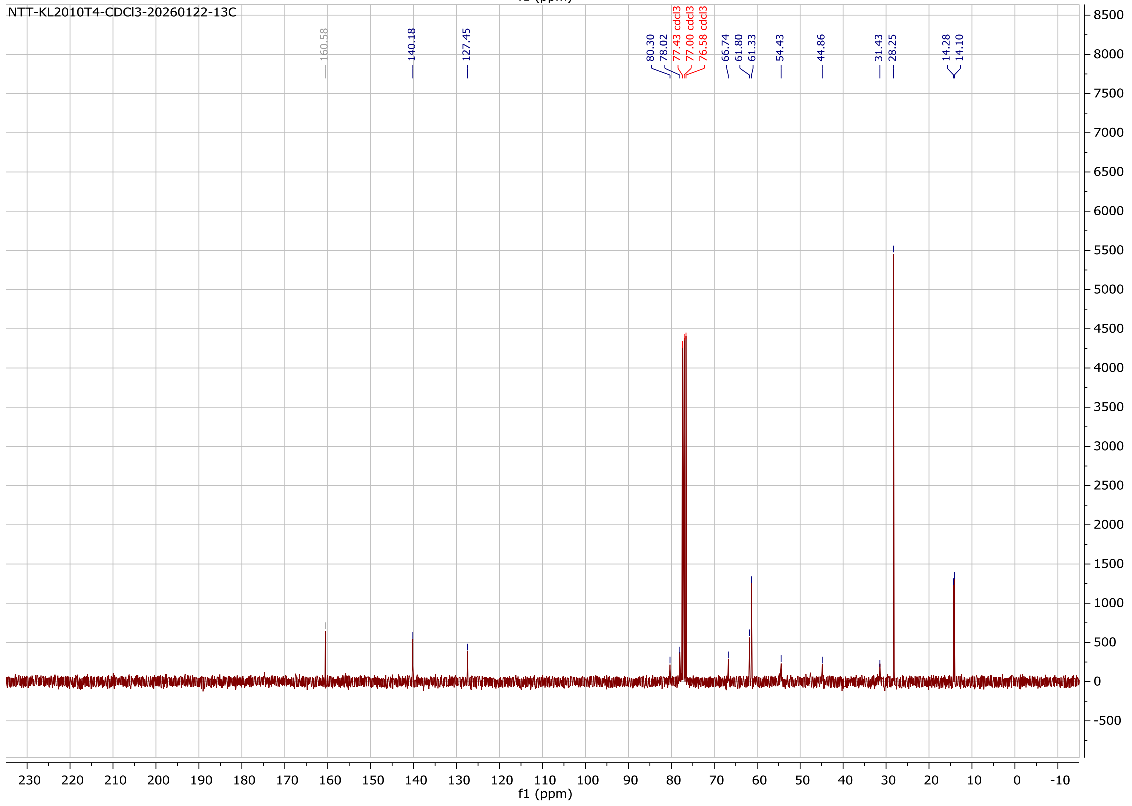
NTT-KL2010T4-CDCl3-20260122-13C
160.58
140.18
127.45
80.30
78.02
77.43 cdcl3
77.00 cdcl3
76.58 cdcl3
66.74
61.80
61.33
54.43
44.86
31.43
28.25
14.28
14.10
f1 (ppm)

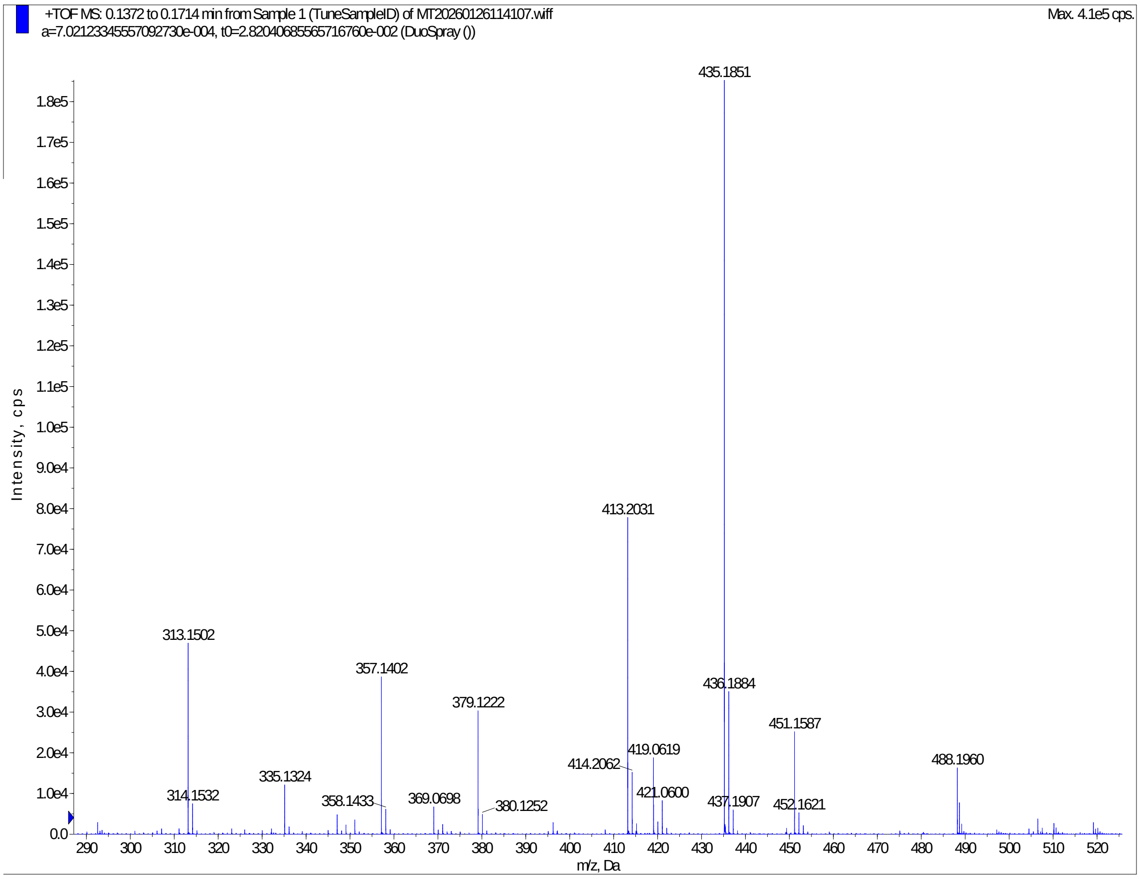
+TOF MS: 0.1372 to 0.1714 min from Sample 1 (TuneSampleID) of MT20260126114107.wiff
a=7.02123345557092730e-004, t0=2.82040685565716760e-002 (DuoSpray ())
Max. 4.1e5 cps.
Intensity, cps
m/z, Da
435.1851
413.2031
313.1502
357.1402
379.1222
436.1884
451.1587
419.0619
414.2062
488.1960
335.1324
421.0600
314.1532
358.1433
369.0698
380.1252
437.1907
452.1621

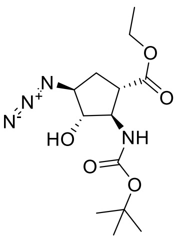

3

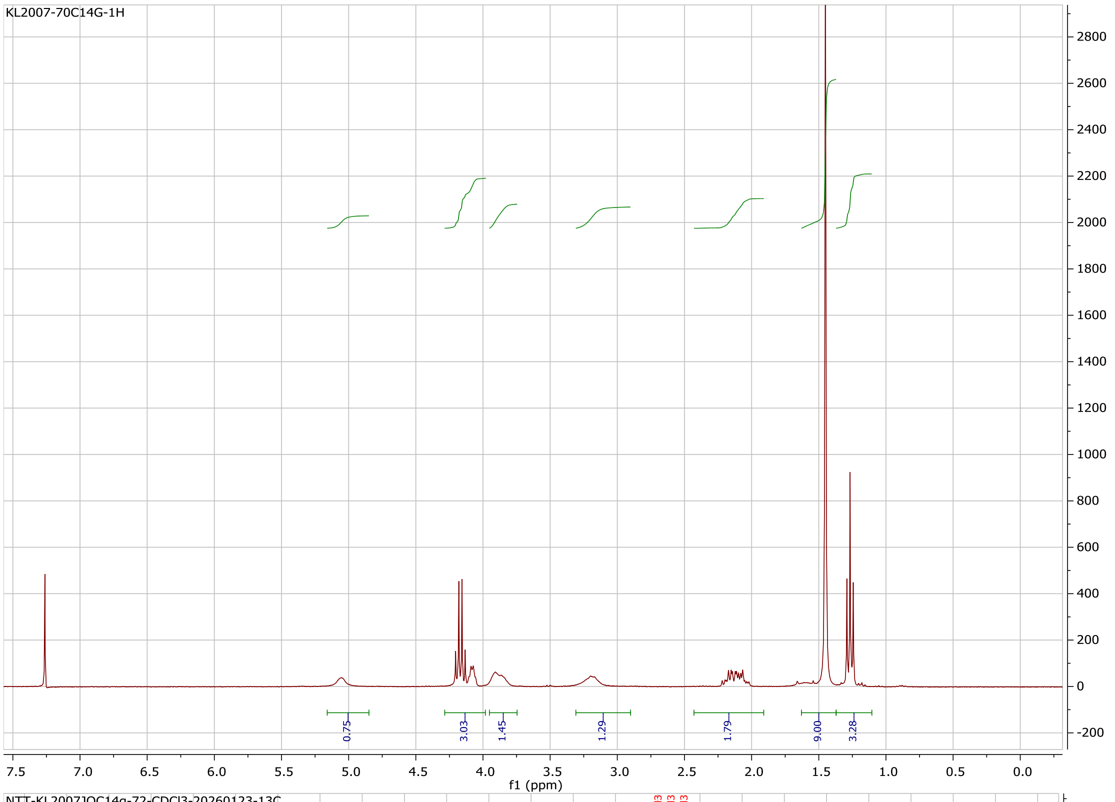
KL2007-70C14G-1H
0.75
3.03
1.45
1.29
1.79
9.00
3.28
f1 (ppm)

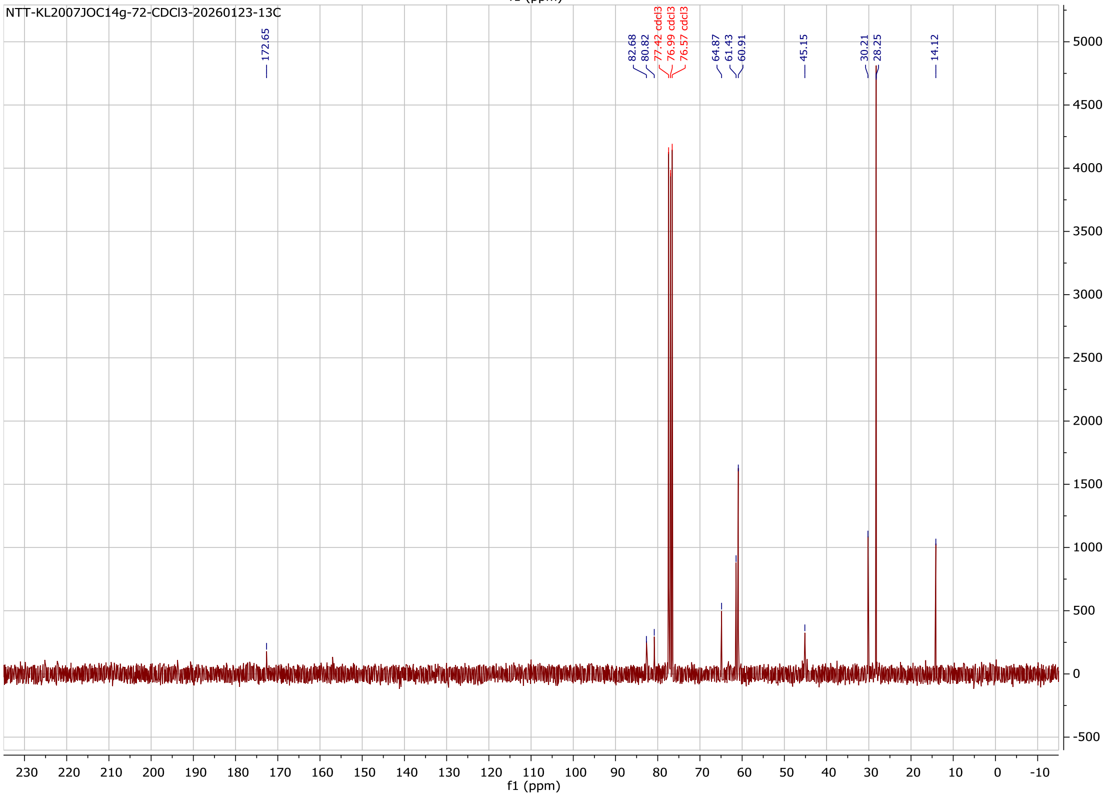
NTT-KL2007JOC14g-72-CDCl3-20260123-13C
172.65
82.68
80.82
77.42 cdcl3
76.99 cdcl3
76.57 cdcl3
64.87
61.43
60.91
45.15
30.21
28.25
14.12
f1 (ppm)

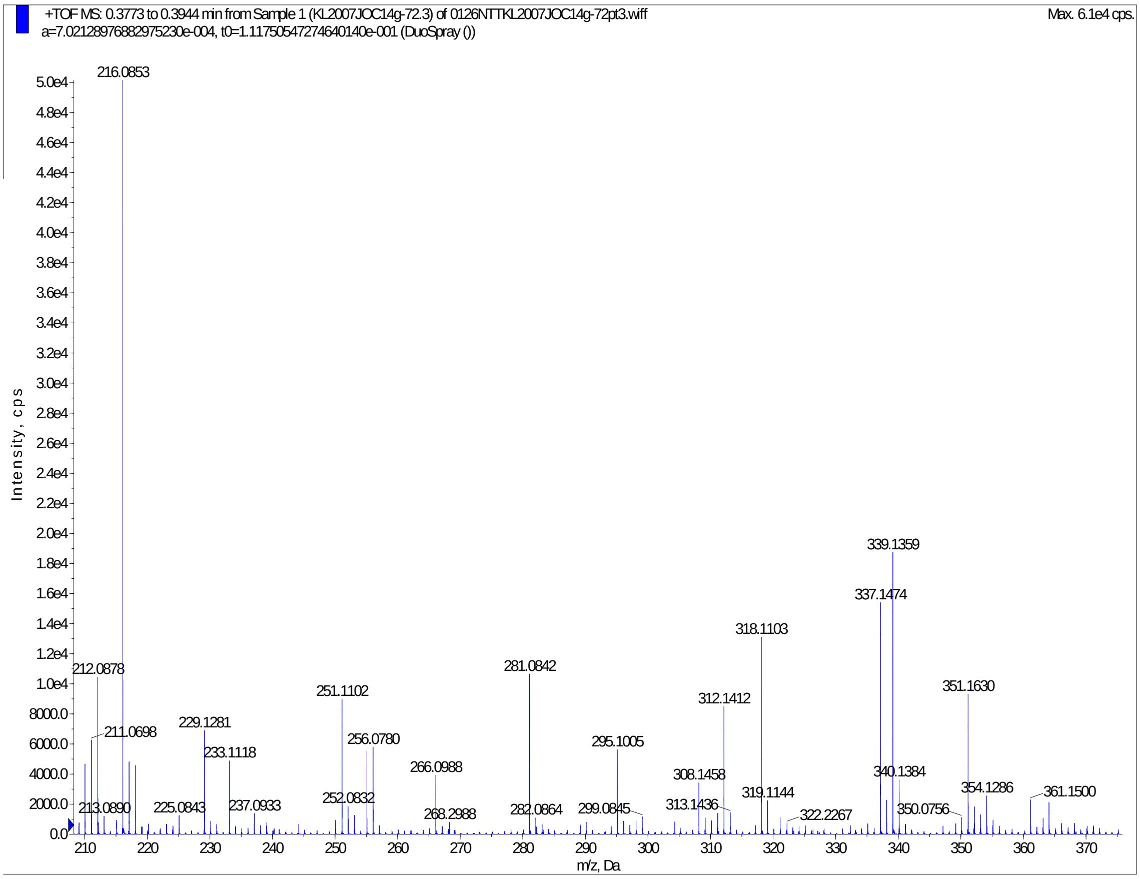
+TOF MS: 0.3773 to 0.3944 min from Sample 1 (KL2007JOC14g-72.3) of 0126NTTKL2007JOC14g-72pt3.wiff
a=7.02128976882975230e-004, t0=1.11750547274640140e-001 (DuoSpray ())
Max. 6.1e4 cps.
Intensity, cps
m/z, Da
216.0853
339.1359
337.1474
318.1103
281.0842
212.0878
251.1102
312.1412
351.1630
211.0698
229.1281
256.0780
295.1005
233.1118
266.0988
308.1458
340.1384
354.1286
361.1500
252.0832
319.1144
213.0890
225.0843
237.0933
313.1436
282.0864
299.0845
350.0756
322.2267
268.2988

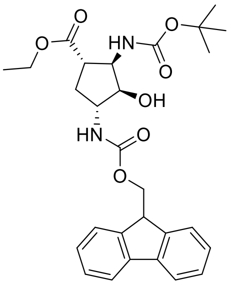
O
O
HN
O
O
OH
HN
O
O

6

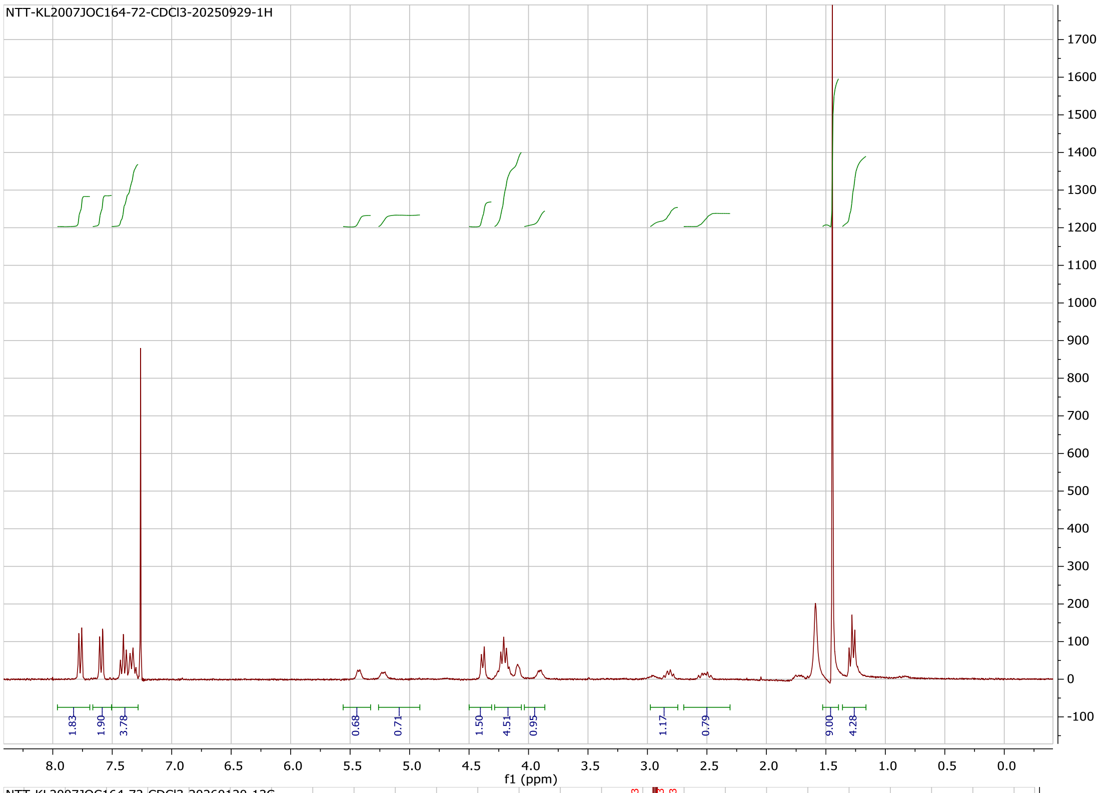
NTT-KL2007JOC164-72-CDCl3-20250929-1H
1.83
1.90
3.78
0.68
0.71
1.50
4.51
0.95
1.17
0.79
9.00
4.28
f1 (ppm)

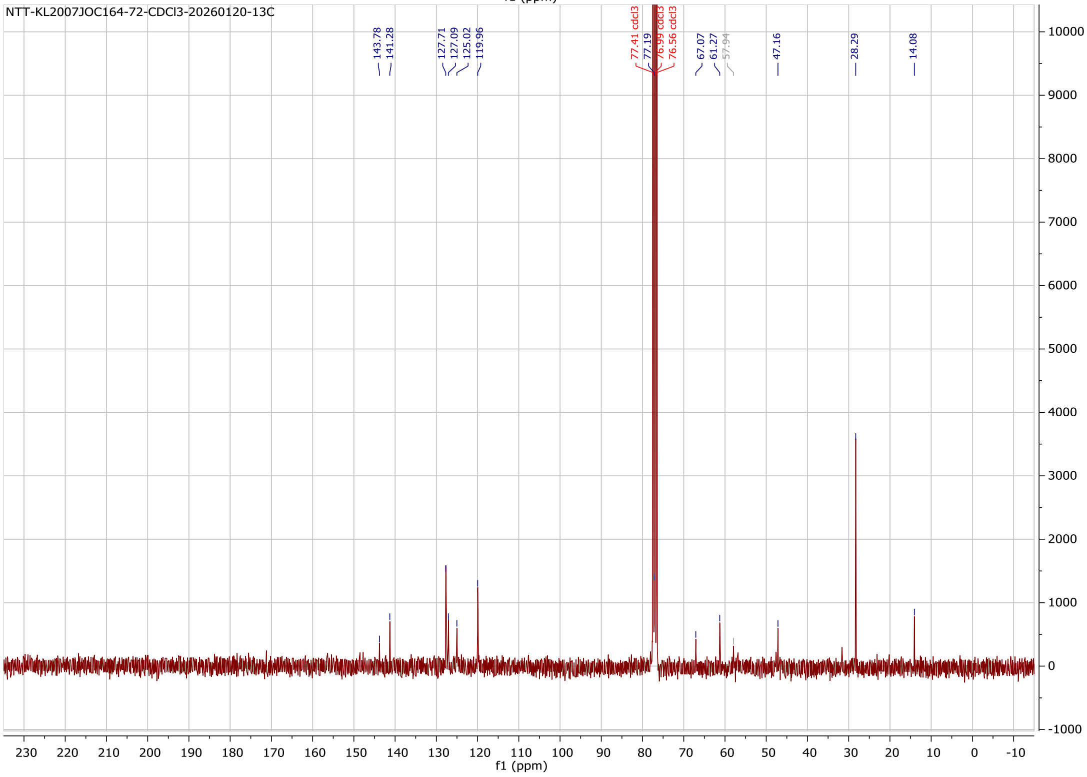
NTT-KL2007JOC164-72-CDCl3-20260120-13C
143.78
141.28
127.71
127.09
125.02
119.96
77.41 cdcl3
77.19
76.99 cdcl3
76.56 cdcl3
67.07
61.27
57.94
47.16
28.29
14.08
f1 (ppm)

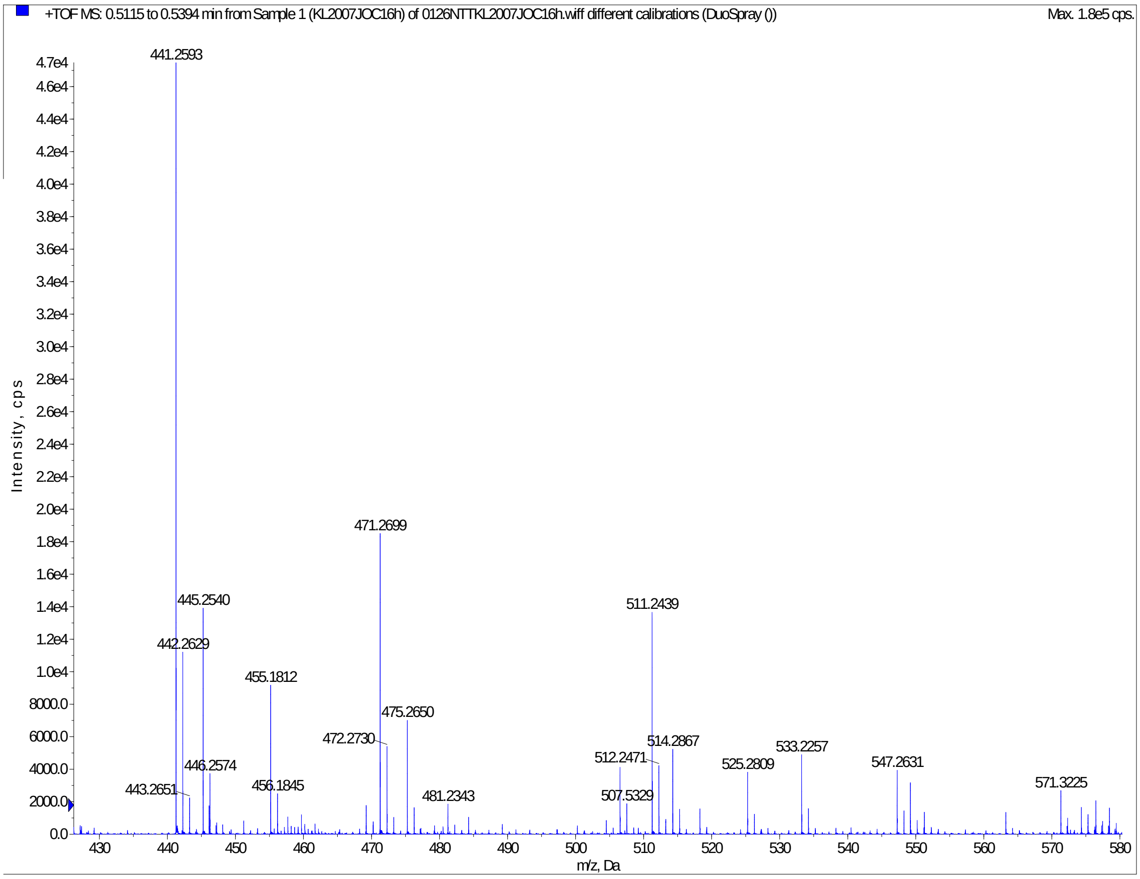
+TOF MS: 0.5115 to 0.5394 min from Sample 1 (KL2007JOC16h) of 0126NTTKL2007JOC16h.wiff different calibrations (DuoSpray ())
Max. 1.8e5 cps.
Intensity, cps
m/z, Da
441.2593
471.2699
445.2540
511.2439
442.2629
455.1812
475.2650
472.2730
514.2867
512.2471
533.2257
525.2809
547.2631
446.2574
443.2651
456.1845
481.2343
507.5329
571.3225

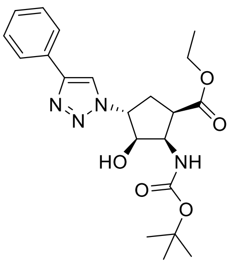

9

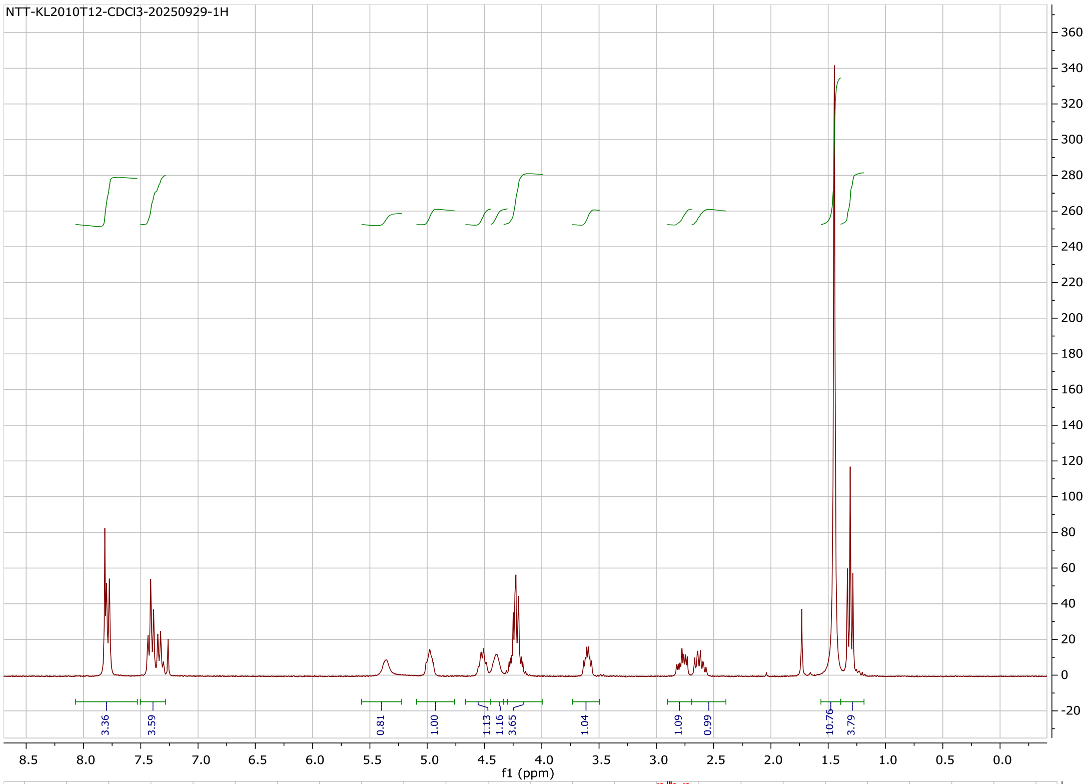

NTT-KL2010T12-CDCl3-20250929-1H
3.36
3.59
0.81
1.00
1.13
1.16
3.65
1.04
1.09
0.99
10.76
3.79
f1 (ppm)

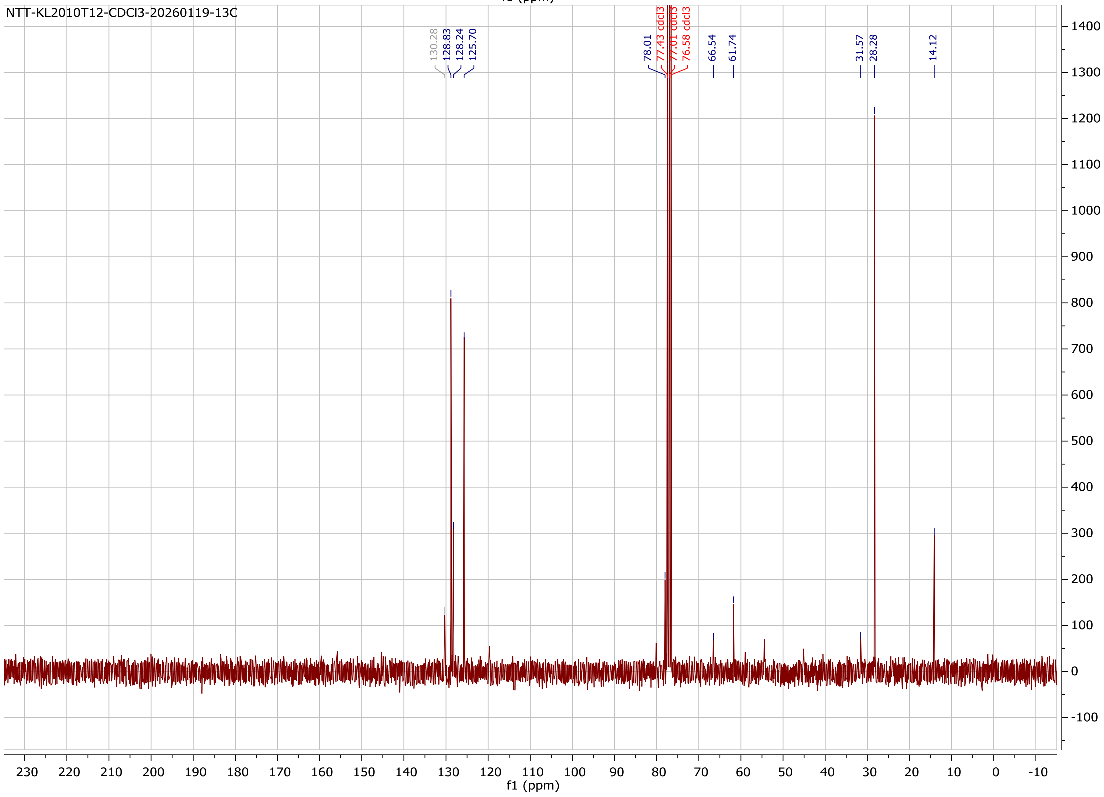

NTT-KL2010T12-CDCl3-20260119-13C
130.28
128.83
128.24
125.70
78.01
77.43 cdcl3
77.01 cdcl3
76.58 cdcl3
66.54
61.74
31.57
28.28
14.12
f1 (ppm)

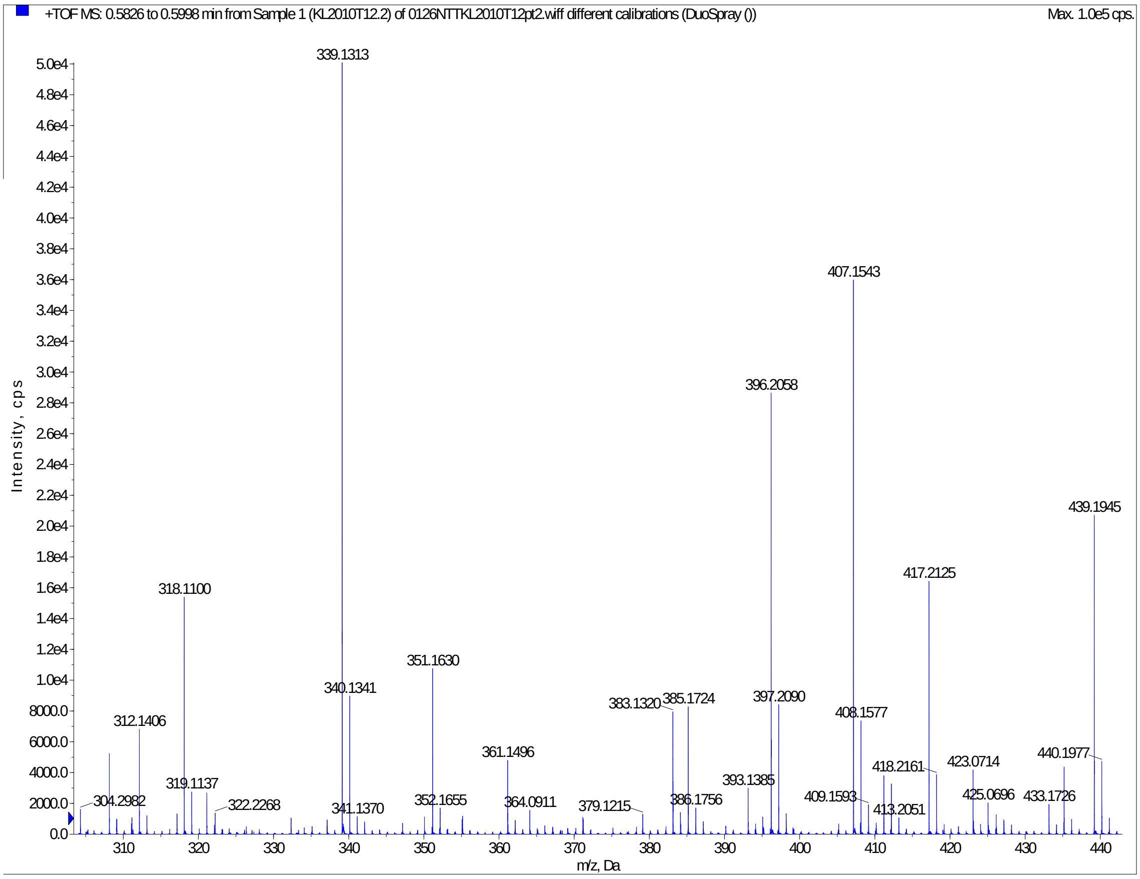
+TOF MS: 0.5826 to 0.5998 min from Sample 1 (KL2010T12.2) of 0126NTTKL2010T12pt2.wiff different calibrations (DuoSpray ())
Max. 1.0e5 cps.
Intensity, cps
m/z, Da
339.1313
407.1543
396.2058
439.1945
417.2125
318.1100
351.1630
340.1341
383.1320
385.1724
397.2090
408.1577
312.1406
361.1496
418.2161
423.0714
440.1977
319.1137
393.1385
304.2982
322.2268
341.1370
352.1655
364.0911
379.1215
386.1756
409.1593
413.2051
425.0696
433.1726

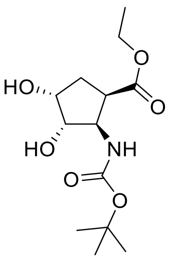

10

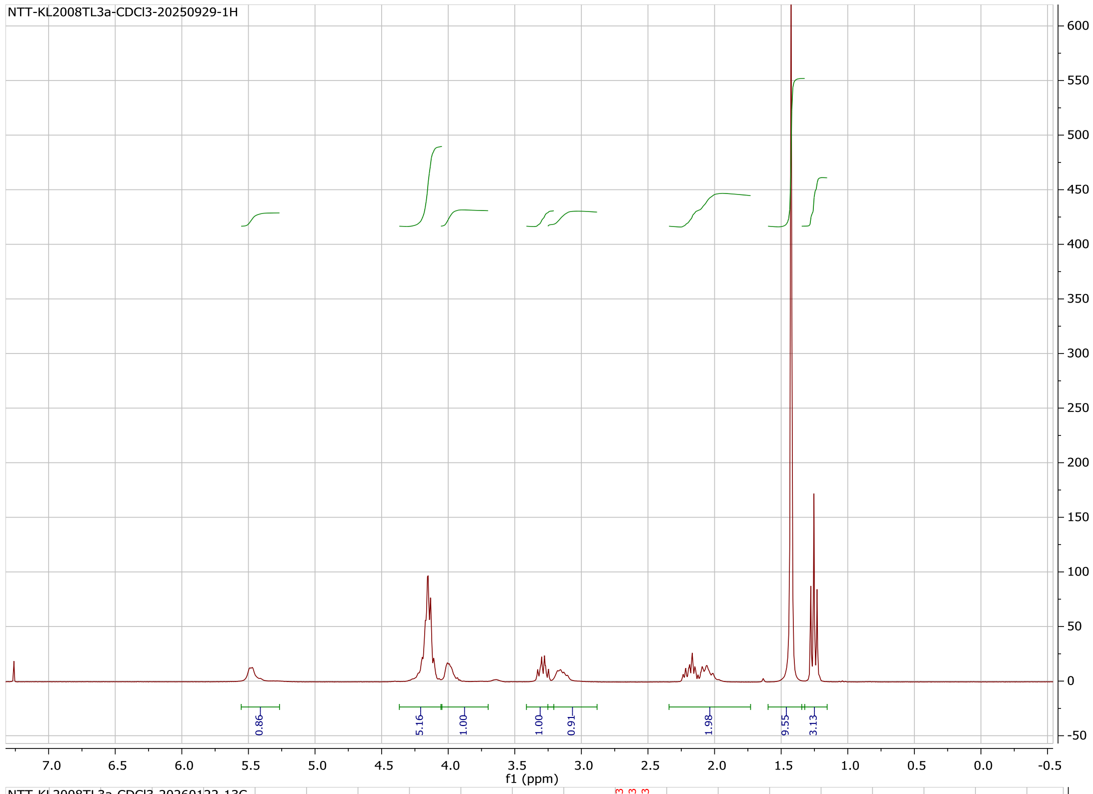
NTT-KL2008TL3a-CDCl3-20250929-1H
0.86
5.16
1.00
1.00
0.91
1.98
9.55
3.13
f1 (ppm)

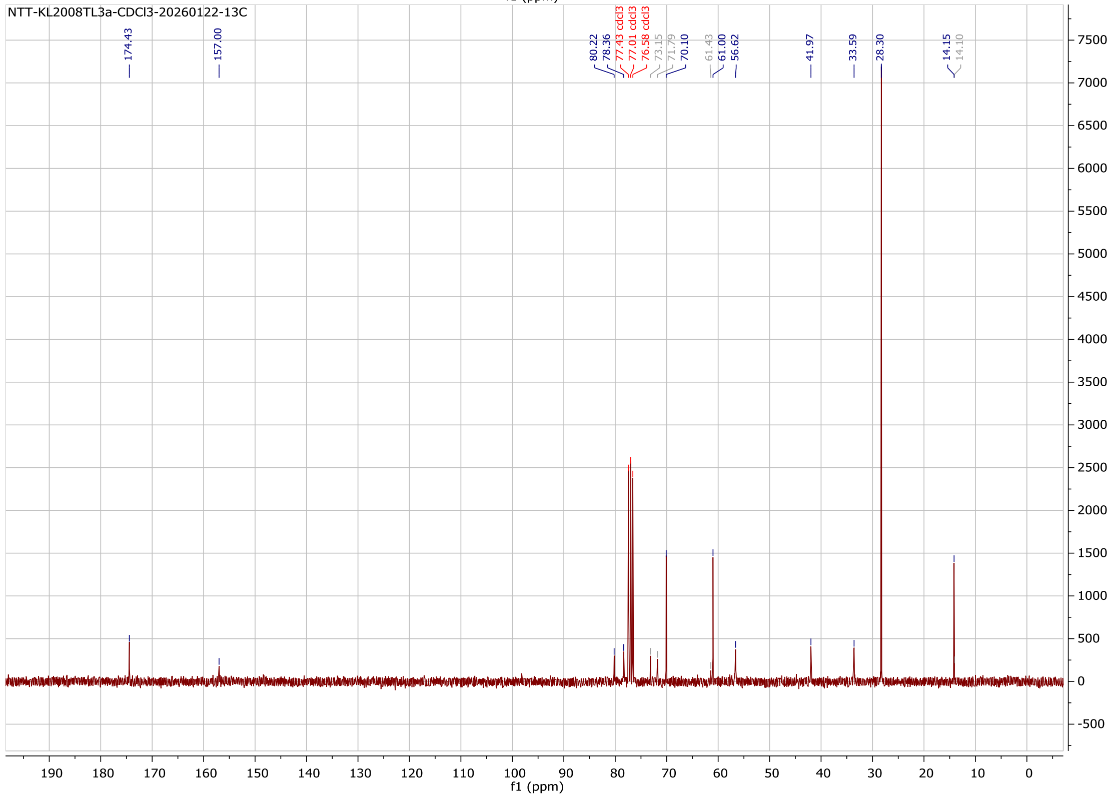
NTT-KL2008TL3a-CDCl3-20260122-13C
174.43
157.00
80.22
78.36
77.43 cdcl3
77.01 cdcl3
76.58 cdcl3
73.15
71.79
70.10
61.43
61.00
56.62
41.97
33.59
28.30
14.15
14.10
f1 (ppm)

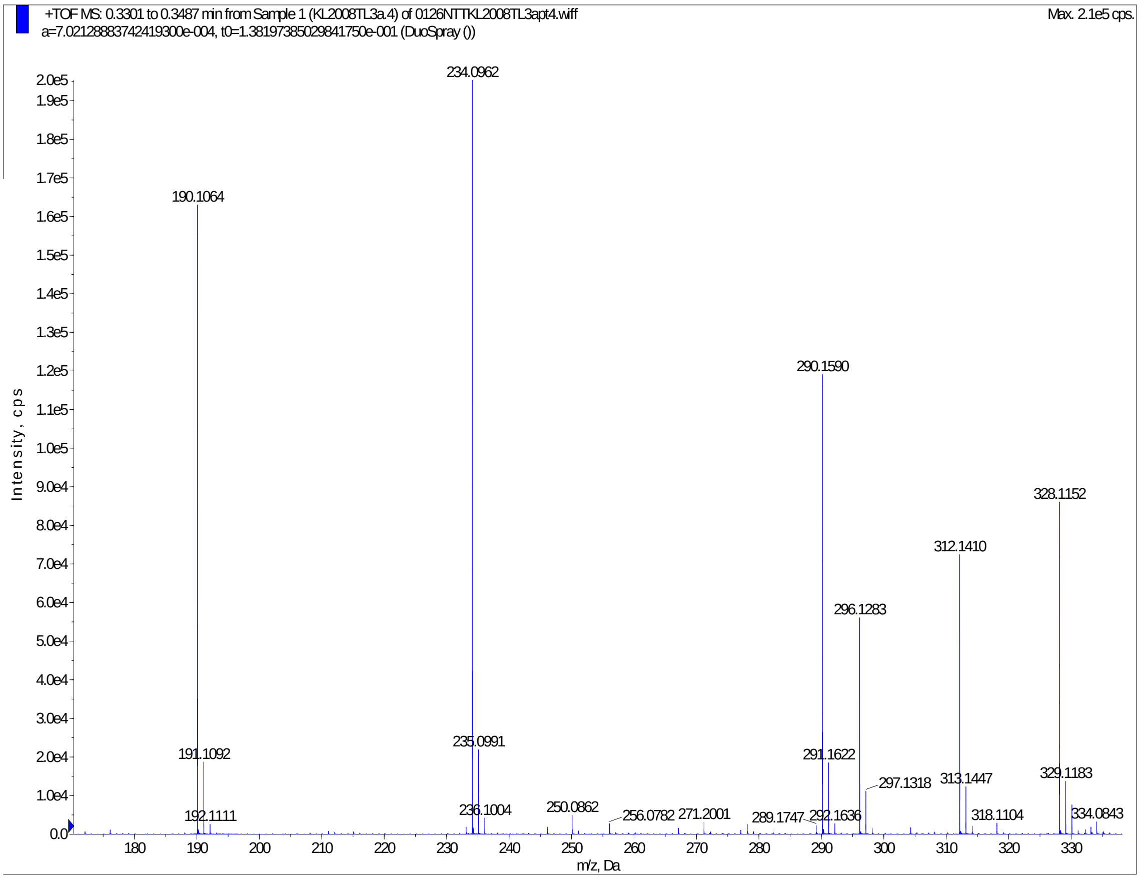

+TOF MS: 0.3301 to 0.3487 min from Sample 1 (KL2008TL3a.4) of 0126NTTKL2008TL3apt4.wiff
a=7.02128883742419300e-004, t0=1.38197385029841750e-001 (DuoSpray ())
Max. 2.1e5 cps.
Intensity, cps
m/z, Da
234.0962
190.1064
290.1590
328.1152
312.1410
296.1283
235.0991
191.1092
291.1622
329.1183
297.1318
313.1447
192.1111
236.1004
250.0862
256.0782
271.2001
289.1747
292.1636
318.1104
334.0843

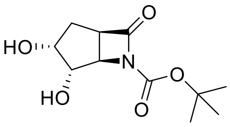

12

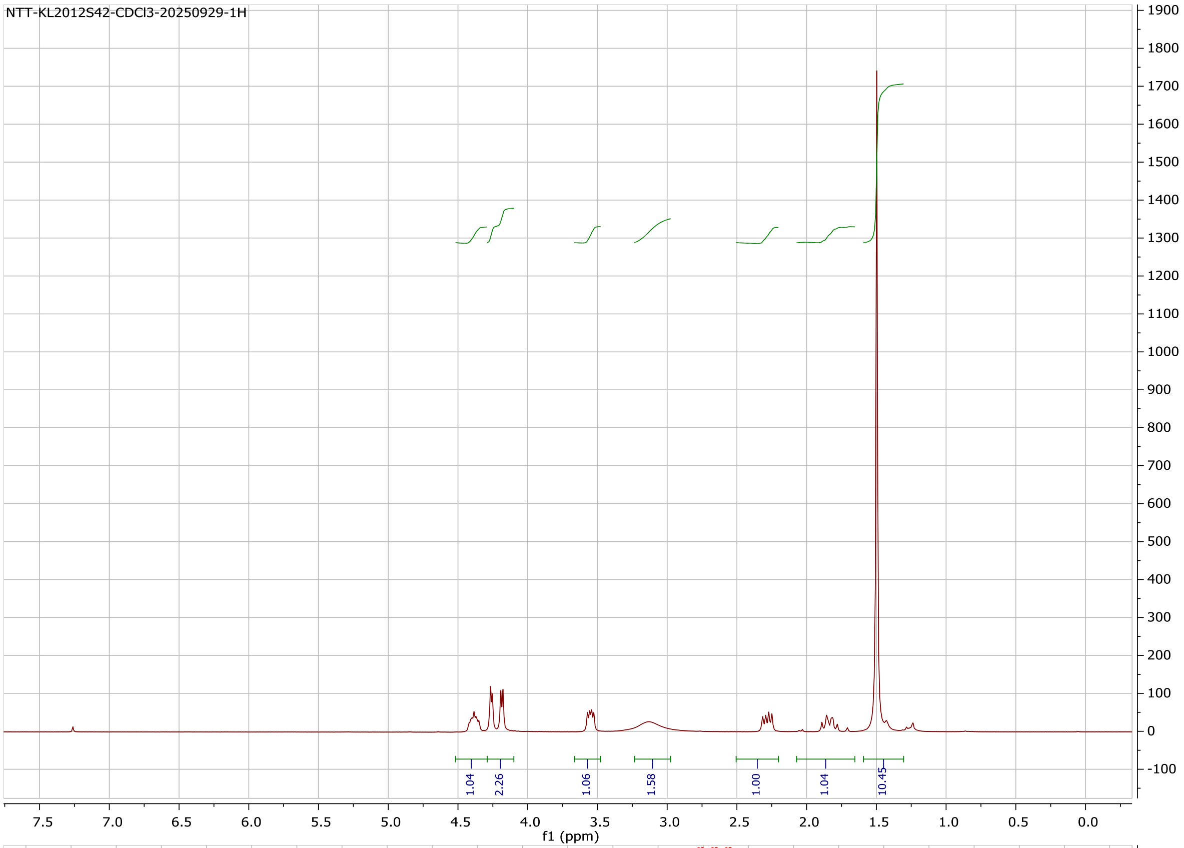
NTT-KL2012S42-CDCl3-20250929-1H
1.04
2.26
1.06
1.58
1.00
1.04
10.45
f1 (ppm)

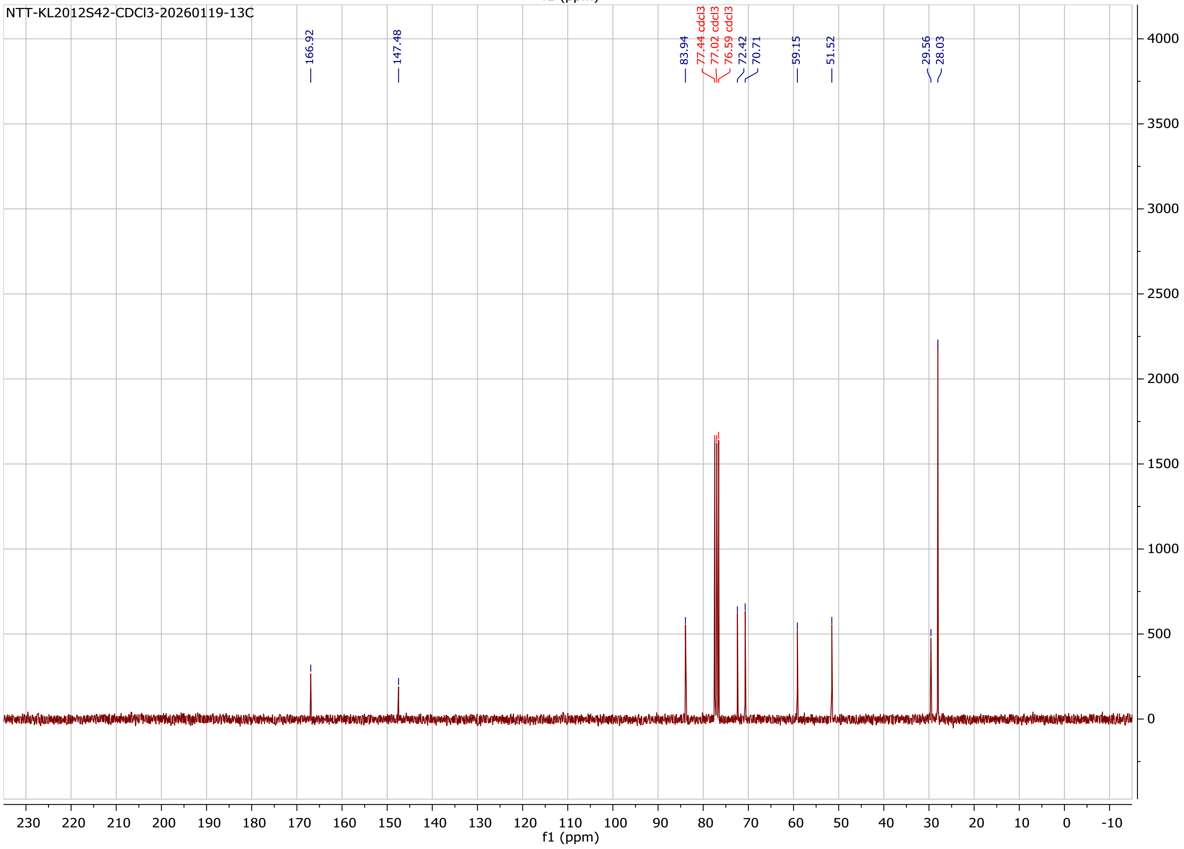
NTT-KL2012S42-CDCl3-20260119-13C
166.92
147.48
83.94
77.44 cdcl3
77.02 cdcl3
76.59 cdcl3
72.42
70.71
59.15
51.52
29.56
28.03
f1 (ppm)

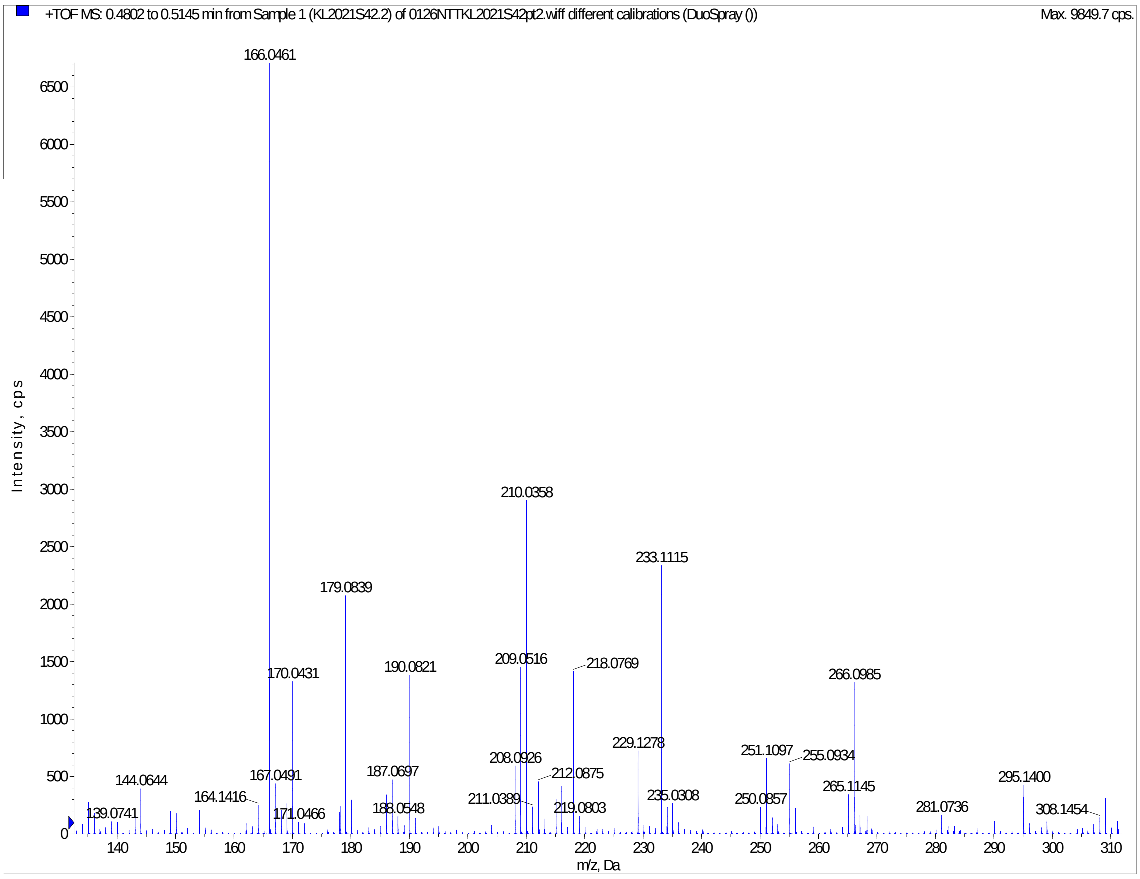
+TOF MS: 0.4802 to 0.5145 min from Sample 1 (KL2021S42.2) of 0126NTTKL2021S42pt2.wiff different calibrations (DuoSpray ())
Max. 9849.7 cps.
Intensity, cps
m/z, Da
166.0461
210.0358
233.1115
179.0839
190.0821
209.0516
218.0769
170.0431
266.0985
229.1278
251.1097
255.0934
208.0926
187.0697
212.0875
167.0491
144.0644
295.1400
265.1145
164.1416
211.0389
235.0308
250.0857
188.0548
219.0803
281.0736
139.0741
171.0466
308.1454

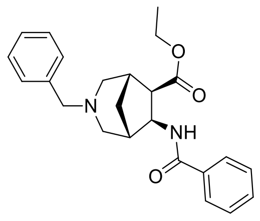
NH
N
O
O
O
14

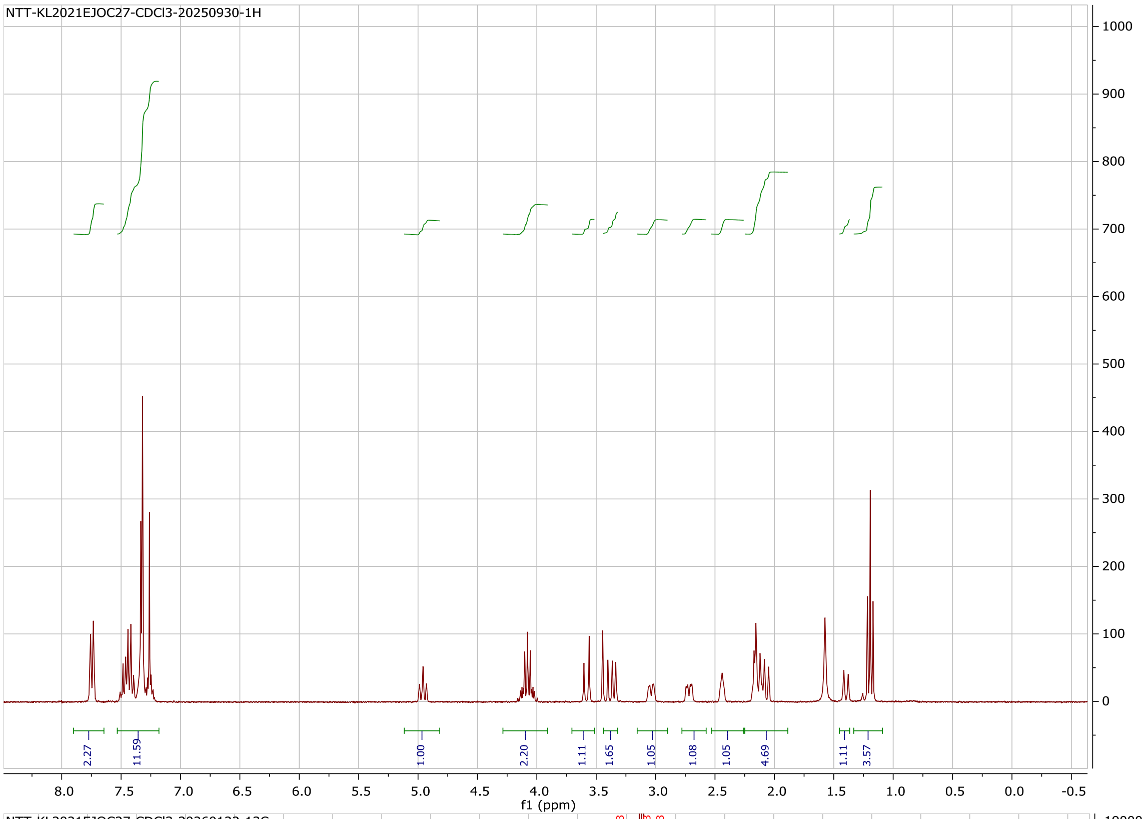
NTT-KL2021EJOC27-CDCl3-20250930-1H
2.27
11.59
1.00
2.20
1.11
1.65
1.05
1.08
1.05
4.69
1.11
3.57
f1 (ppm)

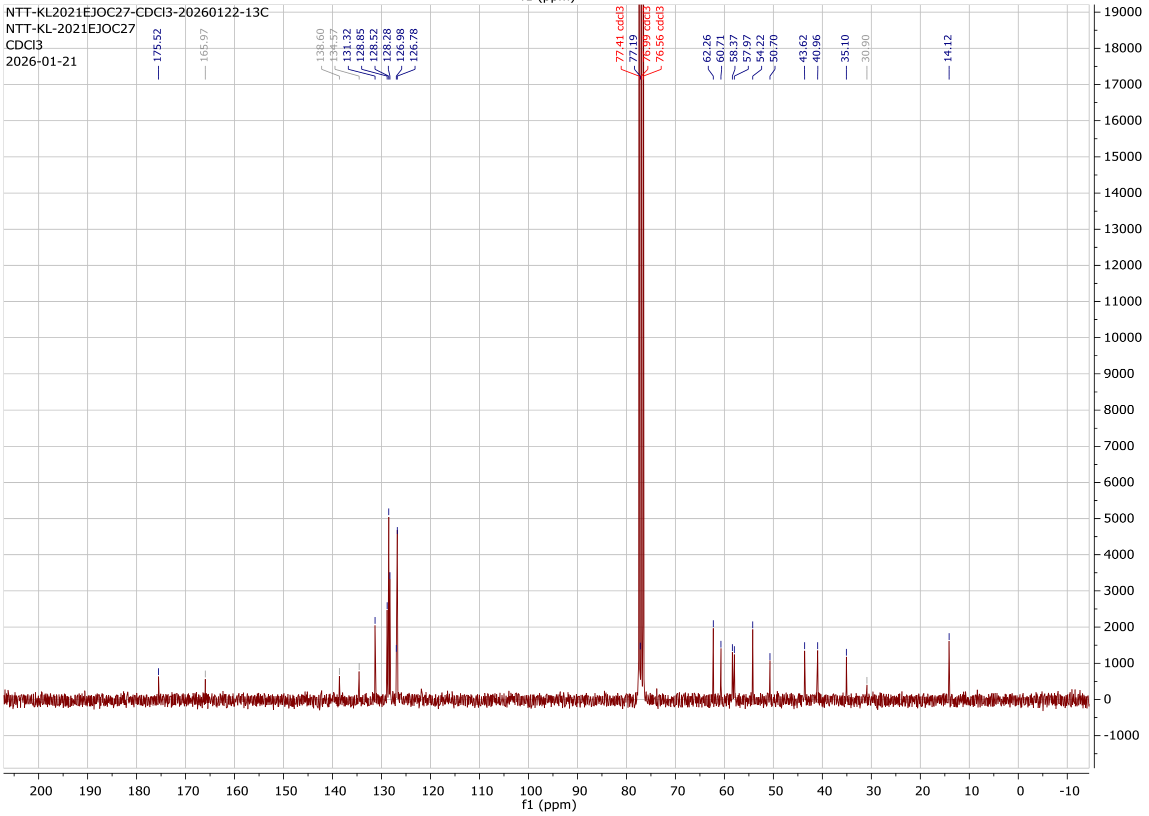
NTT-KL2021EJOC27-CDCl3-20260122-13C
NTT-KL-2021EJOC27
CDCl3
2026-01-21
175.52
165.97
138.60
134.57
131.32
128.85
128.52
128.28
126.98
126.78
77.41 cdcl3
77.19
76.99 cdcl3
76.56 cdcl3
62.26
60.71
58.37
57.97
54.22
50.70
43.62
40.96
35.10
30.90
14.12
f1 (ppm)

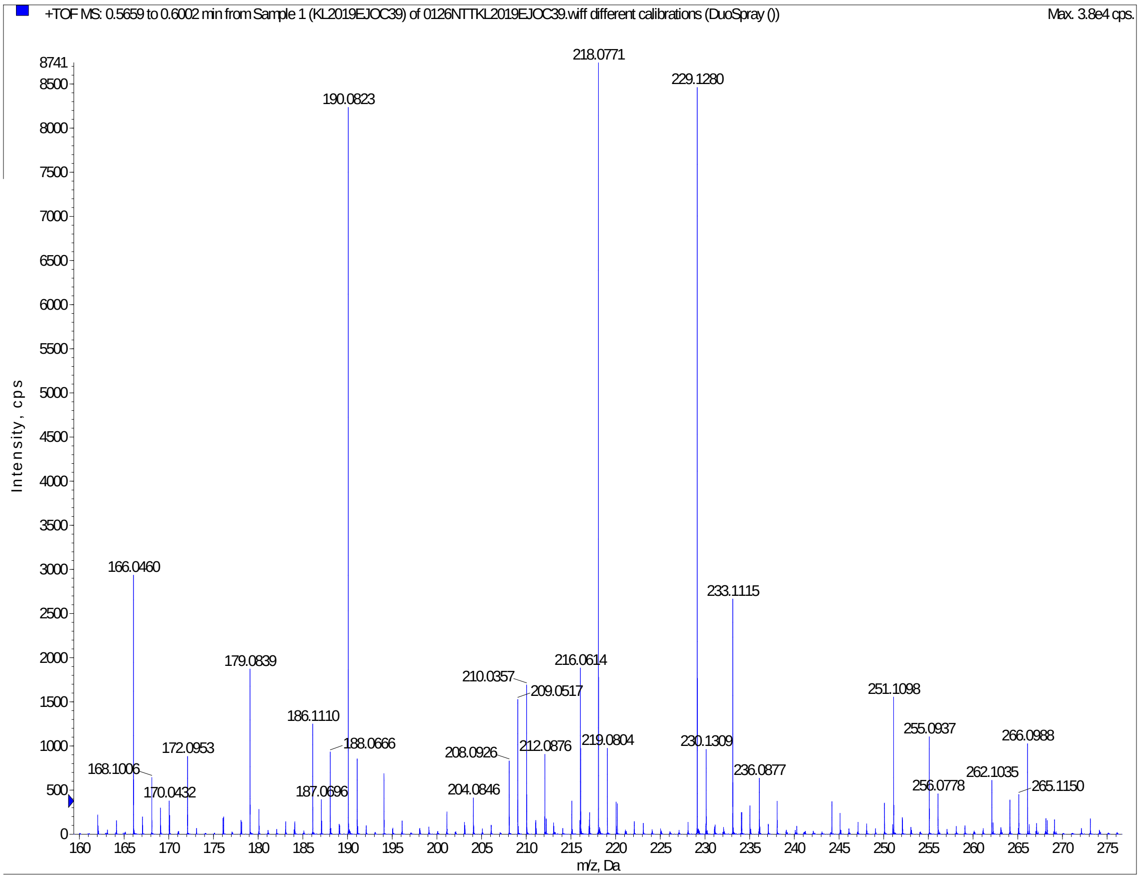

+TOF MS: 0.5659 to 0.6002 min from Sample 1 (KL2019EJOC39) of 0126NTTKL2019EJOC39.wiff different calibrations (DuoSpray ())
Max. 3.8e4 cps.
Intensity, cps
m/z, Da
218.0771
229.1280
190.0823
166.0460
233.1115
179.0839
216.0614
210.0357
209.0517
251.1098
186.1110
188.0666
219.0804
230.1309
255.0937
266.0988
172.0953
208.0926
212.0876
236.0877
168.1006
262.1035
170.0432
187.0696
204.0846
256.0778
265.1150

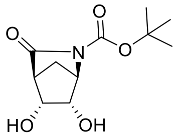

11

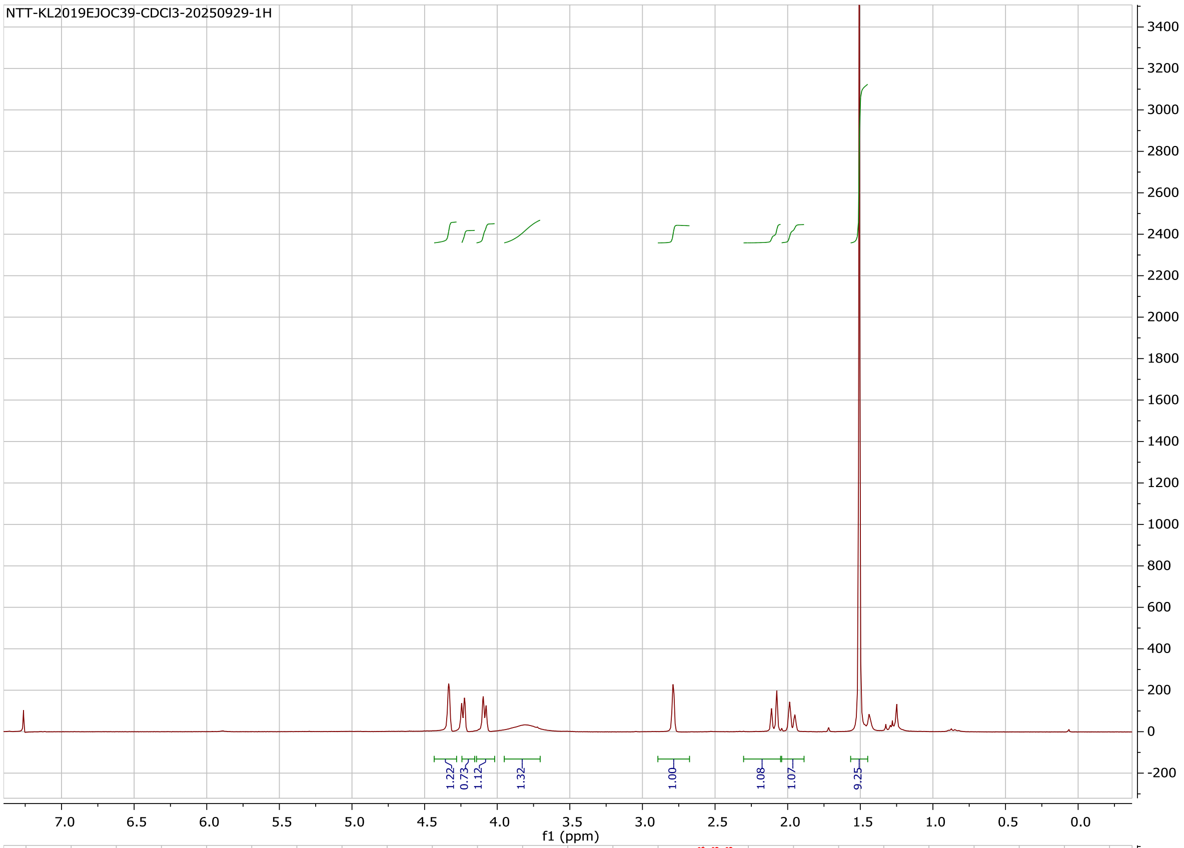
NTT-KL2019EJOC39-CDCl3-20250929-1H
1.22
0.73
1.12
1.32
1.00
1.08
1.07
9.25
f1 (ppm)

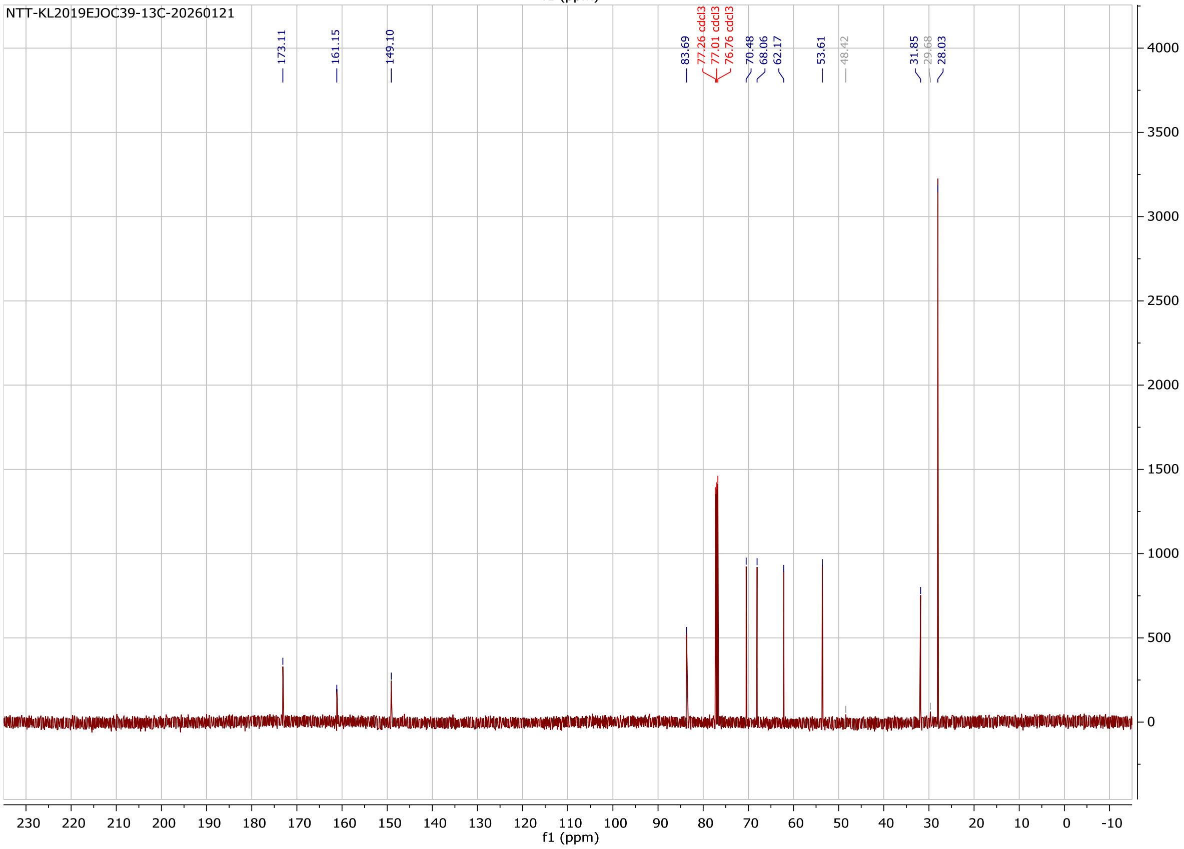
NTT-KL2019EJOC39-13C-20260121
173.11
161.15
149.10
83.69
77.26 cdcl3
77.01 cdcl3
76.76 cdcl3
70.48
68.06
62.17
53.61
48.42
31.85
29.68
28.03
f1 (ppm)

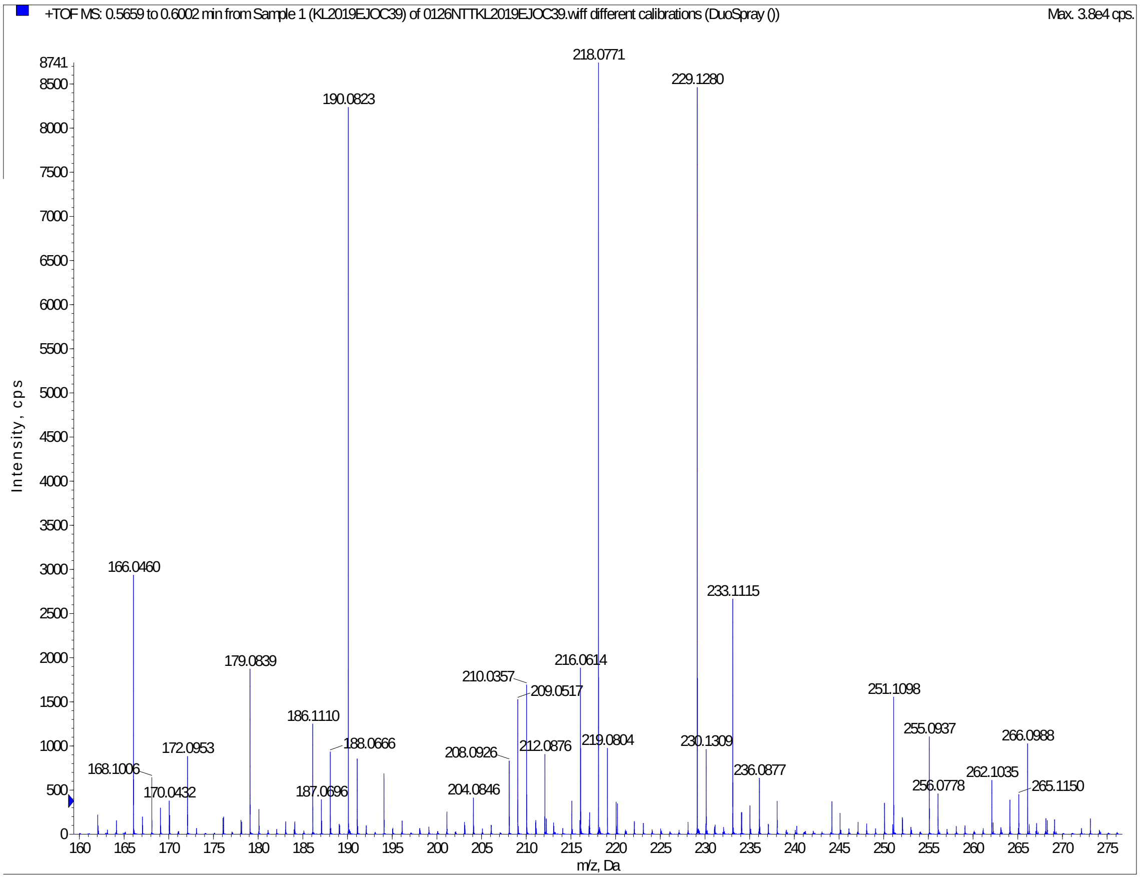
+TOF MS: 0.5659 to 0.6002 min from Sample 1 (KL2019EJOC39) of 0126NTTKL2019EJOC39.wiff different calibrations (DuoSpray ())
Max. 3.8e4 cps.
Intensity, cps
m/z, Da
218.0771
229.1280
190.0823
166.0460
233.1115
179.0839
216.0614
210.0357
209.0517
251.1098
186.1110
188.0666
172.0953
208.0926
212.0876
219.0804
230.1309
255.0937
266.0988
168.1006
236.0877
262.1035
170.0432
187.0696
204.0846
256.0778
265.1150

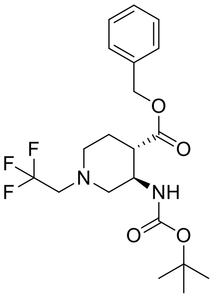

13

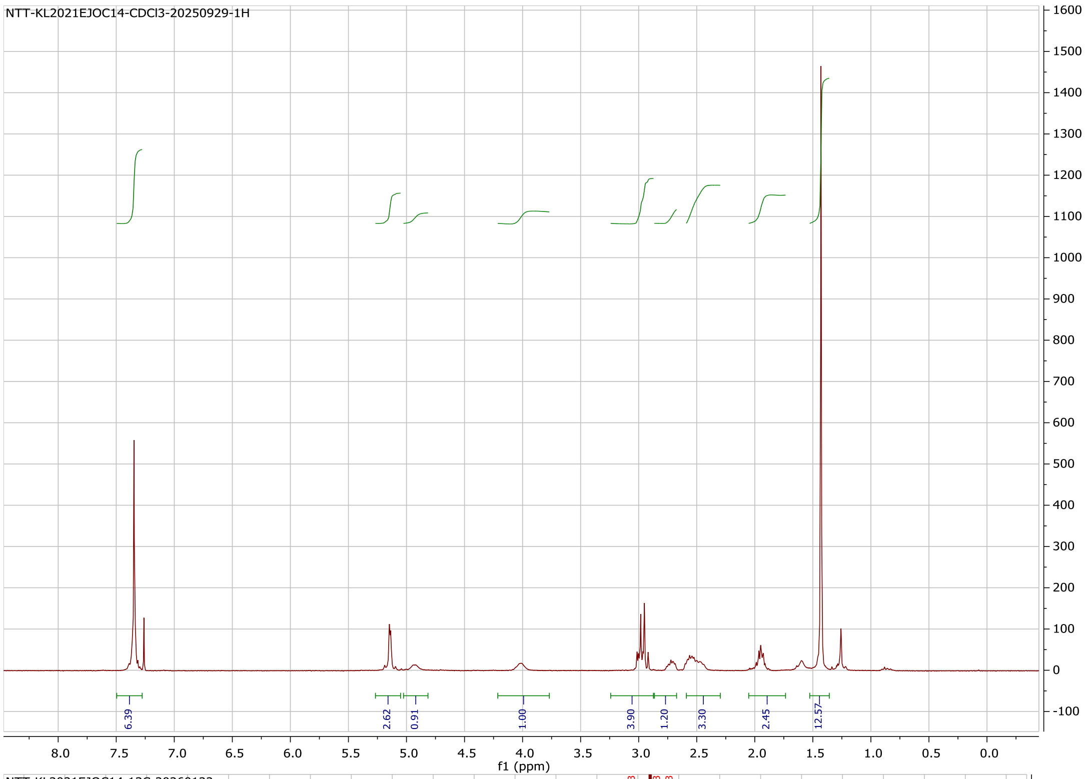
NTT-KL2021EJOC14-CDCl3-20250929-1H
6.39
2.62
0.91
1.00
3.90
1.20
3.30
2.45
12.57
f1 (ppm)

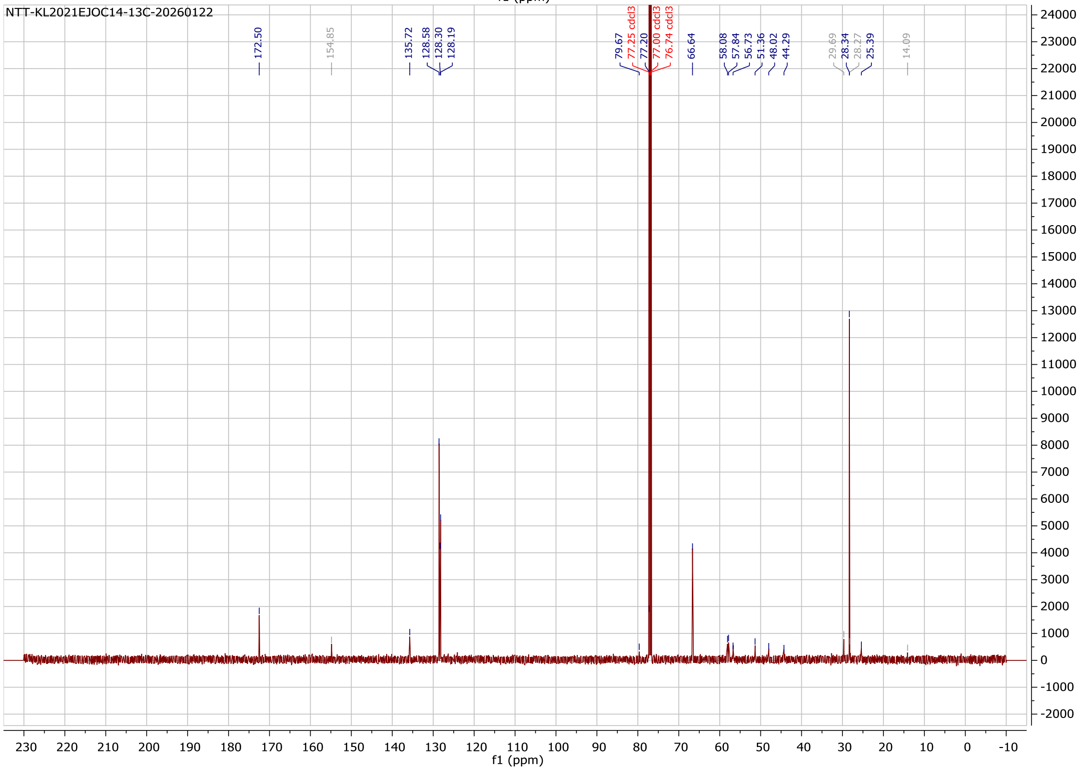
NTT-KL2021EJOC14-13C-20260122
172.50
154.85
135.72
128.58
128.30
128.19
79.67
77.25 cdcl3
77.20
77.00 cdcl3
76.74 cdcl3
66.64
58.08
57.84
56.73
51.36
48.02
44.29
29.69
28.34
28.27
25.39
14.09
f1 (ppm)

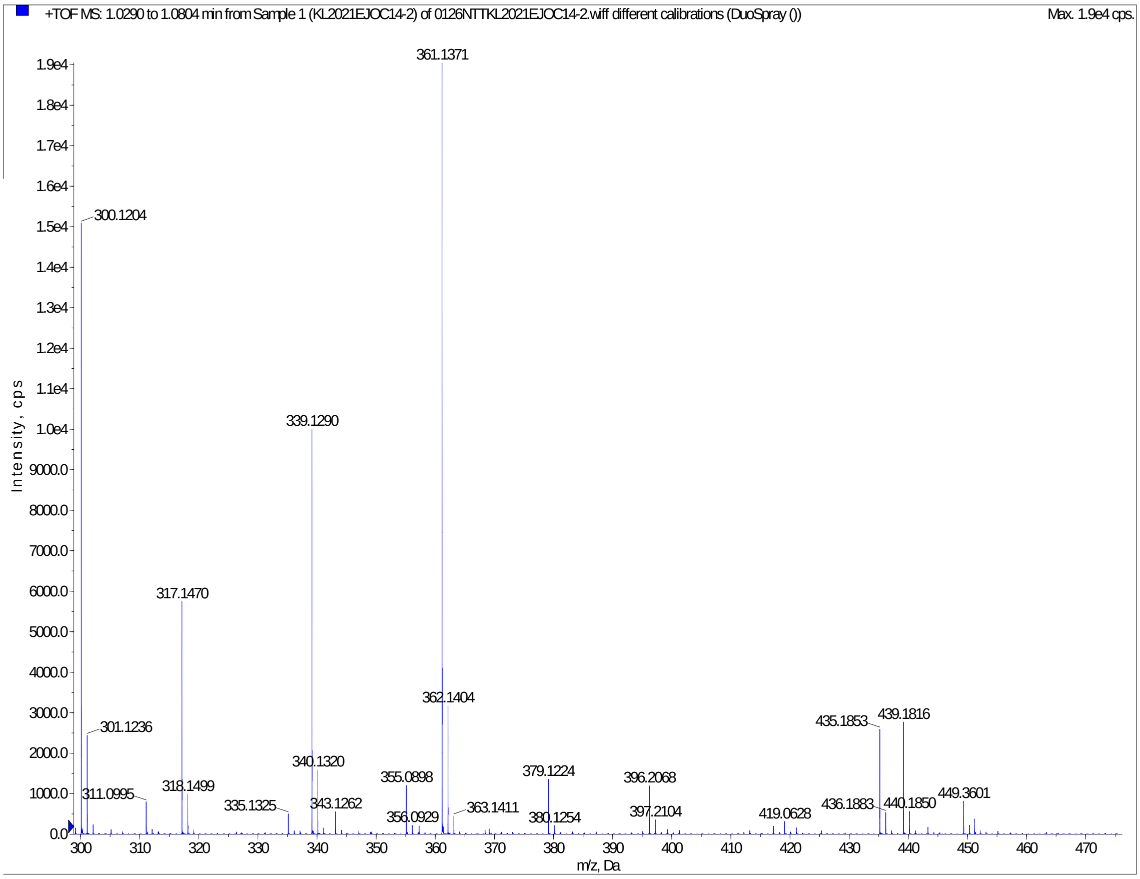
+TOF MS: 1.0290 to 1.0804 min from Sample 1 (KL2021EJOC14-2) of 0126NTTKL2021EJOC14-2.wiff different calibrations (DuoSpray ())
Max. 1.9e4 cps.
Intensity, cps
m/z, Da
361.1371
300.1204
339.1290
317.1470
362.1404
301.1236
435.1853
439.1816
340.1320
355.0898
379.1224
396.2068
318.1499
311.0995
335.1325
343.1262
449.3601
436.1883
440.1850
363.1411
397.2104
419.0628
356.0929
380.1254

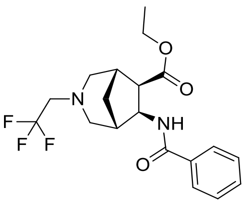

15

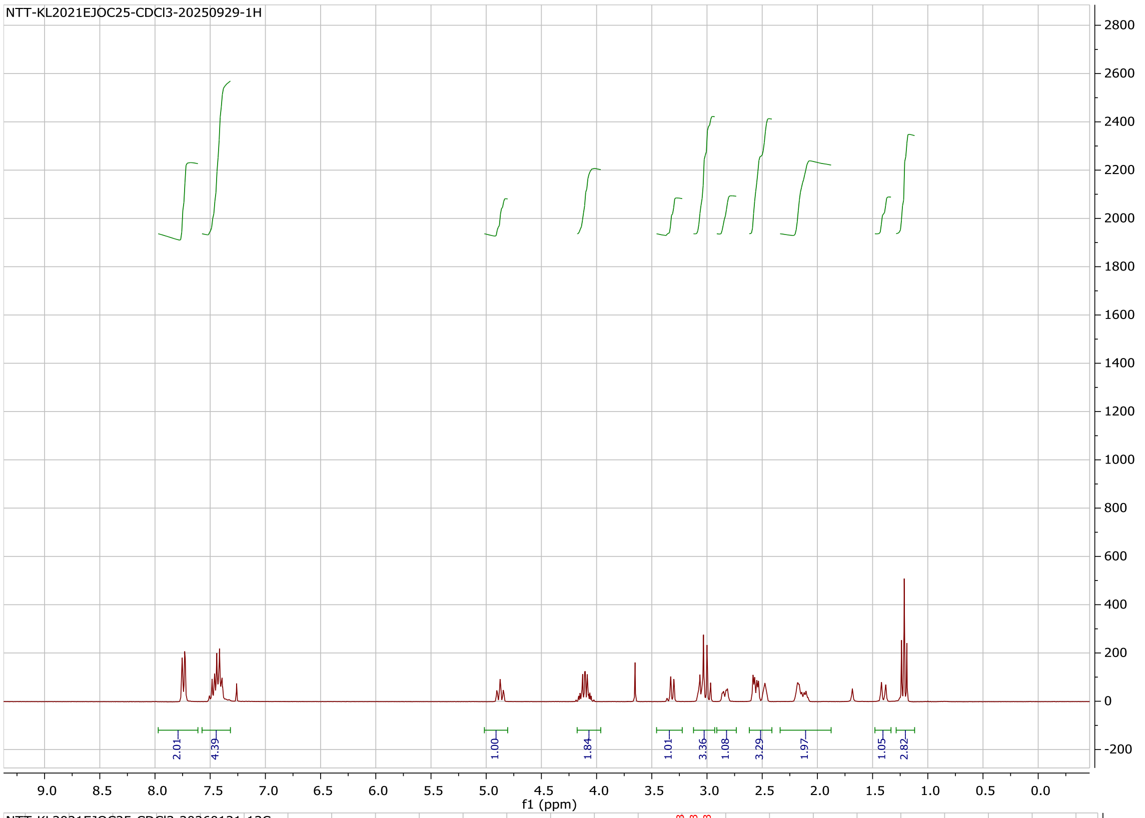

NTT-KL2021EJOC25-CDCl3-20250929-1H
2.01
4.39
1.00
1.84
1.01
3.36
1.08
3.29
1.97
1.05
2.82
f1 (ppm)

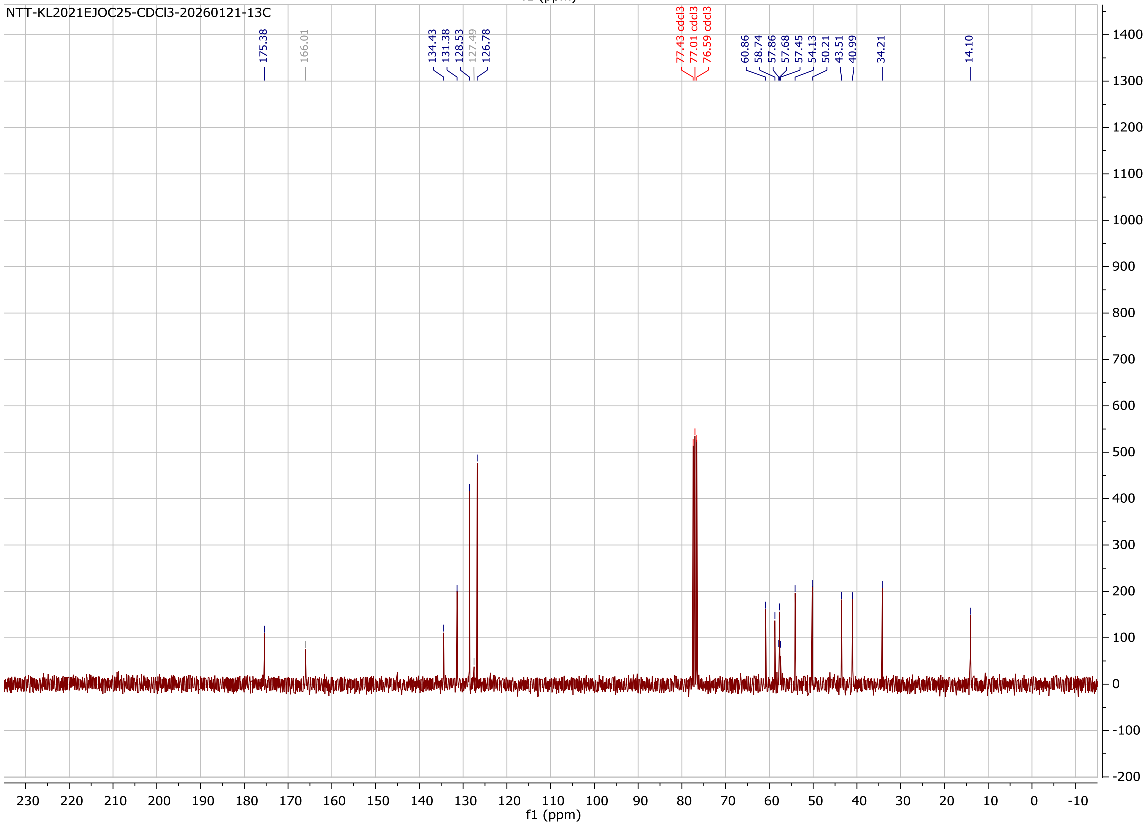

NTT-KL2021EJOC25-CDCl3-20260121-13C
175.38
166.01
134.43
131.38
128.53
127.49
126.78
77.43 cdcl3
77.01 cdcl3
76.59 cdcl3
60.86
58.74
57.86
57.68
57.45
54.13
50.21
43.51
40.99
34.21
14.10
f1 (ppm)

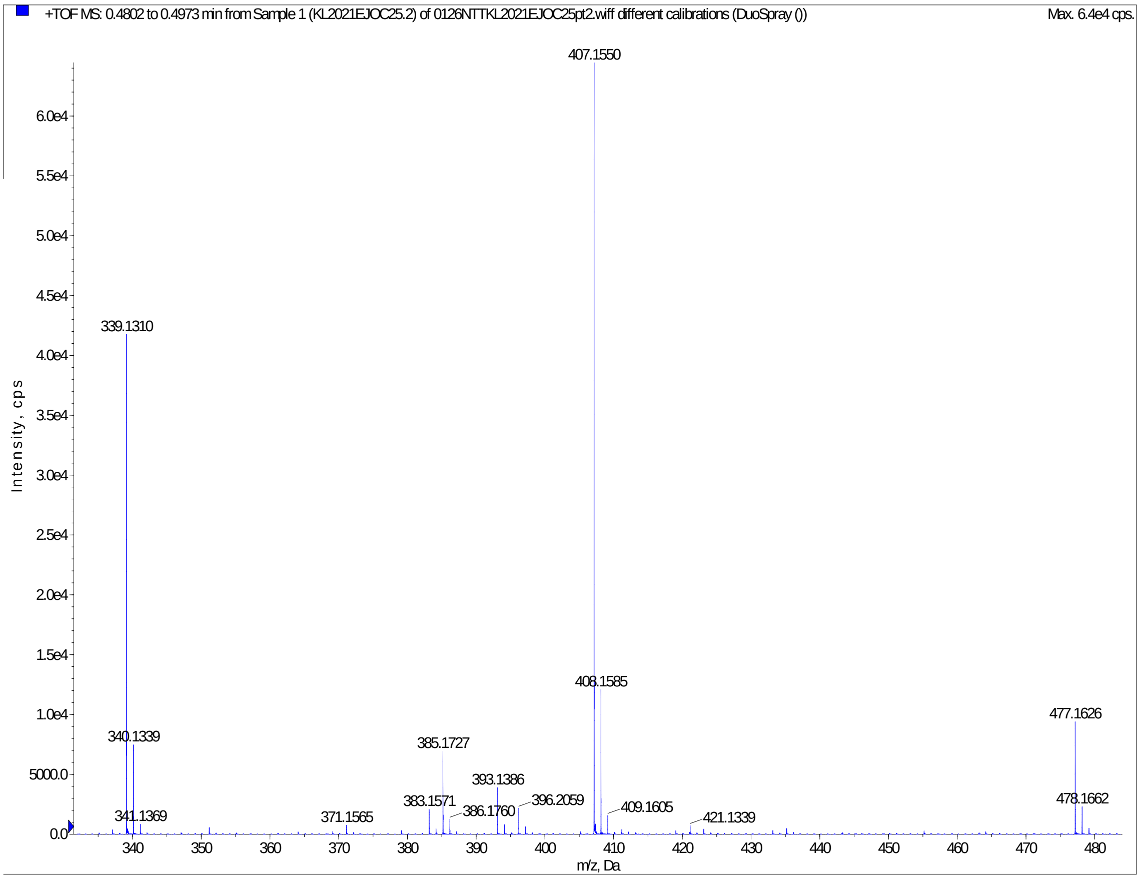
+TOF MS: 0.4802 to 0.4973 min from Sample 1 (KL2021EJOC25.2) of 0126NTTKL2021EJOC25pt2.wiff different calibrations (DuoSpray ())
Max. 6.4e4 cps.
Intensity, cps
m/z, Da
407.1550
339.1310
408.1585
477.1626
340.1339
385.1727
393.1386
383.1571
396.2059
478.1662
386.1760
409.1605
341.1369
371.1565
421.1339

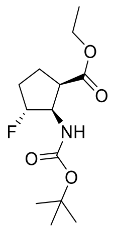

16

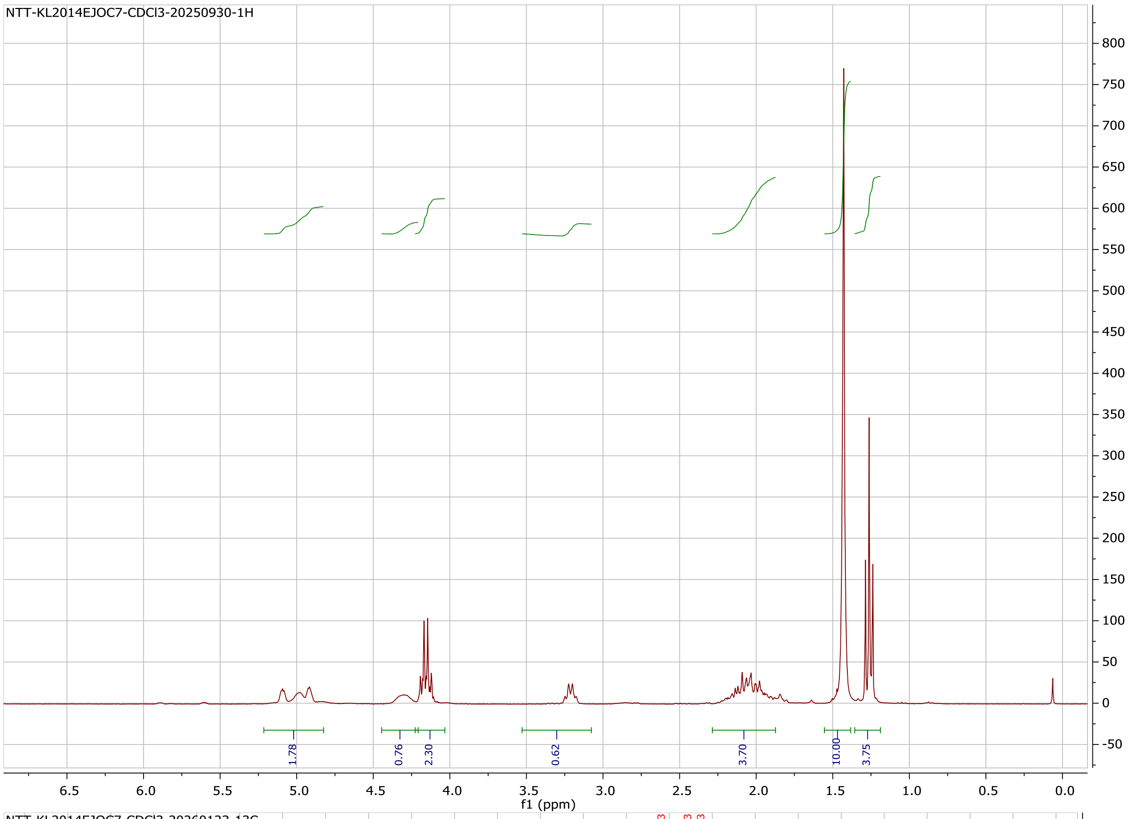

NTT-KL2014EJOC7-CDCl3-20250930-1H
1.78
0.76
2.30
0.62
3.70
10.00
3.75
f1 (ppm)

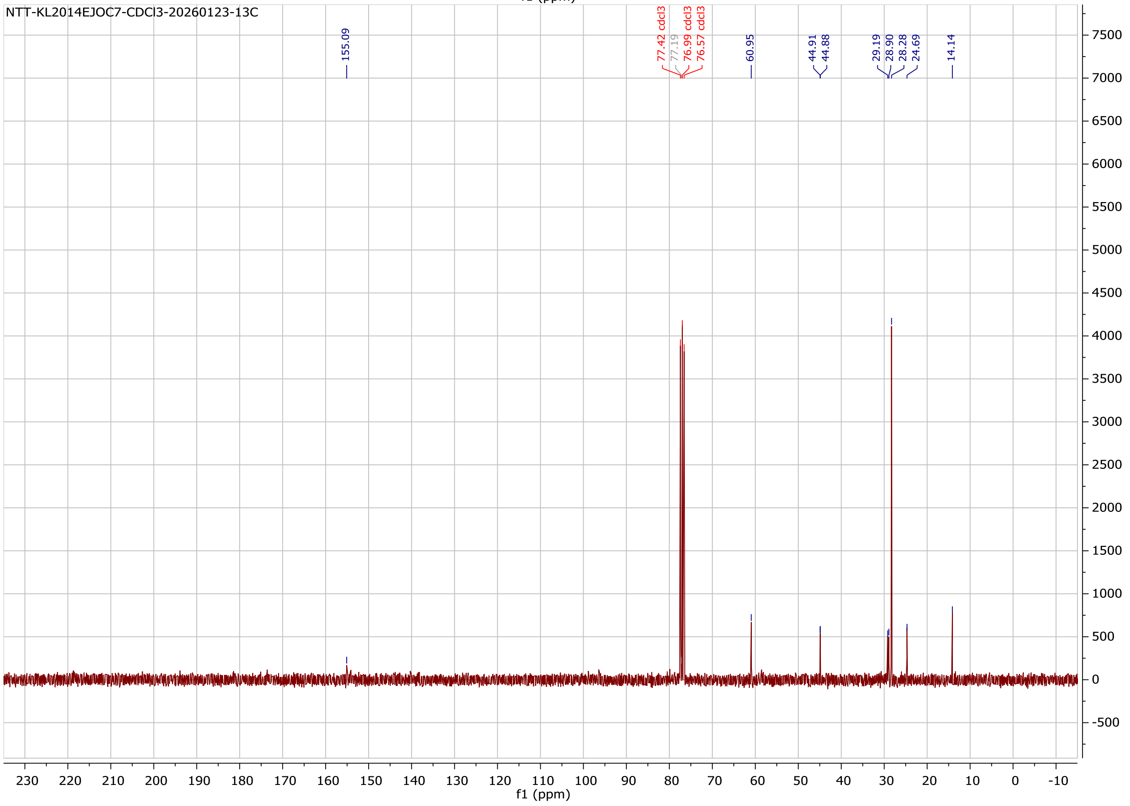

NTT-KL2014EJOC7-CDCl3-20260123-13C
155.09
77.42 cdcl3
77.19
76.99 cdcl3
76.57 cdcl3
60.95
44.91
44.88
29.19
28.90
28.28
24.69
14.14
f1 (ppm)

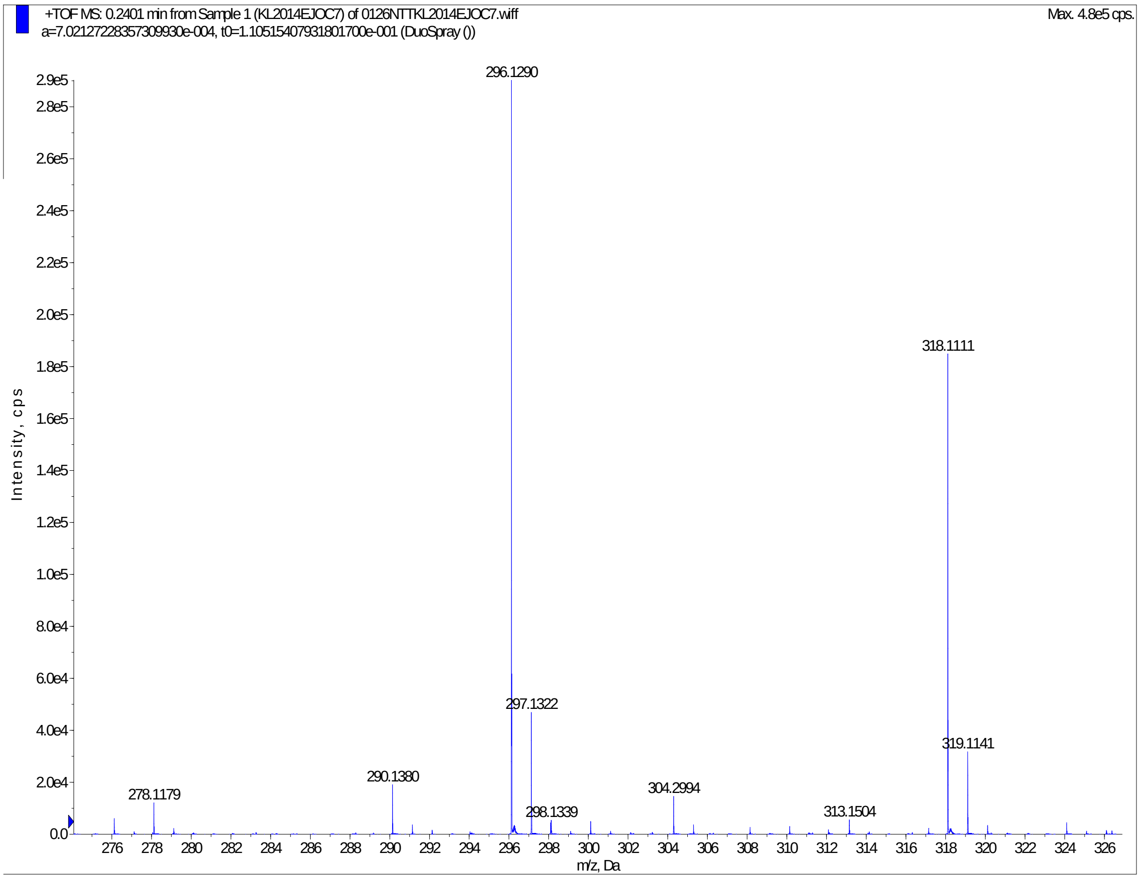

+TOF MS: 0.2401 min from Sample 1 (KL2014EJOC7) of 0126NTTKL2014EJOC7.wiff
a=7.02127228357309930e-004, t0=1.10515407931801700e-001 (DuoSpray ())
Max. 4.8e5 cps.
Intensity, cps
m/z, Da
296.1290
318.1111
297.1322
319.1141
290.1380
304.2994
278.1179
298.1339
313.1504

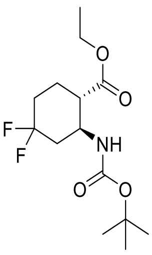

O
O
F
F
NH
O
O

18

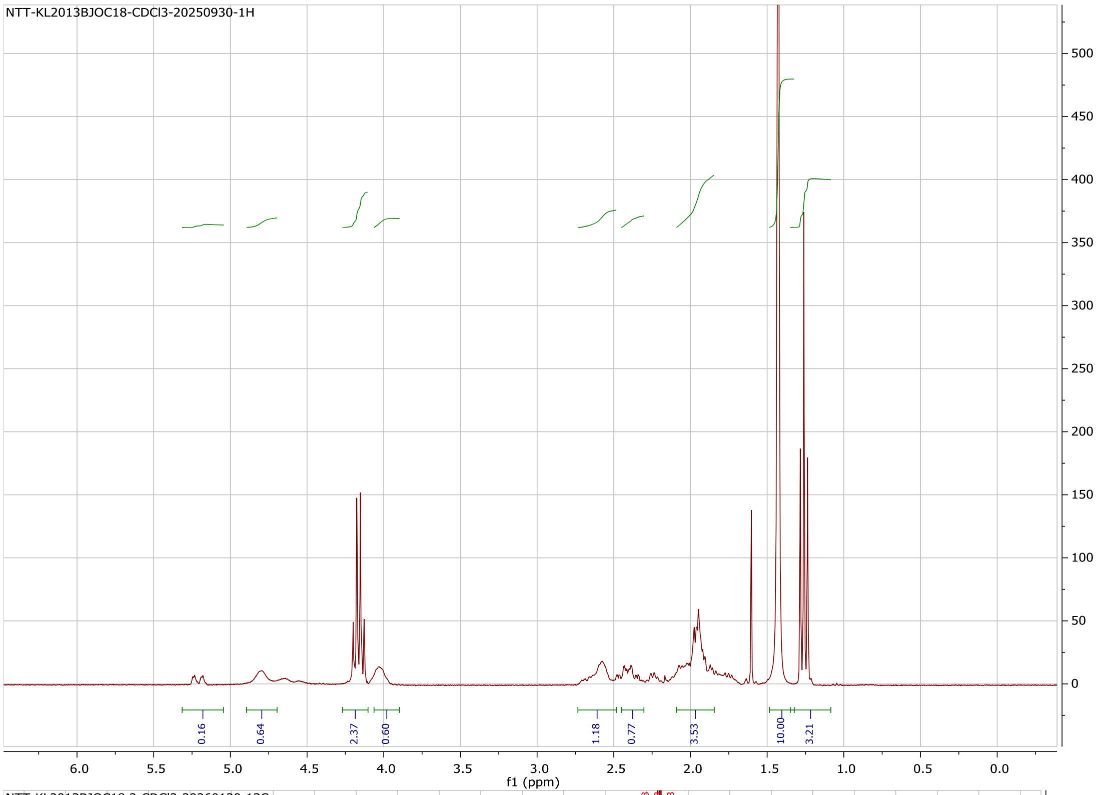

NTT-KL2013BJOC18-CDCl3-20250930-1H
0.16
0.64
2.37
0.60
1.18
0.77
3.53
10.00
3.21
f1 (ppm)

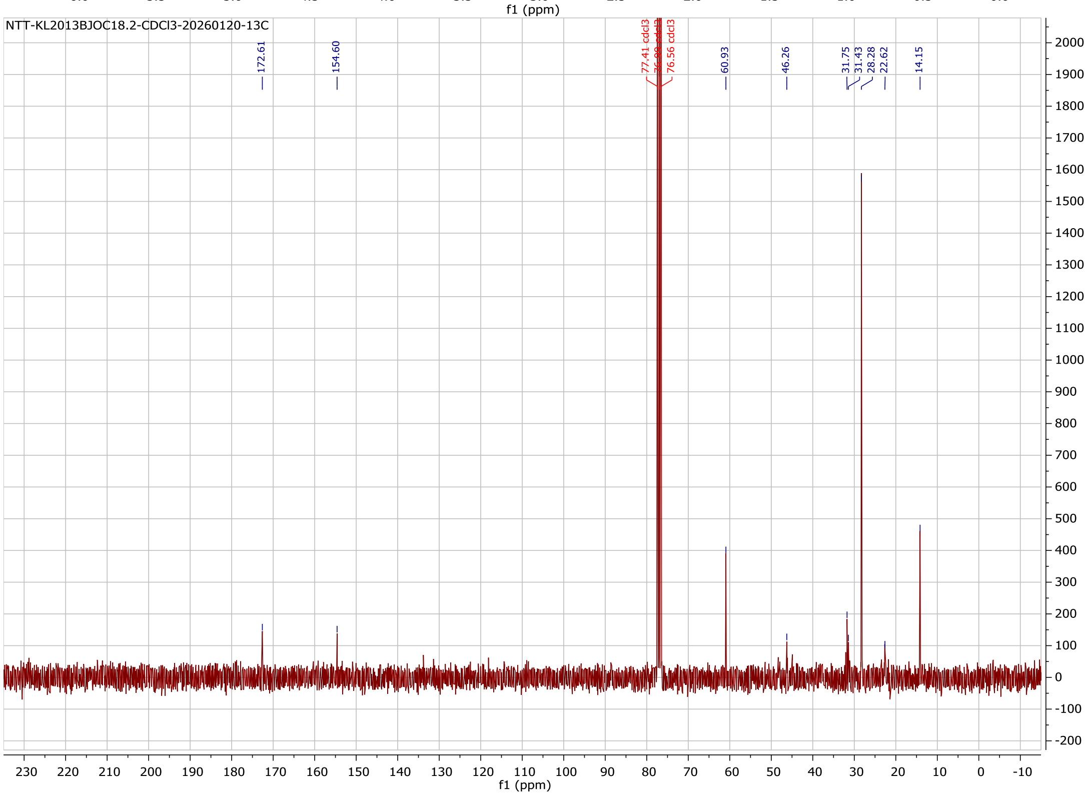

NTT-KL2013BJOC18.2-CDCl3-20260120-13C
172.61
154.60
77.41 cdcl3
76.56 cdcl3
60.93
46.26
31.75
31.43
28.28
22.62
14.15
f1 (ppm)

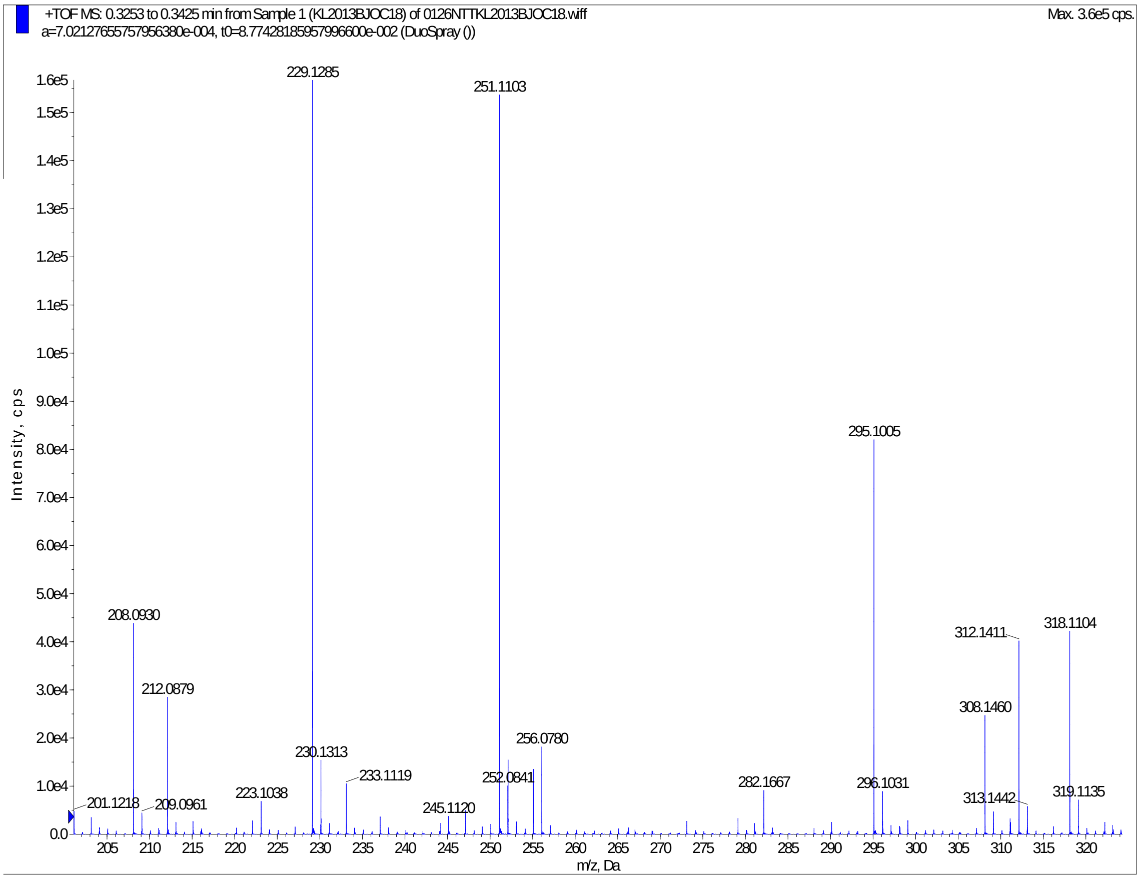
+TOF MS: 0.3253 to 0.3425 min from Sample 1 (KL2013BJOC18) of 0126NTTKL2013BJOC18.wiff
a=7.02127655757956380e-004, t0=8.77428185957996600e-002 (DuoSpray ())
Max. 3.6e5 cps.
Intensity, cps
m/z, Da
229.1285
251.1103
295.1005
208.0930
212.0879
312.1411
318.1104
308.1460
256.0780
230.1313
233.1119
252.0841
282.1667
296.1031
201.1218
209.0961
223.1038
245.1120
313.1442
319.1135

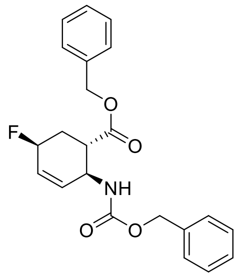

20

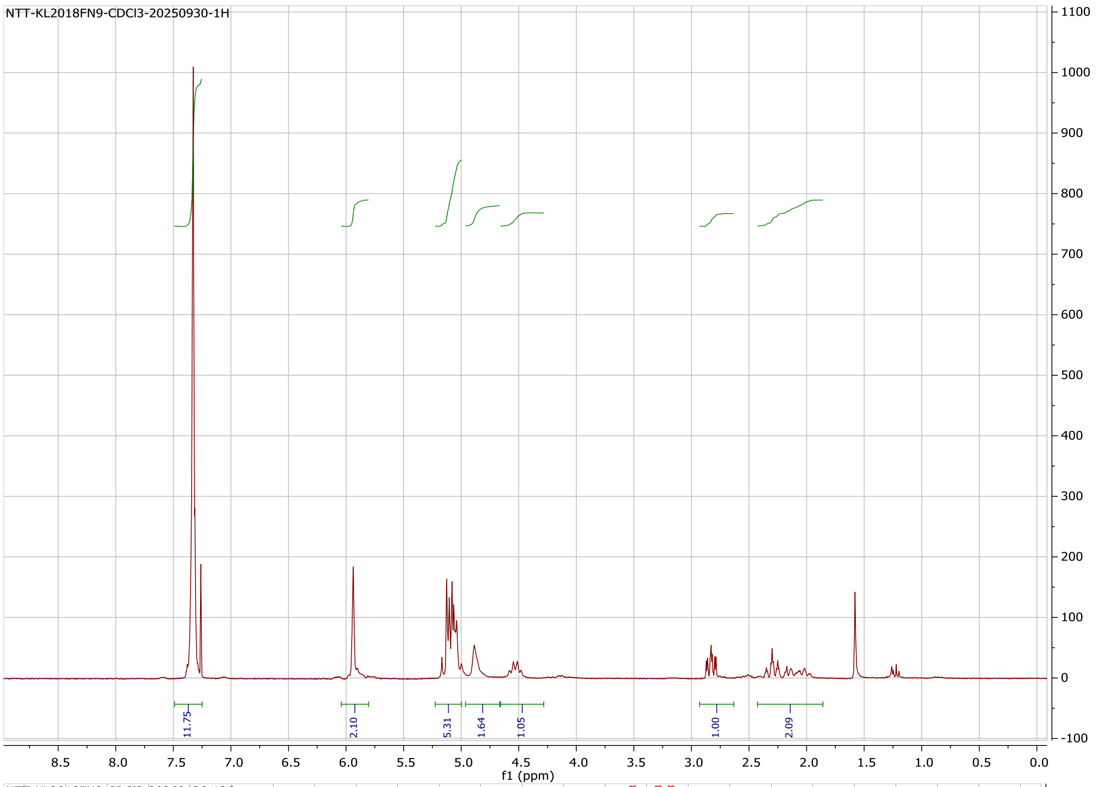

NTT-KL2018FN9-CDCl3-20250930-1H
11.75
2.10
5.31
1.64
1.05
1.00
2.09
f1 (ppm)

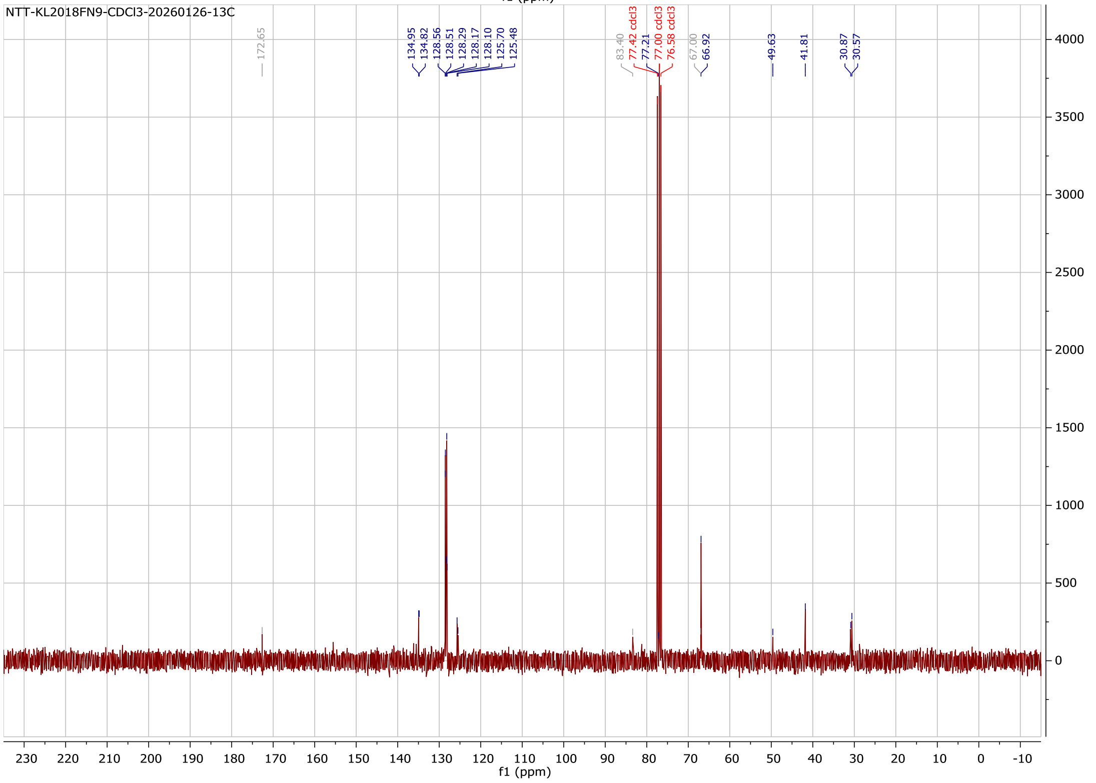

NTT-KL2018FN9-CDCl3-20260126-13C
172.65
134.95
134.82
128.56
128.51
128.29
128.17
128.10
125.70
125.48
83.40
77.42 cdcl3
77.21
77.00 cdcl3
76.58 cdcl3
67.00
66.92
49.63
41.81
30.87
30.57
f1 (ppm)

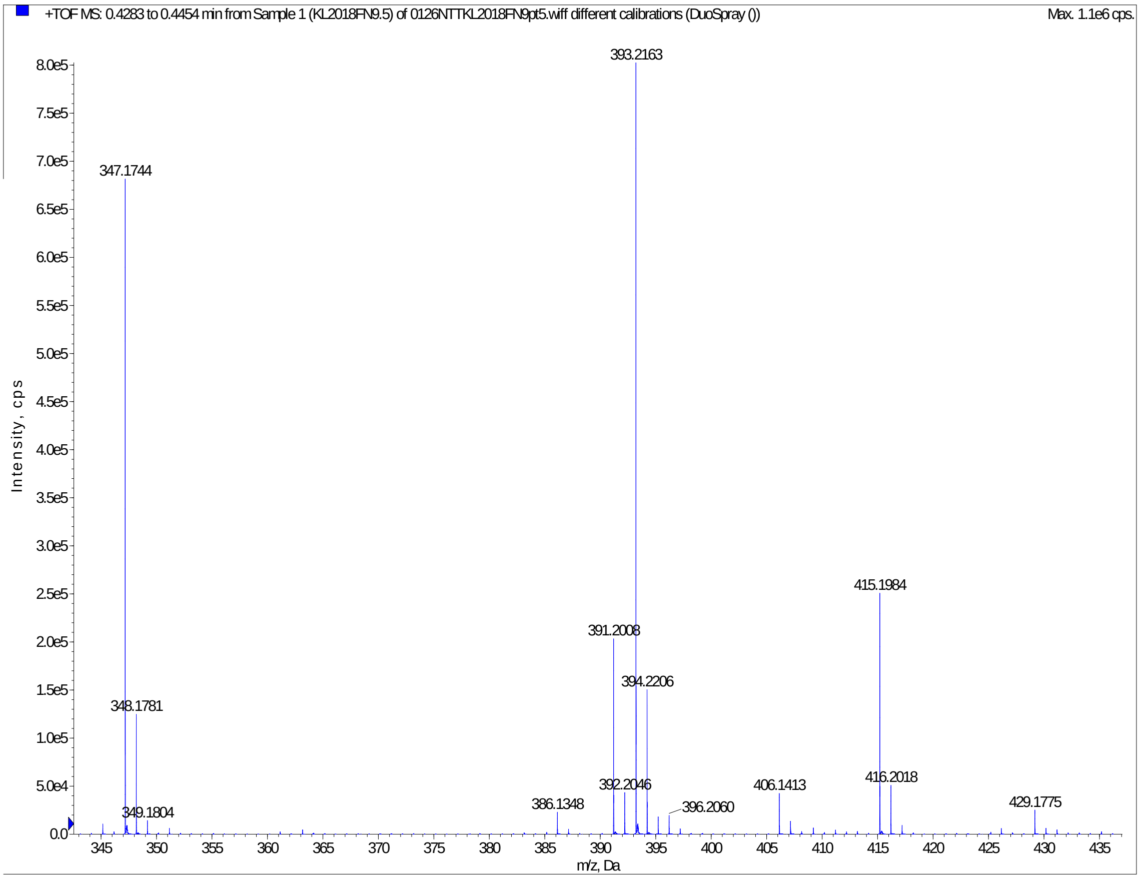
+TOF MS: 0.4283 to 0.4454 min from Sample 1 (KL2018FN9.5) of 0126NTTKL2018FN9pt5.wiff different calibrations (DuoSpray ())
Max. 1.1e6 cps.
Intensity, cps
m/z, Da
393.2163
347.1744
415.1984
391.2008
394.2206
348.1781
392.2046
406.1413
416.2018
349.1804
386.1348
396.2060
429.1775

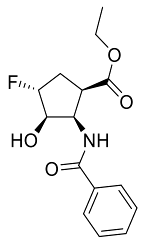

17

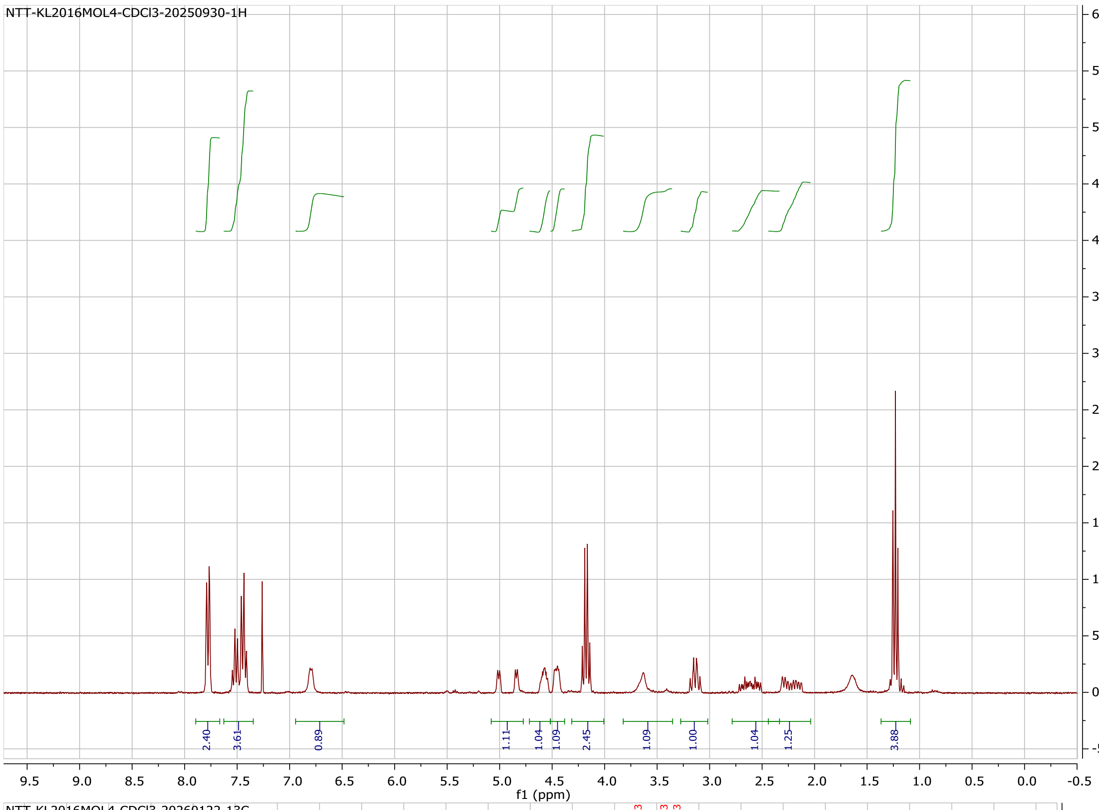
NTT-KL2016MOL4-CDCl3-20250930-1H
2.40
3.61
0.89
1.11
1.04
1.09
2.45
1.09
1.00
1.04
1.25
3.88
f1 (ppm)

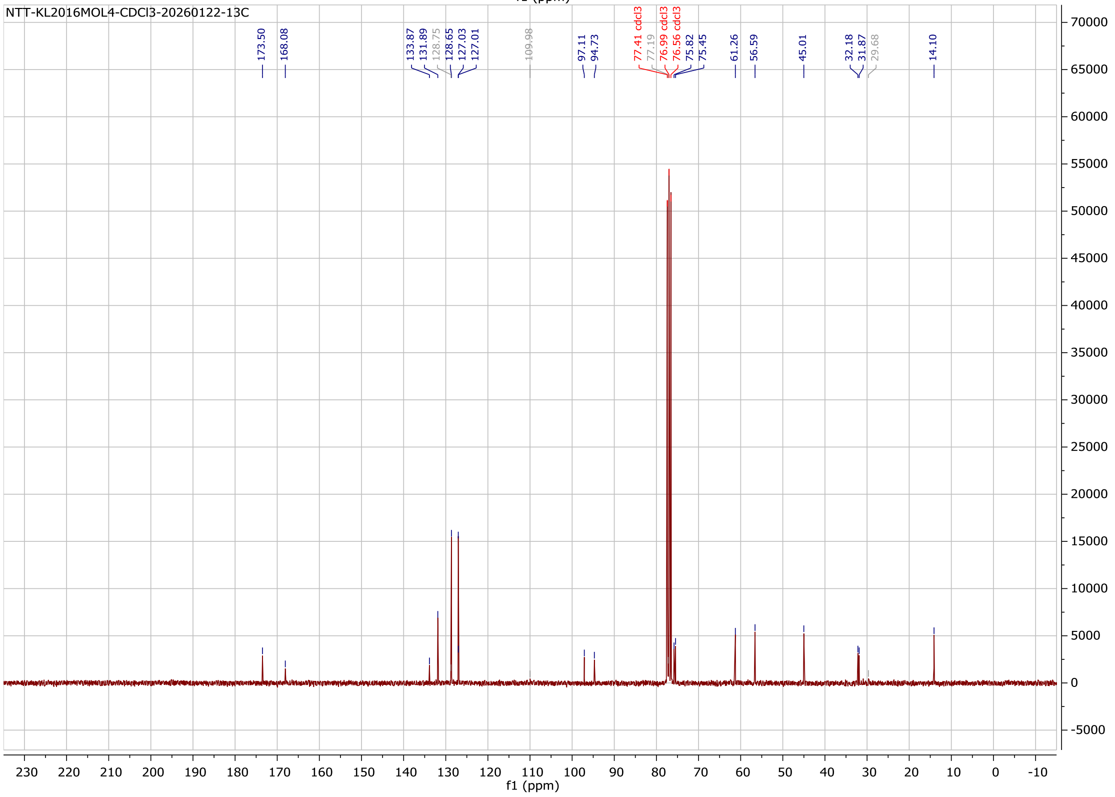
NTT-KL2016MOL4-CDCl3-20260122-13C
173.50
168.08
133.87
131.89
128.75
128.65
127.03
127.01
109.98
97.11
94.73
77.41 cdcl3
77.19
76.99 cdcl3
76.56 cdcl3
75.82
75.45
61.26
56.59
45.01
32.18
31.87
29.68
14.10
f1 (ppm)

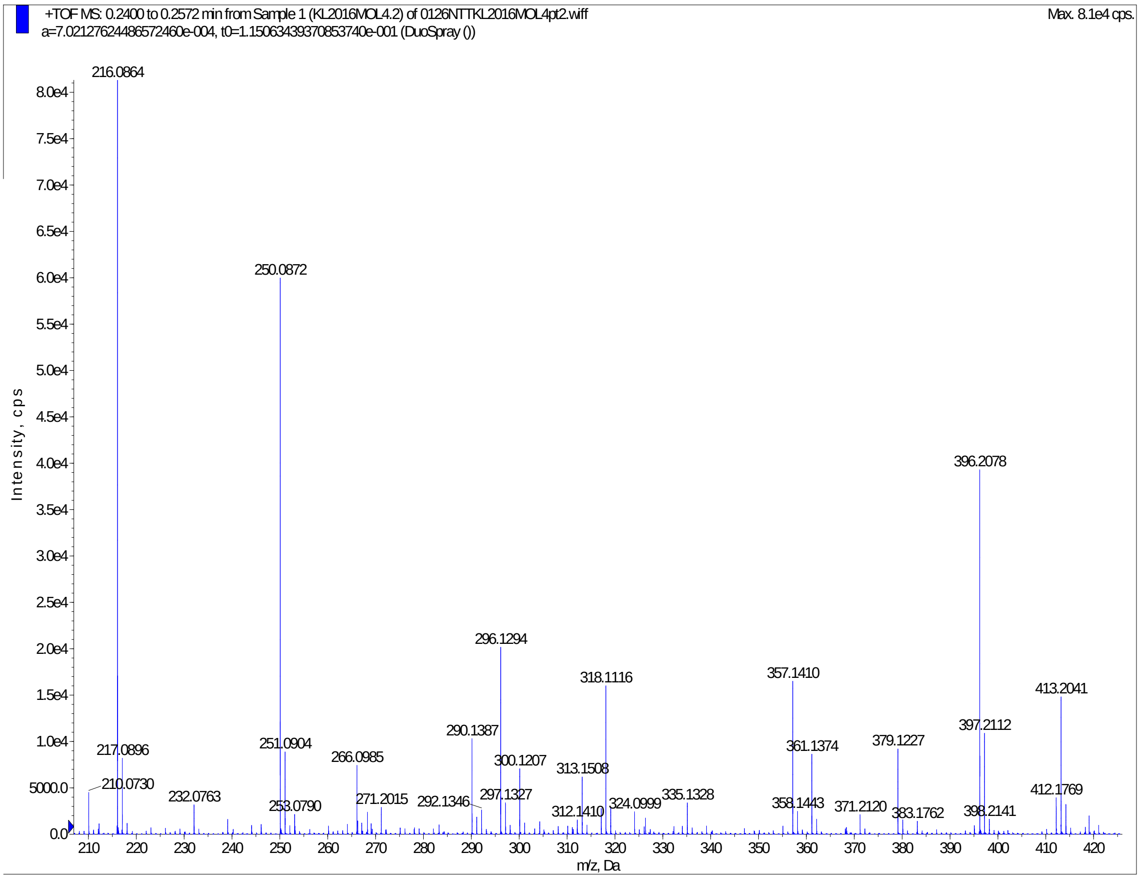

+TOF MS: 0.2400 to 0.2572 min from Sample 1 (KL2016MOL4.2) of 0126NTTKL2016MOL4pt2.wiff
a=7.02127624486572460e-004, t0=1.15063439370853740e-001 (DuoSpray ())
Max. 8.1e4 cps.
Intensity, cps
m/z, Da
216.0864
250.0872
396.2078
296.1294
318.1116
357.1410
413.2041
290.1387
397.2112
251.0904
379.1227
217.0896
361.1374
300.1207
266.0985
313.1508
210.0730
232.0763
335.1328
297.1327
412.1769
271.2015
292.1346
358.1443
324.0999
253.0790
371.2120
312.1410
398.2141
383.1762

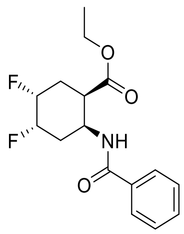
F
F
O
O
NH
O

19

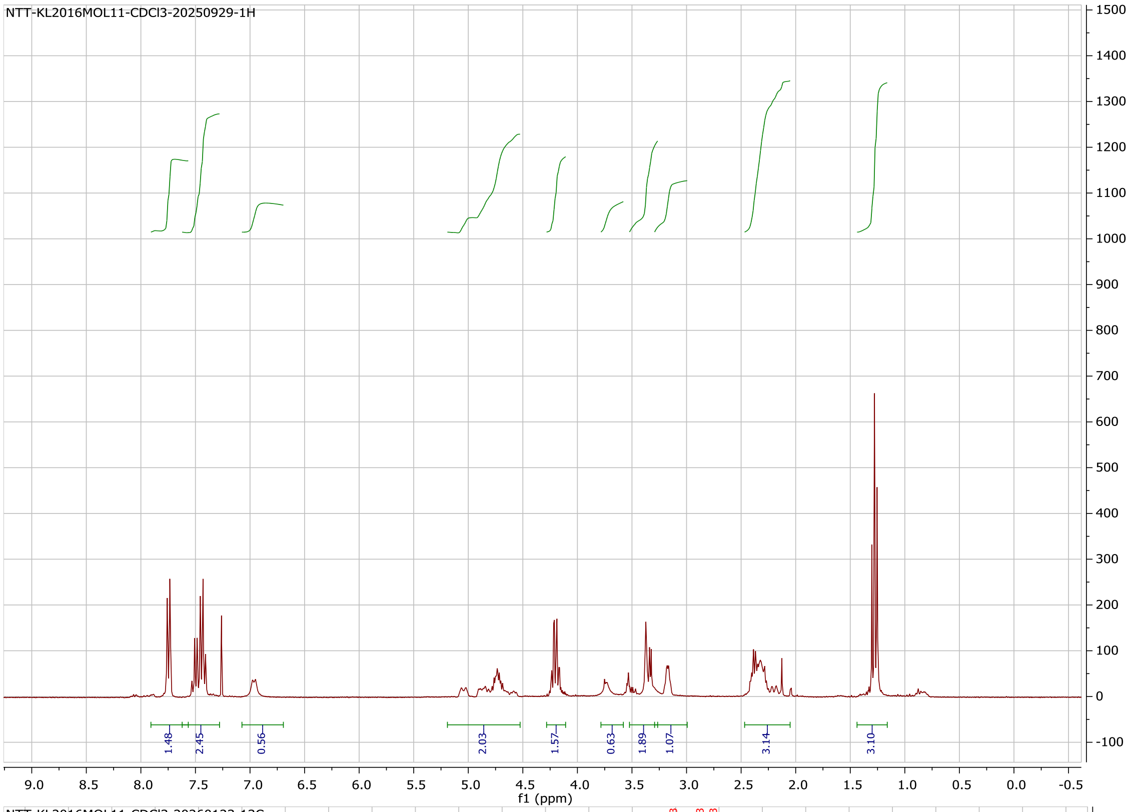
NTT-KL2016MOL11-CDCl3-20250929-1H
1.48
2.45
0.56
2.03
1.57
0.63
1.89
1.07
3.14
3.10
f1 (ppm)

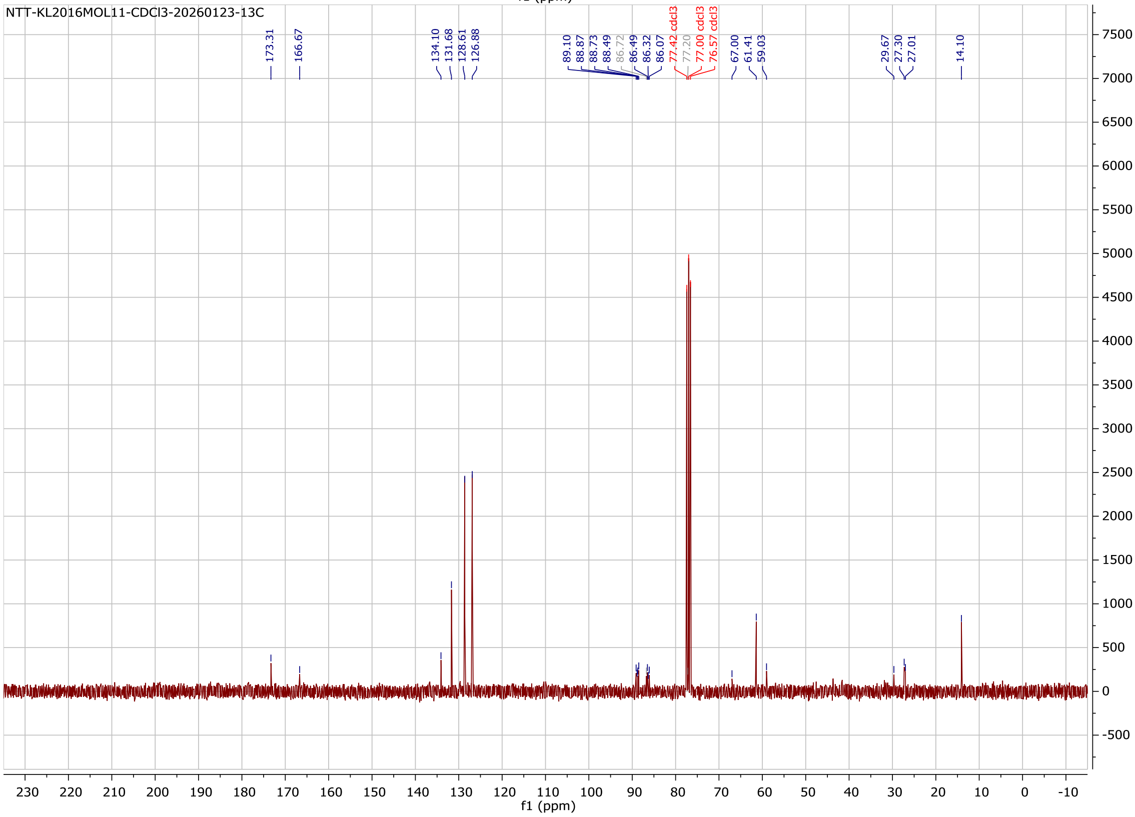
NTT-KL2016MOL11-CDCl3-20260123-13C
173.31
166.67
134.10
131.68
128.61
126.88
89.10
88.87
88.73
88.49
86.72
86.49
86.32
86.07
77.42 cdcl3
77.20
77.00 cdcl3
76.57 cdcl3
67.00
61.41
59.03
29.67
27.30
27.01
14.10
f1 (ppm)

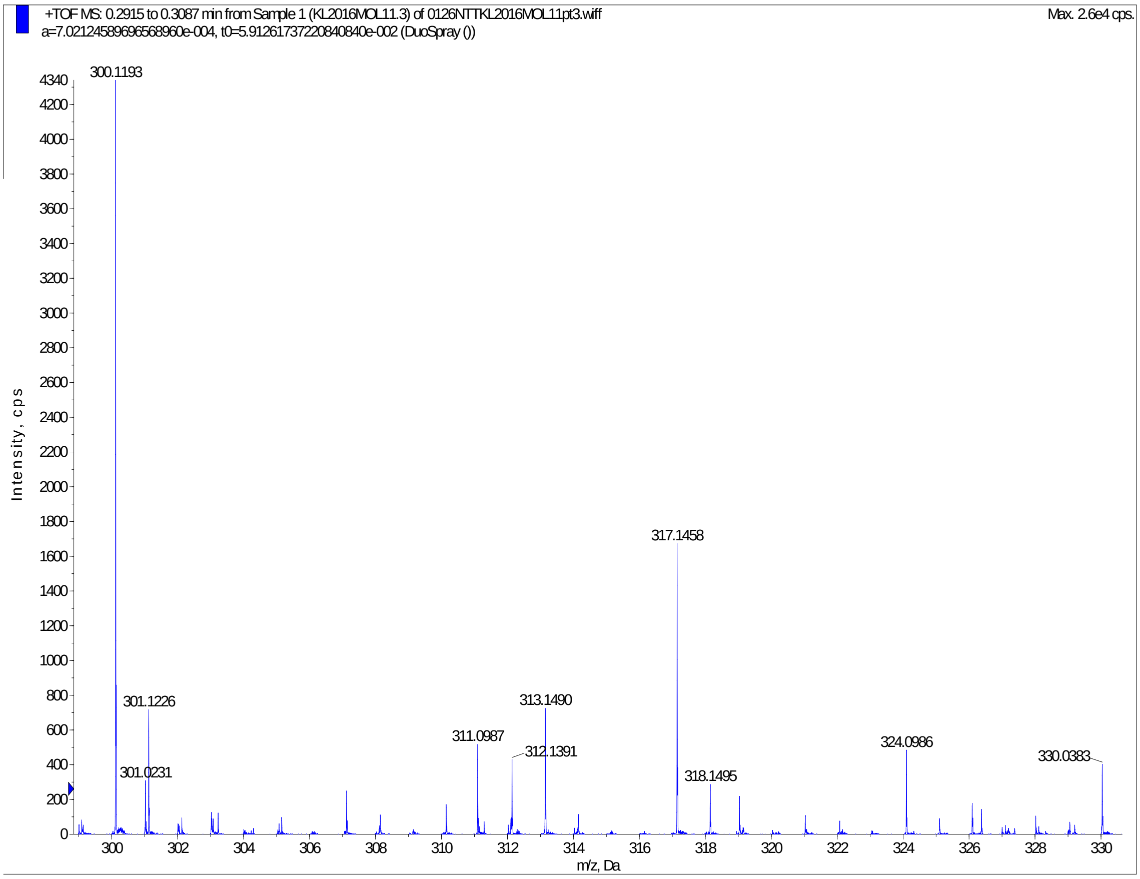
+TOF MS: 0.2915 to 0.3087 min from Sample 1 (KL2016MOL11.3) of 0126NTTKL2016MOL11pt3.wiff
a=7.02124589696568960e-004, t0=5.91261737220840840e-002 (DuoSpray ())
Max. 2.6e4 cps.
Intensity, cps
m/z, Da
300.1193
301.1226
301.0231
311.0987
312.1391
313.1490
317.1458
318.1495
324.0986
330.0383

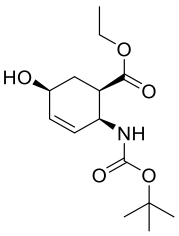

21

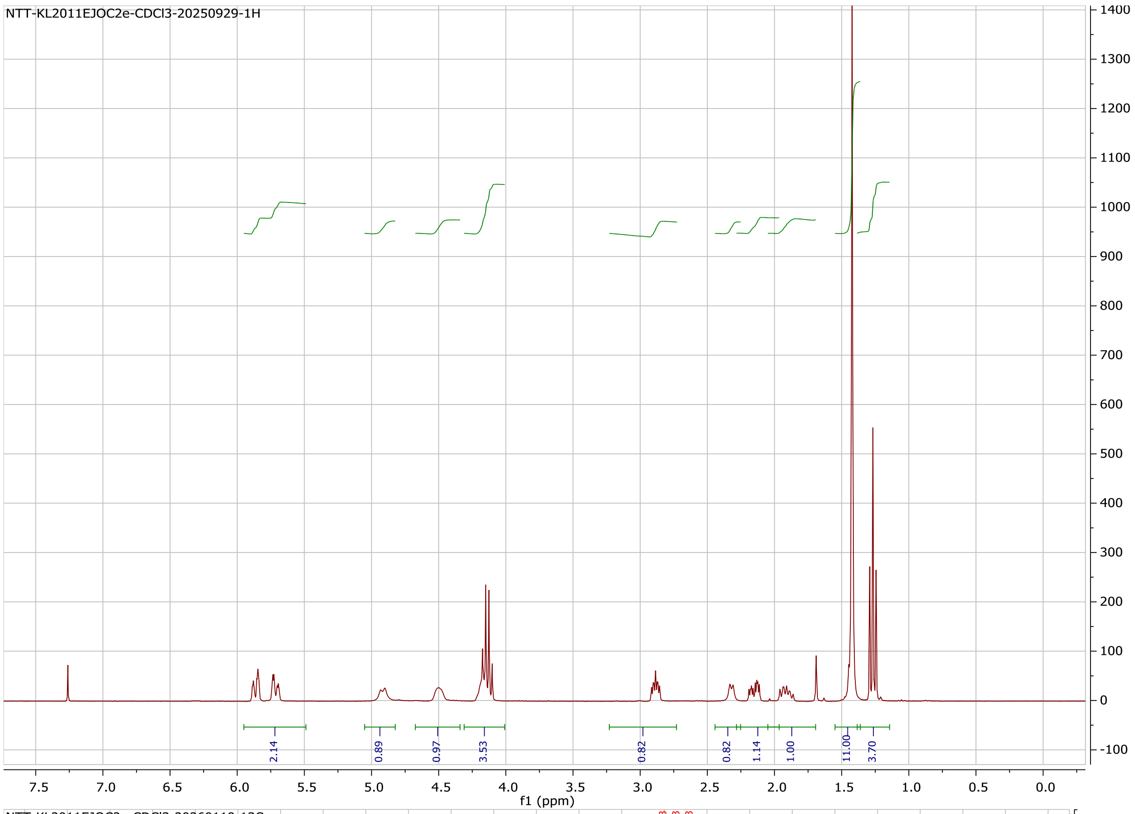

NTT-KL2011EJOC2e-CDCl3-20250929-1H
2.14
0.89
0.97
3.53
0.82
0.82
1.14
1.00
11.00
3.70
f1 (ppm)

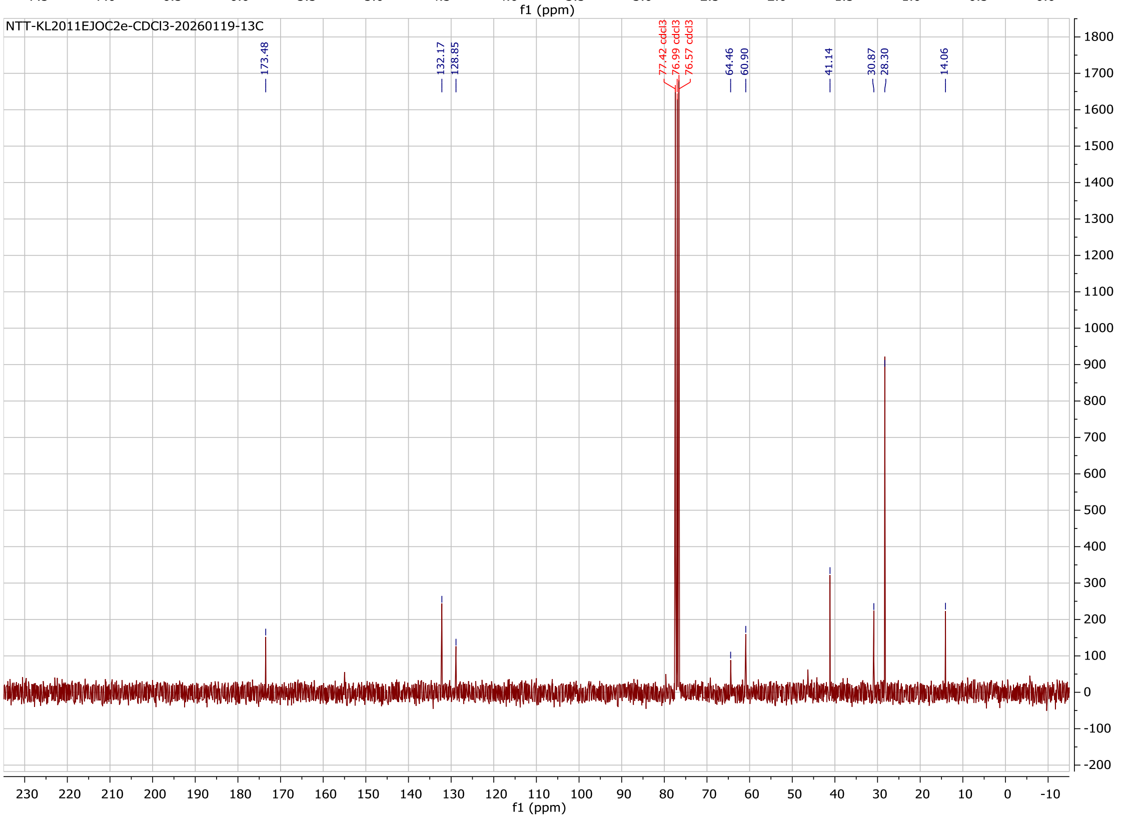

NTT-KL2011EJOC2e-CDCl3-20260119-13C
173.48
132.17
128.85
77.42 cdcl3
76.99 cdcl3
76.57 cdcl3
64.46
60.90
41.14
30.87
28.30
14.06
f1 (ppm)

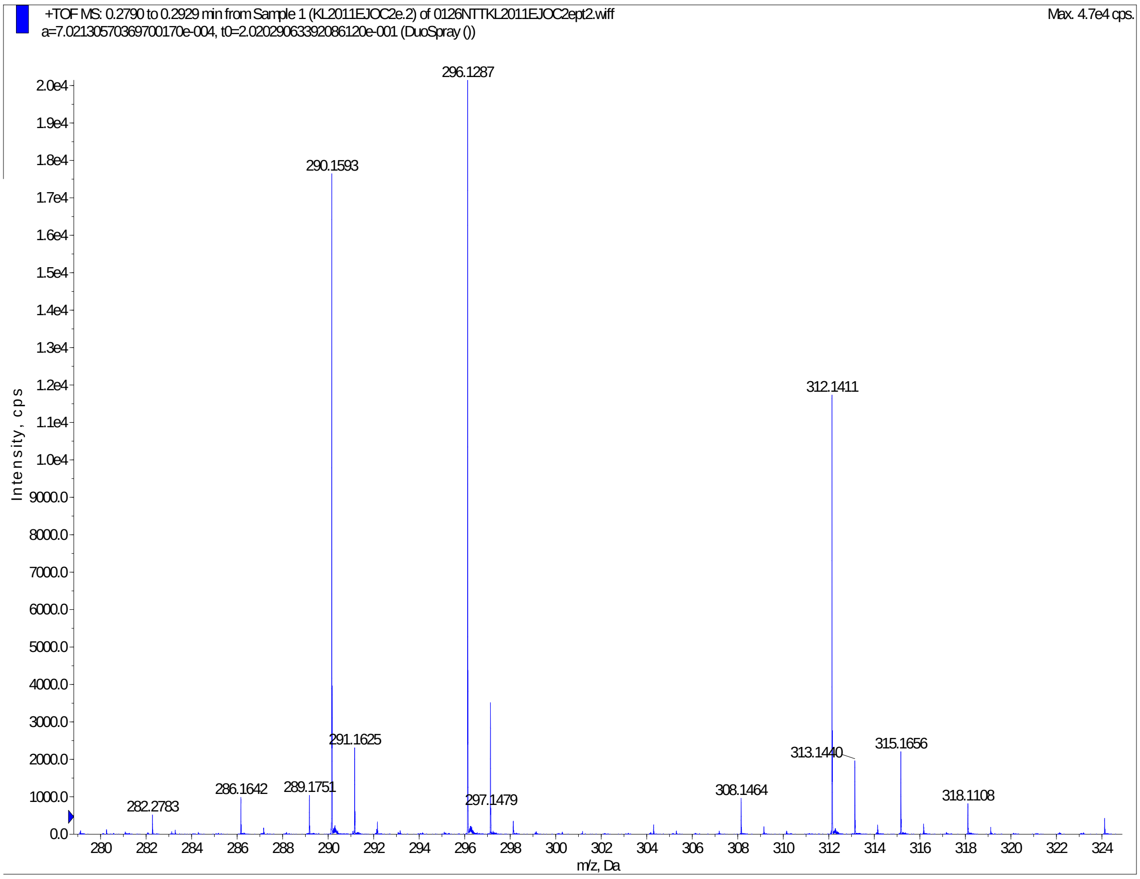
+TOF MS: 0.2790 to 0.2929 min from Sample 1 (KL2011EJOC2e.2) of 0126NTTKL2011EJOC2ept2.wiff
a=7.02130570369700170e-004, t0=2.02029063392086120e-001 (DuoSpray ())
Max. 4.7e4 cps.
Intensity, cps
m/z, Da
296.1287
290.1593
312.1411
291.1625
313.1440
315.1656
286.1642
289.1751
282.2783
297.1479
308.1464
318.1108
